# Supplementary material for: Ex vivo model of functioning human lymph node reveals role for innate lymphocytes and stroma in response to vaccine adjuvant
Source: Cell Rep. 2025 Jul 2;44(7):115938. doi: 10.1016/j.celrep.2025.115938 (PMC12284374; doi:10.1016/j.celrep.2025.115938)
Supplement: Document S1. Figures S1–S5, Tables S1 and S6–S9, and Methods S1 [file mmc1.pdf]

Cell Reports, Volume *44*

Supplemental information

Ex vivo model of functioning human lymph node reveals role for innate lymphocytes and stroma in response to vaccine adjuvant

Joannah R. Fergusson, Jacqueline H.Y. Siu, Nitya Gupta, Edward Jenkins, Eloise Nee, Sören Reinke, Tamara Ströbel, Ananya Bhalla, Shyami M. Kandage, Thomas Courant, Sarah Hill, Moustafa Attar, Michael L. Dustin, Alex Gordon-Weeks, Mark Coles, Calliope A. Dendrou, and Anita Milicic

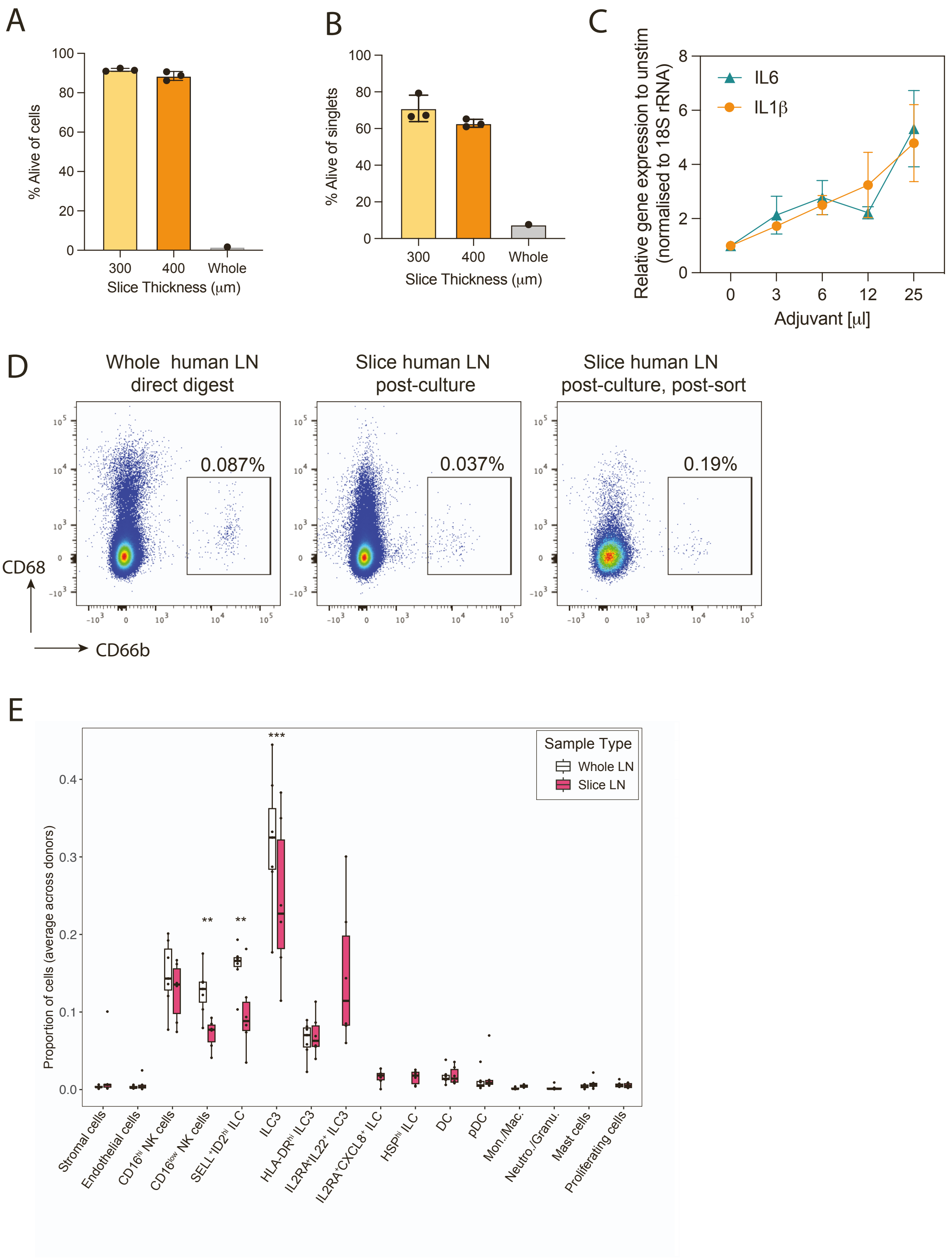

Figure S1: Viability and responsiveness of LN slices in culture. Related to Figure 1.
(A) Percentage of live cells (L/D negative amongst cells gated on FSC vs SSC) from pig LN slices of indicated thickness compared to cultured whole LN after 20 h culture. (n=3 for slices, n=1 whole LN). **(B)** Percentage of live cells (L/D negative amongst all events, gated on singlets only) from pig LN slices of indicated thickness compared to cultured whole LN after 20 h culture (n=3 for slices, n=1 whole LN). **(C)** Expression of IL6 and IL1β relative to housekeeping 18S rRNA in pig LN slices stimulated with the indicated volumes of adjuvant in 1ml of media after 20 h of culture (n=3, average of duplicate wells). **(D)** Presence of neutrophils (gated on singlets; live cells; CD45+CD66b+) in single cell suspensions from human whole LN or sliced LN after 20 h culture pre- (middle) or post- sorting (right). Percentage of CD66b+ in CD45+ cells is indicated above each gate (representative of 3 donors). **(E)** Proportion of each cell type (excluding T and B cells) in single cell transcriptomic analyses of human whole LN (black outline) and LN slices (magenta), each point represents an individual donor. **p≤0.005, ***p<0.0005 by ANOVA using a linear model between cell types with matched clusters in whole and slice datasets.

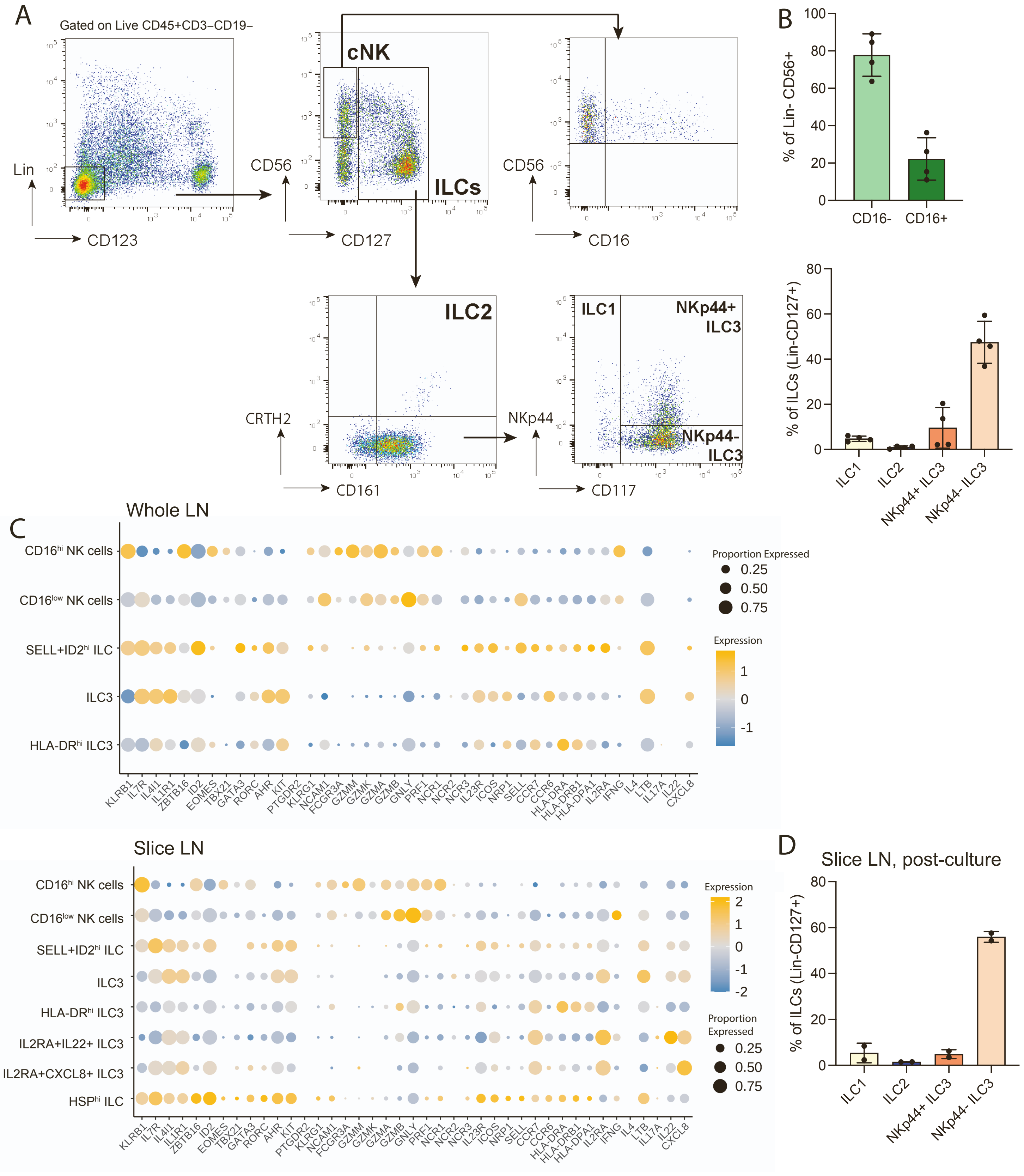

Figure S2: ILC populations of the human cystic LN. Related to Figure 1.
(A) Gating strategy for the identification of NK cells and ILC subsets from whole human LN by flow cytometry. Lin = CD14, CD66, CD34. **(B)** Quantification of the percentage of NK cells either CD16− or CD16+ (above) and ILC subsets (below), as gated in A. **(C)** Dot plots showing expression of marker genes for each ILC cluster within whole (above) and slice (below) human LN. Colour indicates relative log-normalised level of expression across clusters and dot size the proportion of each cluster expressing each gene. **(D)** Quantification of the percentage of ILC subsets in slice human LN following 20 h culture by flow cytometry, as in B.

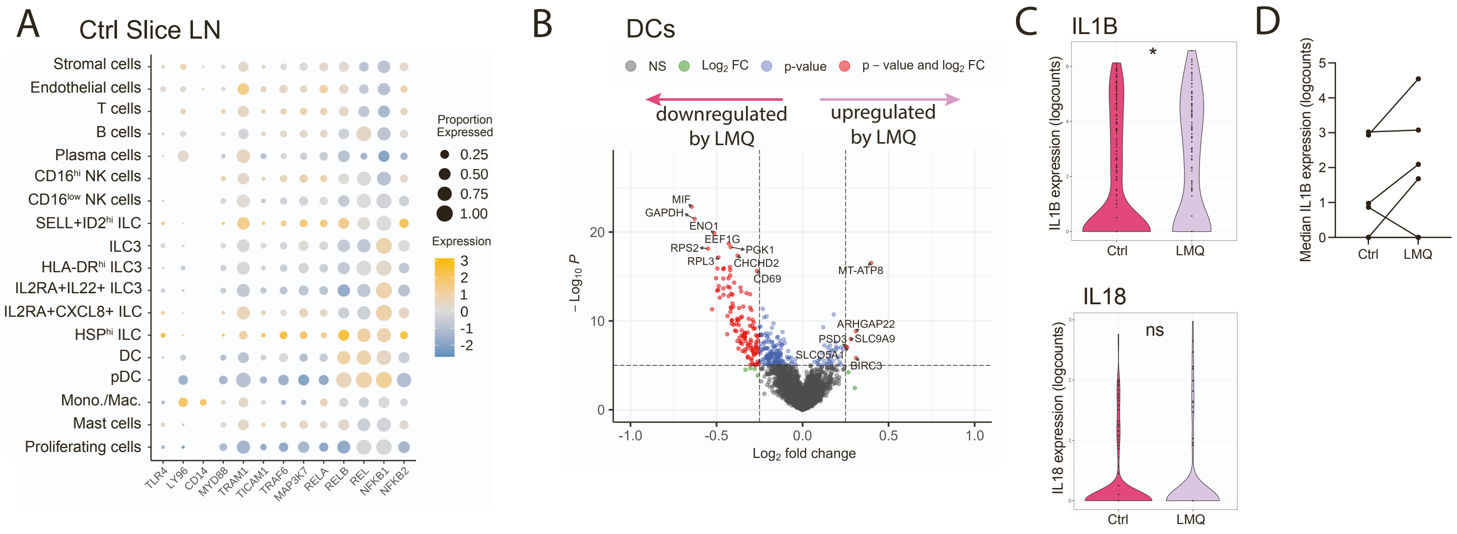

Figure S3: LN slice responses to TLR4 and inflammasome activation by adjuvant LMQ. Related to Figure 3.
(A) Dot plot showing expression of TLR4 signalling components for each cell cluster within untreated (Ctrl) LN slices. Colour indicates relative log-normalised level of expression across clusters and dot size the proportion of each cluster expressing each gene. **(B)** Volcano plot indicating differentially expressed genes between LMQ-stimulated (right: upregulated) and control (left: upregulated) conditions in DCs. Genes with $\log_2$ fold-change >0.25 are indicated in green, those with p value $<10^{-5}$ in blue and those fulfilling both criteria in red. **(C)** Log normalised expression levels of IL1B and IL18 transcripts in the Monocytes/Macrophages cluster of all donors in control (magenta) and LMQ (lavender) treated samples. *p<0.05. **(D)** Median log normalised expression levels of IL1B transcripts in the Monocytes/Macrophages cluster of paired donor slice conditions, either control or LMQ treated conditions.

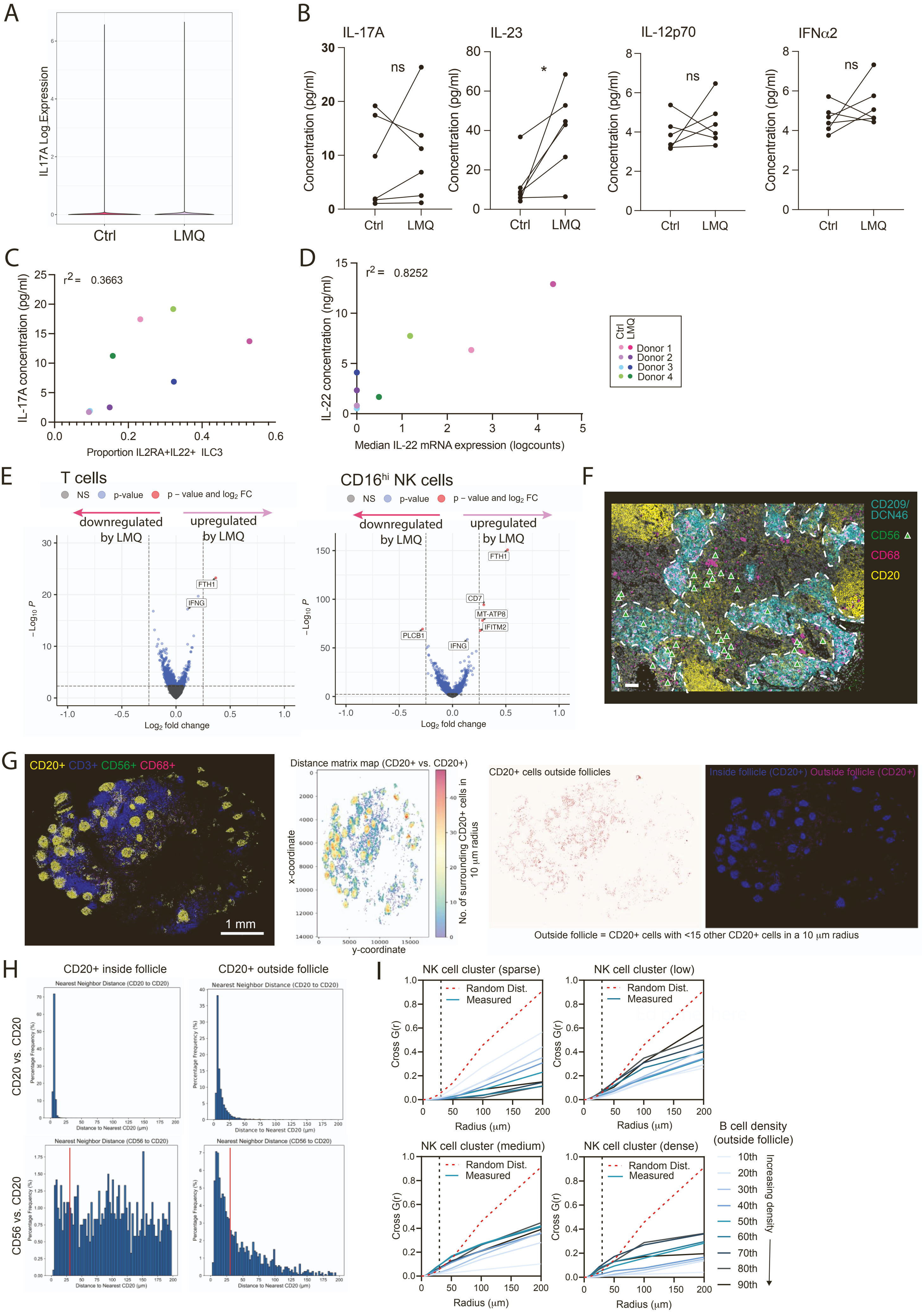

A
IL17A Log Expression
Ctrl
LMQ
B
IL-17A
IL-23
IL-12p70
IFNα2
Concentration (pg/ml)
ns
*
ns
ns
Ctrl
LMQ
C
r2 = 0.3663
IL-17A concentration (pg/ml)
Proportion IL2RA+IL22+ ILC3
D
r2 = 0.8252
IL-22 concentration (ng/ml)
Median IL-22 mRNA expression (logcounts)
Ctrl
LMQ
Donor 1
Donor 2
Donor 3
Donor 4
E
T cells
CD16hi NK cells
NS
p-value
p – value and log2 FC
downregulated by LMQ
upregulated by LMQ
FTH1
IFNG
CD7
MT-ATP8
IFITM2
PLCB1
Log2 fold change
F
CD209/ DCN46
CD56
CD68
CD20
G
CD20+ CD3+ CD56+ CD68+
1 mm
Distance matrix map (CD20+ vs. CD20+)
x-coordinate
y-coordinate
No. of surrounding CD20+ cells in 10 μm radius
CD20+ cells outside follicles
Inside follicle (CD20+) Outside follicle (CD20+)
Outside follicle = CD20+ cells with <15 other CD20+ cells in a 10 μm radius
H
CD20+ inside follicle
CD20+ outside follicle
CD20 vs. CD20
CD56 vs. CD20
Nearest Neighbor Distance (CD20 to CD20)
Nearest Neighbor Distance (CD56 to CD20)
Percentage Frequency (%)
Distance to Nearest CD20 (μm)
I
NK cell cluster (sparse)
NK cell cluster (low)
NK cell cluster (medium)
NK cell cluster (dense)
Random Dist.
Measured
Cross G(r)
Radius (μm)
B cell density (outside follicle)
10th
20th
30th
40th
50th
60th
70th
80th
90th
Increasing density

Figure S4: Activation of ILCs by adjuvant and downstream signaling to B cells. Related to Figure 4.
(A) Log normalised expression levels of IL17A transcripts in all donors in control (magenta) and LMQ (lavender) treated samples. **(B)** Concentrations of IL-17A, IL-23, IL-12p70 and IFNα2 in slice culture supernatants after 20 h culture without (Ctrl) or with LMQ adjuvant (LMQ) from paired donor conditions (n=6, each point is the average of 2-4 slices from one donor *p<0.05 or ns = not significant by Wilcoxon matched-pairs signed rank test). **(C)** Proportion of IL2RA+IL22+ ILC3 cells per donor and per condition against IL-17A concentration detected in culture supernatants. Dots are coloured according to donor, and shade according to treatment (light = control, dark = LMQ-treated). R^2 value was calculated by simple linear regression. **(D)** Median log-normalised expression of IL-22 in the IL2RA+IL22+ ILC3 cluster per donor and per condition against IL-22 concentration detected in culture supernatants. Dots are coloured according to donor, and shade according to treatment (light = control, dark = LMQ-treated). R^2 value was calculated by simple linear regression. **(E)** Volcano plot indicating differentially expressed genes between LMQ-stimulated (right: upregulated) and control (left: upregulated) conditions in T cells (left) and $CD16^{hi}$ NK cells (right). Genes with log_2 fold-change >0.25 are indicated in green, those with p value $<10^{-5}$ in blue and those fulfilling both criteria in red. **(F)** Multiplexed stained image of whole human LN for NK cells (CD56, green triangles) in relation to medullary sinuses (CD209/DCN46; dashed line). Macrophages (CD68) and B cells (CD20) are also shown; scale bars are 50 μm. Images are representative of three individuals. **(G)** (Left) Image of segmented nuclei coloured by indicated marker. Scale bar is indicated. (Middle) Distance map of CD20+ cells from segmented image left, coloured based on the number of surrounding CD20+ cells within a 10 μm radius. Red and blue regions demarcate dense and sparse areas of CD20+ cells, respectively. (Right) CD20+ cells with <15 other CD20+ cells within a 10 μm radius were considered outside follicles. (Right) Nuclei coloured according to this threshold, with blue and magenta nuclei indicating CD20+ inside and outside follicles, respectively. **(H)** Nearest-neighbourhood analysis of CD20+ cells inside and outside follicles vs. either other CD20+ cells (inside follicles) or vs. CD56+ cells (NK cells). Red line indicates 30 μm distance. **(I)** Cross-G analysis of NK and CD20+ cells (outside follicles) after stratifying NK and B cells into groups based on their local density. NK cells were stratified into four distinct cluster sizes ('sparse', 'low', 'medium' and 'dense' based on probability density quartiles) and B cells into ten (one being sparse, and ten being dense). Red-dashed line is the theoretical likelihood of a cell cluster being nearby for a given radius from the cell of interest. This is based on a Poisson distribution and uses the measured area of the LN (see Methods). The teal line indicates the empirical value from the image. Empirical > theoretical = cells examined are closer than you would expect by random. Empirical < theoretical = cells further apart than expected by random. Empirical = theoretical = randomly distributed. This highlights whether sparse or dense NK/B cells tended to congregate more than expected by random. Figure shows that only dense NK and B cell populations tended to congregate closer than expected by random. Analysis is based on using distance matrices from 33,082 CD20+ total, 24,785 CD20+ inside follicles, 8297 CD20+ outside follicles and 1201 CD56+ cells total. 300 cells were used per NK quartile (i.e., sparse, low, medium, dense). ~830 cells were used per CD20+/B cell decile.

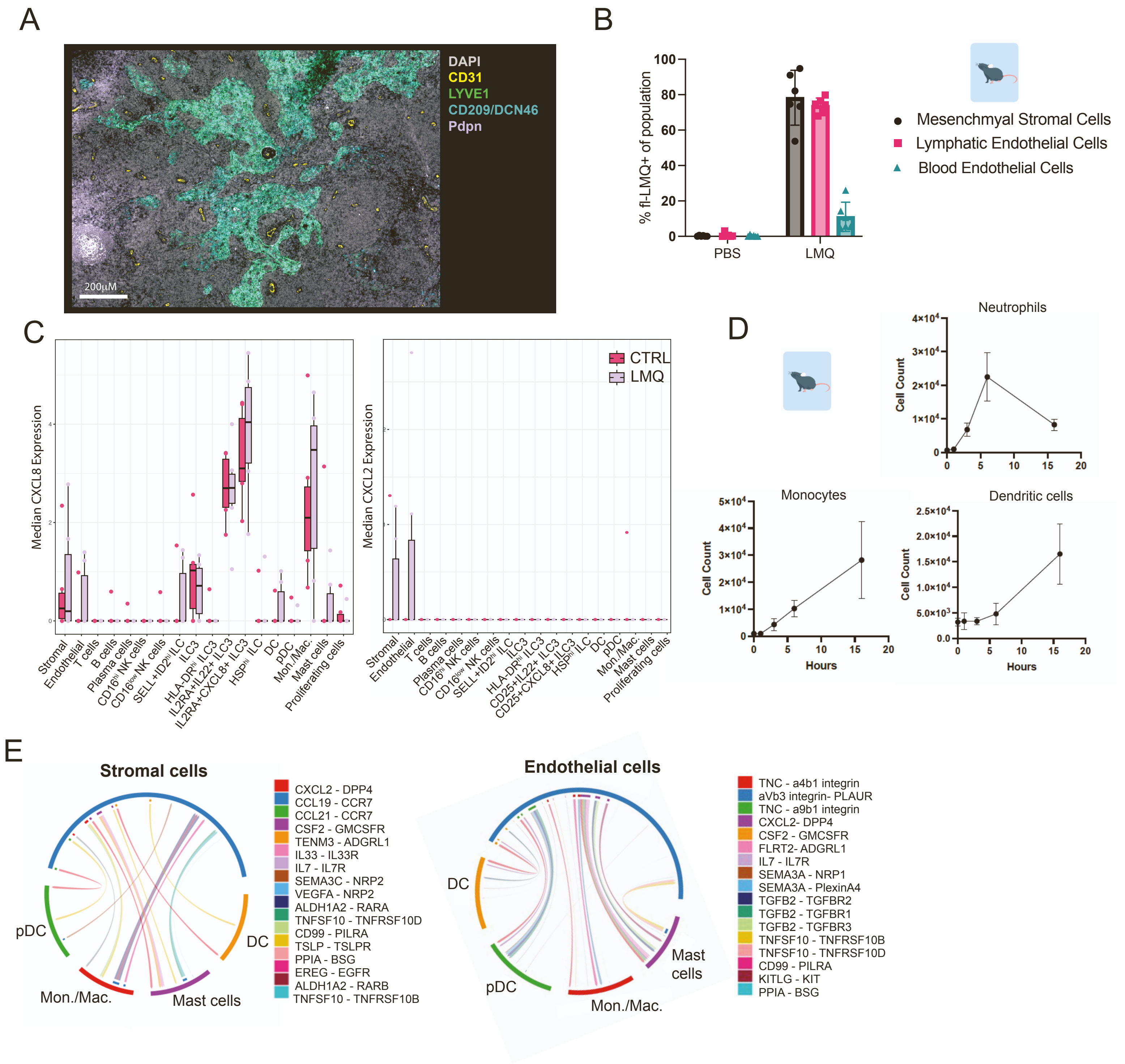

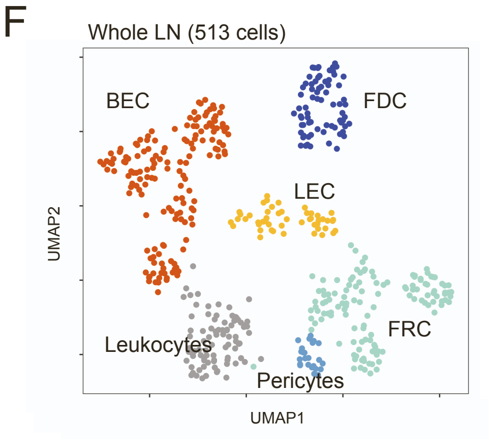

Figure S5: Recruitment of innate cells by non-hematopoietic stromal cells (NHSCs). Related to Figure 5.
(A) Multiplex stained image of a human LN slice after 20 h in culture with LMQ adjuvant spatially identifying endothelial (LYVE1, CD31, CD209) and stromal (Podoplanin; Pdpn) populations. Scale bars are indicated. Image is representative of results from two donors. **(B)** Proportion of adjuvant positive cell populations within CD45– populations of mouse draining LN (dLN) following i.m. injection of fluorescent LMQ adjuvant after 24h. Stromal cells were defined as CD45–Pdpn+CD31– and endothelial cells as CD45–CD31+ and either Pdpn+ (Lymphatic Endothelial cells) or Pdpn– (Blood endothelial cells). Bars indicate mean with standard deviation. **(C)** Median CXCL8 (left) and CXCL2 (right) transcript expression across cell types in human LN slices without (Ctrl; magenta) or with LMQ adjuvant (LMQ; lavender), each dot represents an individual donor. **(D)** Absolute cell counts of neutrophils, monocytes and dendritic cells in the mouse dLN at various timepoints following i.m. injection of LMQ adjuvant (n=6). **(E)** Relevant interactions upregulated in response to adjuvant stimulation between stromal (left) and endothelial (right) cells and indicated innate cell types as calculated by CellPhoneDB. Arrows in the chord diagram indicate the direction of the interaction, with coloured bars indicating the sender cell type and the colour of the bar the receiver cell type. **(F)** UMAP plot of NHSCs from whole human LN coloured by cluster and stromal cell type.

Table S1: Human LN donor demographics

Donor	Self-declared Ethnicity	Age	Sex
1	White British	20	Female
2	White British	61	Female
3	White Eastern European	45	Male
4	White British	59	Male
5	White British	49	Female
6	White Eastern European	40	Male
7	White British	64	Female
8	White British	54	Female
9	White British	54	Female
10	Asian	51	Female
11	White other	75	Female

Table S6: General reagents used throughout study

REAGENT	SOURCE	IDENTIFIER
LMQ	Vaccine Formulation Initiative	DOI: 10.3389/fimmu.2022.976968
RPMI 1640 Medium	ThermoFisher Scientific (Gibco)	Cat#: 21875-034
Dispase II, powder	ThermoFisher Scientific	Cat#: 17105-041
Collagenase P	Merck	Cat#: 11213857001
Deoxyribonuclease I from bovine pancreas	Sigma Aldrich	Cat#: D5025-150KU
PBS, pH 7.4	ThermoFisher Scientific (Gibco)	Cat#: 10010015
Fetal Bovine Serum, Value, One Shot™ format	ThermoFisher Scientific (Gibco)	Cat#: A5209401
UltraPure 0.5M EDTA, pH 8.0	ThermoFisher Scientific (Invitrogen)	Cat#: 15575020
1,1' – Dioctadecyl-3,3,3'3'-Tetramethylindodicarbocyanine Perchlorate (DiD'/DiIC$_{18}$(5)) oil	ThermoFisher Scientific	Cat#:D307
Digitonin (5%)	ThermoFisher Scientific	Cat#: BN2006
UltraPure Agarose	ThermoFisher Scientific (Invitrogen)	Cat#: 16500500
Penicillin-Streptomycin (10,000 U/ml)	ThermoFisher Scientific (Gibco)	Cat#: 15140122
2-Mercaptoethanol	VWR Lifescience	Cat#: 0482-100ML
Sodium Pyruvate (100mM)	ThermoFisher Scientific	Cat#: 11360070
MEM Non-Essential Amino Acids Solution (100X)	ThermoFisher Scientific	Cat#: 11140035
HEPES (1M)	ThermoFisher Scientific (Gibco)	Cat#: 15630056
CP-456773 sodium salt (MCC950)	Sigma Aldrich	Cat#: PZ0280
TLR4 inhibitor, TAK242	Merck	Cat#: 614316
LEGENDplex Human Inflammation Panel 1	BioLegend	Cat#: 740809
Human IL-22 ELISA Kit - Quantikine	R&D Systems	Cat#: D2200
eBioscience Cell Stimulation Cocktail (500X)	ThermoFisher Scientific (Invitrogen)	Cat#: 00-4970-93

Table S7: Flow cytometry Antibodies, Instrument, Software

REAGENT	SOURCE	IDENTIFIER	
**Antibodies**		**Clone**	**Cat. No.**
Brilliant Violet 795 anti-mouse CD45 Antibody	BioLegend	30-F11	103149
CD45R (B220) Monoclonal Antibody, Alexa Fluor 488	eBioscience	RA3-6B2	53-0452-82
Alexa Fluor 594 anti-mouse F4/80 Antibody	BioLegend	BM8	123140
PE/Cyanine7 anti-mouse CD169 (Siglec-1) Antibody	BioLegend	3D6.112	142411
Ly6G/Ly-6C Monoclonal Antibody, eFluor450, eBiosience	ThermoFisher Scientific	RB6-8C5	48-5931-82
Brilliant Violet 605 anti-mouse CD11c Antibody	BioLegend	N418	117334
Brillaint Violet 711 anti-mouse/human CD11b Antibody	BioLegend	M1/70	101241
Alexa Fluor 488 anti-mouse CD3 Antibody	BioLegend	17A2	100212
CD19 Monoclonal Antibody, FITC, eBioscience	ThermoFisher Scientific	MB19-1	11-0191-85
Alexa Fluor 488 anti-mouse NK1.1 Antibody	BioLegend	PK136	108718
PerCP/Cyanine5.5 anti-mouse I-A/I-E Antibody	BioLegend	M5/114.15.2	107626
PE anti-mouse Ly-6C Antibody	BioLegend	HK1.4	128008
BD Horizon BUV395 Mouse Anti-Human CD3	BD Biosciences	UCHT1	563548
BD Horizon BUV496 Mouse Anti-Human CD45	BD Biosciences	HI30	750179
Brillaint Violet 421 anti-human CD19 Antibody	BioLegend	HIB19	302233
BD OptiBuild BV750 Mouse Anti-Human CD25 (IL-2 Receptor α)	BD Biosciences	2A3	747290
BD OptiBuild BV786 Mouse Anti-Human CD161	BD Biosciences	HP-3G10	748281
PerCP/Cyanine5.5 anti-human CD14 Antibody	BioLegend	HCD14	325621
Alexa Fluor 488 anti-human CD56 (NCAM) Antibody	BioLegend	HCD56	318311
PE anti-human CD117 (c-kit) Antibody	BioLegend	104D2	313203
PE/Dazzle 594 anti-human CD294 (CRTH2) Antibody	BioLegend	BM16	350125
PE/Cyanine5 anti-human CD16 Antibody	BioLegend	3G8	302009
PE/Cyanine7 anti-human CD123 Antibody	BioLegend	6H6	306009
Alexa Fluor 647 anti-human CD336 (NKp44) Antibody	BioLegend	P44-8	325112
Alexa Fluor 700 anti-human CD66b Antibody	BioLegend	G10F5	305113
Alexa Fluor 700 anti-human CD34 Antibody	BioLegend	561	343621
APC/Fire 810 anti-human CD127 (IL-7Rα) Antibody	BioLegend	A019D5	351374
Alexa Fluor 700 anti-human CD16 Antibody	BioLegend	3G8	302026
PE/Cyanine7 anti-human CD11c Antibody	BioLegend	S-HCL-3	371508
PE anti-human Podoplanin Antibody	BioLegend	NZ-08	337003
Alexa Fluor 488 anti-human CD3 Antibody	BioLegend	HIT3a	300319
Alexa Fluor 488 anti-human CD19 Antibody	BioLegend	HIB19	302219
Brilliant Violet 785 anti-human CD68 Antibody	BioLegend	Y1/82A	333826
BD OptiBuild BV650 Anti-Human CD31	BD Biosciences	M89d3	744465
Brilliant Violet 605 anti-human CD45 Antibody	BioLegend	HI30	304042
PE anti-human CD235a (Glycophorin A) Antibody	BioLegend	I264	349105
BD Horizon BV421 Mouse Anti-Human HLA-DR	BD Biosciences	G46-6	562805
**Other**			
Live/Dead Fixable Near-IR Dead Cell Kit	ThermoFisher Scientific	Cat#: L10119	
Live/Dead Fixable Aqua Dead Cell Kit	ThermoFisher Scientific	Cat#: L34957	
7-AAD Viability Staining Solution	BioLegend	Cat#: 420403	
Human TruStain FcX Fc Receptor Blocking Solution	BioLegend	Cat#: 422302	
TruStain FcX (anti-mouse CD16/32) Antibody	BioLegend	Cat#: 101320	
ArC Amine Reactive Compensation Bead Kit	ThermoFisher Scientific	Cat#: A10628	
AbC Total Antibody Compensation Kit	ThermoFisher Scientific	Cat#: A10497	
BD CompBeads Anti-Mouse Ig, k/Negative Control Compensation Particles Set	BD Biosciences	Cat#: 552843	
Precision Count Beads	BioLegend	Cat#: 424902	
Fixation Buffer	BioLegend	Cat#: 420801	
eBioscience Foxp3/Transcription Factor Staining Buffer Set	ThermoFisher Scientific	Cat#: 00-5523-00	
**Software**			
FlowJo v10.8.1	BD Life Sciences	https://docs.flowjo.com/flowjo/getting-acquainted/10-8-release-notes/10-8-1-exhaustive-release-notes/	
**Hardware**			
LSRFortessa X20 (R/B/V/YG)	BD Biosciences		
Cytek Aurora (R/B/V/YG/UV)	Cytek Biosciences		

Table S8: CellDIVE Antibodies, Instrument, Software

REAGENT	SOURCE	IDENTIFIER	
**Antibodies**		**Clone**	**Cat. No.**
Recombinant Anti-NCAM1 Antibody	Abcam	EPR2566	ab133345
IL3RA/CD123 Mouse mAb	Cell Signaling Technology	BR4MS	23797S
Purified anti-human IgD Antibody	BioLegend	W18340A	324502
Mast Cell Tryptase Antibody	Santa Cruz	AA1	sc-59587
Recombinant Alexa Fluor 555 Anti-CD68 Antibody	Abcam	EPR20545	ab280860
Alexa Fluor 647 anti-human CD66b Antibody	BioLegend	G10F5	305110
Alexa Fluor 647 Anti-Sialoadhesin/CD169 antibody	Abcam	SP216	ab306568
Alexa Fluor 488 Anti-CD11c Antibody	Abcam	EP1347Y	ab275738
Alexa Fluor 555 Anti-CD3 Antibody	Abcam	SP162	ab307335
CD31/PECAM-1 Antibody	Novus Biologicals	C31.3	NBP2-15202
Alexa Fluor 488 Anti-CD20 Antibody	Abcam	EP459Y	ab198941
Purified Mouse Anti-Human CD209	BD Biosiences	DCN46	551186
Podoplanin Mouse Monoclonal Antibody	Cell Marque	D2-40	322M
Recombinant Anti-CD14 Antibody	Abcam	EPR3653	ab133335
Recombinant Anti-LYVE1 Antibody	Abcam	EPR21857	ab232935
Donkey Anti-Rabbit IgG H&L	Abcam	Polyclonal	ab150073
Donkey Anti-Mouse IgG H&L	Abcam	Polyclonal	ab150106
Donkey Anti-Rat IgG H&L	Abcam	Polyclonal	ab150155
**Other**			
Bovine Serum Albumin (BSA)	Sigma – Aldrich	Cat#: A7906	
Citrate Antigen Retrieval Solution (ARS1)	Vector Laboratories	Cat#: H-3300	
DAPI Stock Solution	ThermoFisher Scientific	Cat#: D3571	
Donkey Serum	Sigma – Aldrich	Ca#: D9663	
EDTA	Alfa Aesar	Cat#: 33312	
Ethanol	Honeywell	Cat#: 32221-2.5L	
FcR Blocking Reagent	Miltenyi	Cat#: 130-059-901	
Human TruStain FcX Fc Receptor Blocking Solution	BioLegend	Cat#: 422302	
Glycerol	Sigma – Aldrich	Cat#: G5516	
Hydrogen Peroxide	Sigma – Aldrich	Cat#: 216763	
Phosphate Buffered Saline 10X	VWR	Cat#: 437117K	
Propyl Gallate	Sigma – Aldrich	Cat#: 02370	
Sodium Bicarbonate	Sigma - Aldrich	Cat #: S6297	
Sodium Hydroxide Pellets	Sigma – Aldrich	Cat#: 30620-1KG-M	
Triton X-100	Sigma – Aldrich	Cat#: T9284	
Trizma®Base	Sigma – Aldrich	Cat#: T6066	
Xylene	Sigma – Aldrich	Cat#: 534056	
Super Frost Plus Glass Slides	Fisher Scientific	Product code 10149870	
Leica SurgiPath Coverglass 24 x 50 mm coverslips (Size 1)	Leica	Cat#: 3800145G	
Mix-n-Stain CF450 Dye Antibody Labeling Kit (50-110ug)	Biotium	Cat#: 92324	
Mix-n-Stain CF555 Dye Antibody Labeling Kit (50-110ug)	Biotium	Cat#: 92234	
**Software**			
QuPath v0.4.3	Bankhead, P. et al. QuPath: Open source software for digital pathology image analysis. Scientific Reports (2017). https://doi.org/10.1038/s41598-017-17204-5	https://qupath.github.io/	
**Hardware**			
Cell DIVE multiplexed imaging platform	General Electric	https://www.ge.com/research/project/multiplexed-tissue-imaging-platform	
Epredia Gemini AS Automated Slide Stainer	Fisher Scientific	https://www.fishersci.co.uk/shop/products/gemini-as-automated-slide-stainer/15243973	
Manual Rotary Microtome HistoCore BIOCUT	Leica	https://www.leicabiosystems.com/en-gb/histology-equipment/microtomes/histocore-biocut/	

Table S9: qPCR primers and reagents

Gene symbol	Gene	Forward primer	Reverse primer	Manufacturer
18S rRNA	18S ribosomal RNA	GACAAATCGCTCCACCAACT	CCTGGGGCTTAATTTGACTC	ThermoFisher Scientific
IL1B	Interleukin 1b	TGATGGCTAACTACGGTGACAACA	CTCAGAGAACCAAGGTCCAGGTTT	
IL6	Interleukin 6	CTCTCCACAAGCGCCTTTAGTC	GCATTTTGTCTGAGGTGGCATC	

REAGENT	SOURCE	IDENTIFIER
SYBR Green PCR Master Mix	ThermoFisher Scientific	Cat#: 4309155
First Strand cDNA Synthesis Kit	ThermoFisher Scientific	Cat#: K1612
RNeasy Mini Kit	Qiagen	Cat#: 74106
RNase-Free DNase Set	Qiagen	Cat#: 79254
**SOFTWARE**		
Design & Analysis Software 2.6.0	AppliedBioSystems by ThermoFisher Scientific	
**HARDWARE**		
ViiA 7 Real-Time PCR System	ThermoFisher Scientific	

METHODS S1: Methods relating to supplementary material only

Pig Samples

Porcine mesenteric lymph nodes from healthy piglets were kindly provided by The Pirbright Institute, and were processed as for human lymph nodes.

CellDIVE Image Quantification

Segmentation and subsequent extraction of centroid, shape descriptors (e.g., area, perimeter) and all channel intensity information of individual nuclei was performed using StarDist in python with the pre-trained '2d_versatile_flou' model [1] and 'regionprops' from Skicit-image [2], respectively. 249,227 nuclei were detected. Nuclei were assigned a positive marker if the intensity was greater than the median + 1-3.5x the standard deviation for that intensity across all nuclei. More stringent thresholds were assigned to channel CD56 based on weaker staining compared to CD3 or CD20. Only nuclei that were single positive for a given marker were used for downstream analysis. For example, nuclei that were CD3+CD56+ (i.e., considered as NKT cells) were excluded. CD3+ only were defined as T cells, CD20+ cells were defined as B cells and CD56+ cells were defined as NK cells. To separate CD20+ cells inside vs. outside of follicles a distance matrix (i.e., using the Euclidian distance) was produced between all nuclei, and CD20+ cells that exhibited > 15 other CD20+ cells with a 10 μm radius were considered inside a follicle. The (cross) G-function (i.e., cumulative frequency distribution (CFD) of the 1st-order nearest neighbour distances), was used to produce a probability curve for the spatial relationships between indicated cells [3]. The cross G-function is given by:

$$G^{xy}(r) = \frac{1}{N_x}\sum_{k=1}^{N_x} 1\{d_k^{(y)} \leq r\}$$

To provide context, the CFD is compared to a curve based on a Poisson distribution (i.e., a random distribution of cells) within the same given area of the LN (i.e., 15493173 μm^2 in this case). The Poisson distribution is given by:

$$G_{xy}(r) = 1 - e^{-\lambda_y \pi r^2}$$

λ_y is the intensity (mean density) of cell type-y. Clustered populations of cells appear as curves shifted to the left of the Poisson distribution, and is interpreted as cell population x and cell population y being found closer more than you would expect by random for the same given number of cells within a defined area. Curves that match the Poisson distribution are randomly distributed. Curves that fall to the right are likely cells segregated from each other. NK cells and B cells (outside follicles) were further stratified into different density groups i.e., 'sparse', 'low', 'medium', and 'dense' (for NK cells) or density groups 1-10 (1 being sparse and 10 being most dense) for B cells. This was done using quartiles/deciles of probability density values

measured from a gaussian kernel density estimate of the NK or B cell (outside follicle) centroid coordinates. All image processing and quantification was performed using custom scripts written in Python, and are available at DOI 10.5281/zenodo.15396757.

Quantitative real-time polymerase chain reaction (qRT-PCR)

Slices were stored in RNA protect (Qiagen) until RNA extraction by RNeasy Mini Kit (Qiagen) following manufacturer's instructions. An electronic pestle was used to aid tissue disruption. Extracted RNA was reverse transcribed using First-strand cDNA synthesis kit (Thermo Fisher Scientific) and 100ng of complementary DNA used in a 20μl qRT-PCR reaction. PCR cycling consisted of 20 seconds at 95°C followed by 40 cycles of 3 seconds at 95°C followed by 20 seconds at 60°C. Amplified DNA products were detected by SYBR Green PCR Master Mix (ThermoFisher Scientific) and primers for IL1B, IL6 and 18S rRNA and with a ViiA 7 Real-Time PCR System (Thermo Fisher Scientific). The reagents, instruments, and software used are listed in Table S9.

CellPhoneDB

To analyse cell-cell communication molecules, CellPhoneDB v5 (method 3) was used to understand the differentially expressed interactions. Relevant interactions were determined by: 1) interaction genes of interest must be expressed in the corresponding cell type by more than 10% of cells, and 2) at least one gene-cell type pair is identified in the DEG analysis described above. Interactions were also scored as described by CellPhoneDB v5 to rank interactions by specificity of their interacting partners [4,5] . Results were visualised using ktplots. Relevant interactions and significant means are detailed in Table S5.

SUPPLEMENTARY REFERENCES

1. Schmidt, U., Weigert, M., Broaddus, C., and Myers, G. (2018). Cell Detection with Star-Convex Polygons. In Lecture Notes in Computer Science, (Springer International Publishing), pp. 265-273. 10.1007/978-3-030-00934-2_30.
2. van der Walt, S., Schonberger, J.L., Nunez-Iglesias, J., Boulogne, F., Warner, J.D., Yager, N., Gouillart, E., Yu, T., and scikit-image, c. (2014). scikit-image: image processing in Python. PeerJ *2*, e453. 10.7717/peerj.453.
3. Parra, E.R. (2021). Methods to Determine and Analyze the Cellular Spatial Distribution Extracted From Multiplex Immunofluorescence Data to Understand the Tumor Microenvironment. Frontiers in Molecular Biosciences *8*. 10.3389/fmolb.2021.668340.
4. Efremova, M., Vento-Tormo, M., Teichmann, S.A., and Vento-Tormo, R. (2020). CellPhoneDB: inferring cell-cell communication from combined expression of multi-subunit ligand-receptor complexes. Nat Protoc *15*, 1484-1506. 10.1038/s41596-020-0292-x.

5. Troule, K.P., R.; Prete, M.; Cranley, J.; Harasty, A.; Tuong, Z.K.; Teichmann, S.A.; Garcia-Alonso, L.; Vento-Tormo, R. (2023). CellPhoneDB v5: inferring cell-cell communication from single-cell multiomics data. arXiv. 10.48550/arXiv.2311.04567.
