# Supplementary material for: Ex vivo model of functioning human lymph node reveals role for innate lymphocytes and stroma in response to vaccine adjuvant
Source: Cell Rep. 2025 Jul 2;44(7):115938. doi: 10.1016/j.celrep.2025.115938 (PMC12284374; doi:10.1016/j.celrep.2025.115938)
Supplement: Document S2. Article plus supplemental information [file mmc6.pdf]

Cell Reports

Article

Ex vivo model of functioning human lymph node reveals role for innate lymphocytes and stroma in response to vaccine adjuvant

Graphical abstract

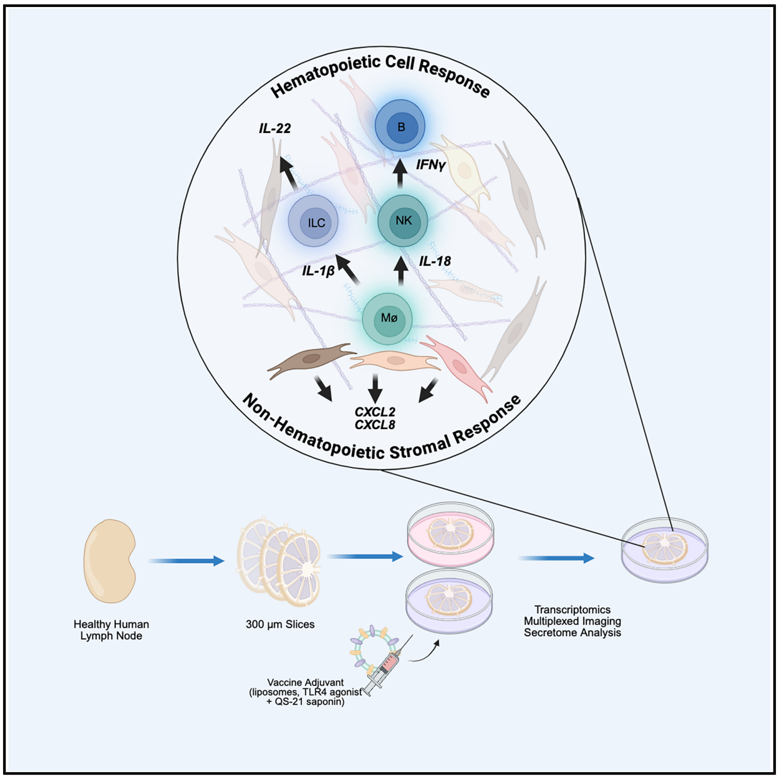

Authors

Joannah R. Fergusson, Jacqueline H. Y. Siu, Nitya Gupta, ..., Mark Coles, Calliope A. Dendrou, Anita Milicic

Correspondence

mark.coles2@kennedy.ox.ac.uk (M.C.),
calliope.dendrou@kennedy.ox.ac.uk (C.A.D.),
anita.milicic@ndm.ox.ac.uk (A.M.)

In brief

Fergusson et al. establish precision cutting and culture of human lymph nodes for studying response to perturbation in architecturally preserved, functioning, lymphoid tissue. In response to stimulation with a vaccine adjuvant, the model reveals a central role for innate lymphoid cells in directing adaptive immunity, with stromal cells orchestrating the inflammatory response.

Highlights

- Precision-cut human LNs remain functionally responsive to inflammatory stimuli in culture
- Vaccine adjuvant drives an *ex vivo* cascade of direct and cytokine-mediated signaling
- ILCs including NK cells are a major cell type in the LN, activated indirectly by macrophages
- Resident stromal cells are key for neutrophil recruitment in response to the adjuvant

Fergusson et al., 2025, Cell Reports *44*, 115938
July 22, 2025 © 2025 The Authors. Published by Elsevier Inc.
https://doi.org/10.1016/j.celrep.2025.115938

CellPress

Cell Reports

CellPress
OPEN ACCESS

Article

Ex vivo model of functioning human lymph node reveals role for innate lymphocytes and stroma in response to vaccine adjuvant

Joannah R. Fergusson,[1] Jacqueline H.Y. Siu,[1] Nitya Gupta,[1] Edward Jenkins,[1] Eloise Nee,[1] Sören Reinke,[2] Tamara Ströbel,[1] Ananya Bhalla,[1] Shyami M. Kandage,[1] Thomas Courant,[3] Sarah Hill,[1] Moustafa Attar,[1] Michael L. Dustin,[1] Alex Gordon-Weeks,[4] Mark Coles,[1,*] Calliope A. Dendrou,[1,*] and Anita Milicic[2,5,*]

[1]Kennedy Institute of Rheumatology, University of Oxford, Roosevelt Drive, Oxford OX3 7FY, UK
[2]Jenner Institute, University of Oxford, Old Road Campus Research Building, Roosevelt Drive, Oxford OX3 7DQ, UK
[3]Vaccine Formulation Institute, Rue du Champ-Blanchod 4, 1228 Plan-Les-Ouates, Switzerland
[4]Nuffield Department of Surgical Sciences, University of Oxford, John Radcliffe Hospital, Oxford OX3 9DU, UK
[5]Lead contact
*Correspondence: mark.coles2@kennedy.ox.ac.uk (M.C.), calliope.dendrou@kennedy.ox.ac.uk (C.A.D.), anita.milicic@ndm.ox.ac.uk (A.M.)
https://doi.org/10.1016/j.celrep.2025.115938

SUMMARY

Immunological processes that underpin human immune responses to therapeutics and vaccine components, such as vaccine adjuvants, remain poorly defined due to a paucity of models that faithfully recapitulate immune activation in lymphoid tissues. We describe precision-cut human lymph node (LN) slices as a functioning, architecturally preserved, full-organ cross-sectional model system. Using single-cell transcriptomics and multiplexed imaging, we explore early inflammatory response to a potent, clinically relevant liposomal vaccine adjuvant containing a TLR4-agonist and QS-21 saponin. Both TLR4 and NLRP3 inflammasome activation are involved in the direct initiation of the inflammatory response to adjuvant by monocytes and macrophages (Mon./Mac.) with secretion of interleukin (IL)-1β, but not IL-18, dependent on TLR4 signaling. Innate lymphoid cells, including natural killer cells, are indirectly activated by Mon./Mac.-produced cytokines, signaling downstream to B cells via interferon-γ secretion. Resident LN stromal populations, primed both directly and indirectly by vaccine adjuvant, are instrumental in mediating inflammatory cell recruitment, particularly neutrophils.

INTRODUCTION

Much of our understanding of how the human immune system functions is extrapolated from studies in inbred mice. While enabling interrogation at both systemic and tissue-specific levels, animal models lack much of the complexity of humans and are often poorly predictive of responses to perturbations such as vaccination or infection.[1] Successful drug and vaccine design requires preclinical models that accurately reflect human immune responses to improve mechanistic understanding and accelerate clinical development of new preventive and therapeutic interventions.[2,3]

Initial modeling of human immune responses was restricted to *in vitro* 2D systems such as cell co-cultures or whole blood assays (WBAs),[4] which offer an over-simplified interpretation of immune interactions and fail to replicate the complex immune environment of lymphoid tissue where these responses initiate.[5] More recently, ultrasound-guided fine needle aspiration (FNA) of lymph nodes (LNs) have been used to monitor vaccine-induced responses[6–9] and autoantibody generation,[10,11] enabling temporal sampling of the immune system from within native tissue. However, in addition to complete loss of spatial information, FNAs predominantly capture T and B lymphocytes and miss out on rarer innate immune and non-hematopoietic stromal cells (NHSCs),[12] which are critical for induction and maintenance of immunity.[13,14]

LNs are highly organized and structured to enable cell-cell interactions required for an effective immune response. The importance of experimentally recapitulating these interactions to accurately model *in vivo* responses has long been appreciated,[15–17] and current efforts have turned to designing 3D *in vitro* replicas of human lymphoid tissue, either by combining single-cell suspensions with a matrix scaffold[2] or through reaggregation of dissociated tissue, as described with tonsil-derived organoids.[18] While 3D *in vitro* organoid models can be informative, the initial tissue disaggregation irretrievably leads to the loss of physiological architecture, and these models often lack critical tissue components such as the stromal compartment or extracellular matrix.[13] An alternative approach is *ex vivo* culture of non-dissociated tissue block explants, as reported with lymphoid tissues such as tonsil,[19,20] spleen[21,22] and, more recently, LN organs,[23] in studying responses to infection[24] and vaccination.[25] These explants retain the natural tissue architecture, but sample only a small

This is an open access article under the CC BY license (http://creativecommons.org/licenses/by/4.0/).

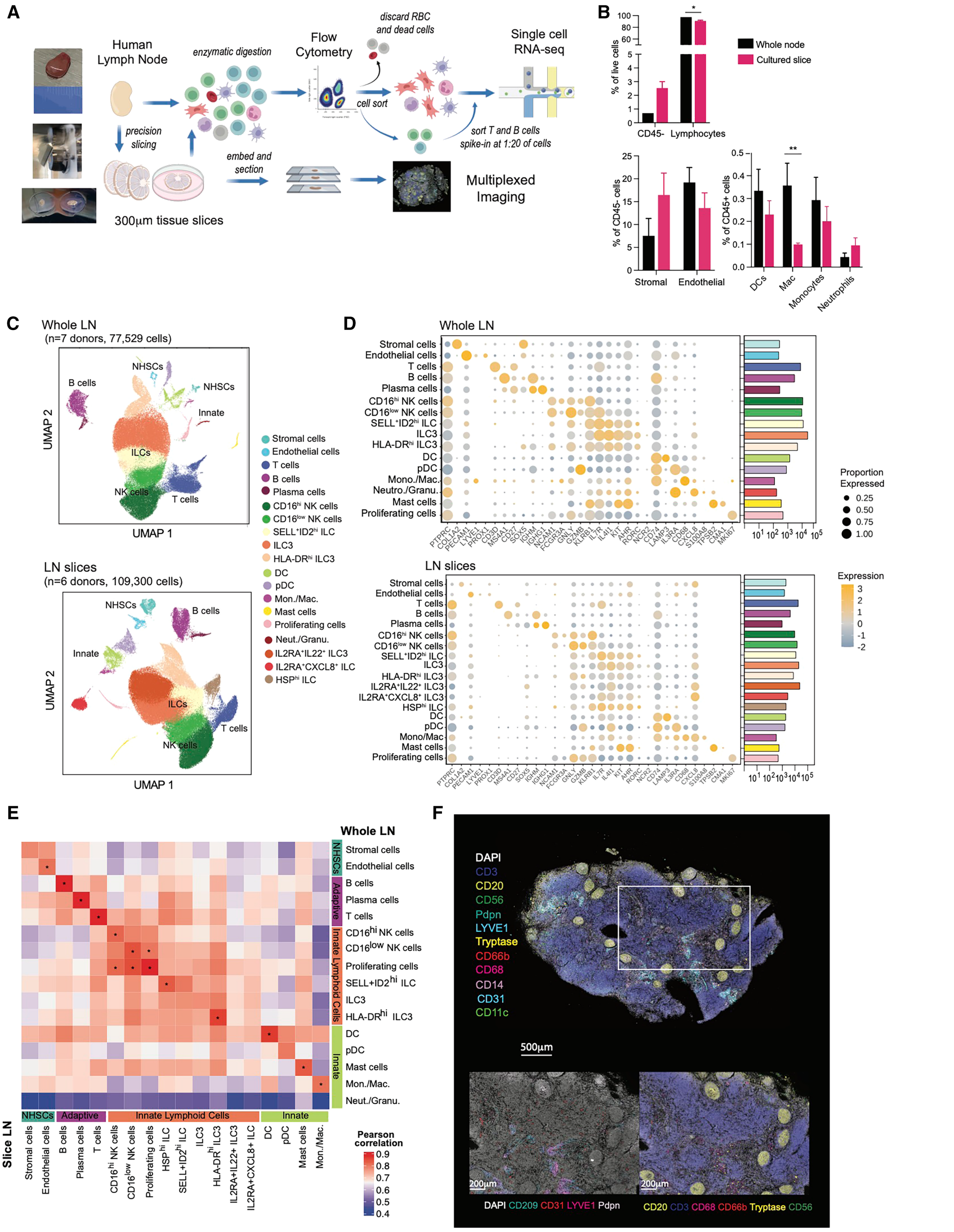

(legend on next page)

subsection of the whole organ, and thus may suffer from sampling bias and fail to capture important tissue structures, in addition to an imbalanced exposure to nutrients throughout the tissue chunk.[21] In contrast, precision cutting enables generation of systematic cross-sections of tissues such as liver, lung, spleen, or tumor,[22,26–28] and viable culture of lymphoid tissues such as tonsils[29] and adenoids[30] for study of T cell responses. In mice, LN slices have been shown to retain both acute function and the capacity for recall responses to prior vaccination.[31,32]

Here we establish a protocol for generation and culture of 300-μm-thick, full-organ cross-sections of human LNs, as an architecturally preserved and functionally responsive model system, enabling direct study of early innate immune responses across cell types, including the NHSC compartments that organize and orchestrate LN responses.[13] Using this approach, we sought to profile early immune events, particularly within the innate and stromal compartments, at the single-cell level during induction of inflammation by a potent, clinically relevant vaccine adjuvant. Adjuvants can induce an effective inflammatory innate immune response, enhancing adaptive immunity,[3] although complete understanding of their mechanism of action has been limited by poor translatability from animal models, with even licensed adjuvants lacking fully defined mechanisms.[3] Adjuvant AS01 by Glaxo Smith Kline (GSK) combines a TLR4 agonist with saponin QS-21 in a liposomal formulation and is included in the highly effective vaccines against shingles (Shingrix) and malaria (Mosquirix), yet our mechanistic insights of its mode of action derive mostly from mouse studies[33–36] or human blood.[33,37]

Most potent adjuvants are proprietary, which further restricts our knowledge of their immunostimulatory mechanisms. We employed an open-access adjuvant LMQ, similar in composition to AS01 and with proven safety and efficacy in animal models,[38,39] to *ex vivo* stimulate precision-cut tissue slices of live human LNs. From a single-cell transcriptomic map of the early adjuvant-induced events, we observed a pivotal function for innate lymphoid cells (ILCs), particularly natural killer (NK) cells, in translating inflammation into downstream adaptive immune responses that could lead to enhanced protection. By stimulating intact LN tissue, we reveal the key role of resident NHSC, a previously underappreciated cell type in studies of vaccine responses, in the amplification of the inflammatory response within the human LN.

RESULTS

Cell populations of the human LN are transcriptionally and architecturally preserved in precision-cut slices

To dissect functional responses of human secondary lymphoid tissue following immune perturbation, we developed an *ex vivo* culture system that allows individual cell populations to be observed in their physiological tissue microenvironment. Healthy non-inflamed human LNs were obtained through routine elective cholecystectomy, excised from surrounding connective tissue. A method was developed for precision cutting of whole LNs into 300-μm-thick, full-organ cross-sections that retained viability over short-term culture (STAR Methods, Figure S1), allowing assessment by a range of analytical assays (Figure 1A). A thickness of 300 μm was chosen as the thinnest reproducible cross-section, to maximize the number of slices obtained per LN while retaining tissue viability. Cell populations in a whole LN at baseline remained present and viable within slices (Figure 1B), although a 3.7-fold reduction in the percentage of macrophages (Mac.) was observed in LN slices after culture.

To verify that LN slices are representative of the cell populations and phenotypes in an intact LN, we compared their full transcriptomes: LNs were either dissociated into single-cell suspensions or cut into slices and cultured for 20 h in complete RPMI, prior to enzymatic digestion and single-cell RNA-sequencing (scRNA-seq) analysis. Multiple slices (average of four per condition) from each donor were pooled after culture to account for differences in the anatomical location of specific cell types. Red blood cells (RBCs) and dead cells were removed by cell sorting, and as T and B cells dominate numerically within the LN (Figure 1B), they were sorted separately and added (spiked) back at a 1:20 ratio to enable more balanced representation across all cell populations. Transcriptomic data from 77,529 "whole LN" cells from seven donors, and 109,300 "LN slice" cells from six donors, were separately integrated by donor, followed by unsupervised clustering analysis to identify cell populations across whole or sliced LNs (Figure 1C). Cell clusters in both whole and sliced LNs were annotated using expression levels of canonical gene markers and gene sets (Figure 1D), identifying clusters corresponding to myeloid, stromal, and lymphoid populations, including the spiked-in T and B lymphocytes. After T/B cell depletion, most cells within both whole and sliced LNs were innate lymphoid cells (ILCs). This includes NK cells, with two clusters identified based on

Figure 1. Precision-cut human LN slices are transcriptionally and architecturally preserved in culture, reflecting a whole native human LN *ex vivo*

(A) Schematic of workflow for analysis of whole or sliced human LNs.

(B) Live cell-type distribution from either whole digested human LNs ($n = 3$) or from 300-μm LN slices after 20 h in culture ($n = 3$) by flow cytometry. Data are mean and SEM. $^{*}p < 0.05$, $^{**}p < 0.005$ by two-way ANOVA with Sidak's multiple comparisons test; all other comparisons were not significant.

(C) UMAP plots of whole human LNs from 77,529 cells from seven donor LNs, and human LN slices of 109,300 cells from six individual donors, pooled from multiple slices per donor, analyzed by scRNA-seq, colored by cluster and cell type.

(D) Dot plots showing expression of marker genes for each cell cluster across whole (top) and slice (bottom) LN datasets. Color indicates relative log-normalized level of expression across clusters and dot size the proportion of each cluster expressing each gene. Bar plots indicate the absolute cell counts for each cell type.

(E) Comparison of gene expression across all cell types between whole LNs (vertical) and slice LNs (horizontal): heatmap of the Pearson correlation coefficient between matched clusters for the median gene expression of the top 2,000 most variable genes with * indicating where $r > 0.8$.

(F) Multiplex stained image of a human LN slice section after 20 h in culture spatially identifying cell types with the indicated markers. Lower images show greater magnification of the boxed area, with markers for stromal (left) and leukocyte (right) cell types. Scale bars are indicated, and image is representative of two individuals.

See also Figure S1 and S2.

CD16 transcriptional expression and confirmed at the protein level by flow cytometry and conventional gating strategies[40] (Figure S2). In the whole LN, ILCs formed three clusters, all expressing *KIT* (CD117), *IL23R,* and *RORC*, associated with an ILC3 phenotype. ILC3s were also the major subset detected at the protein level, with most being NKp44^{-} (Figure S2). While an NKp44^{+} population was detected at the protein level, only low levels of transcript (*NCR2)* were found, consistent with previously described discrepancy between NKp44 transcript and protein expression.[41] Notably, however, *NCR2* transcript levels were similar to those detected in NK cells and were restricted to the ILC3 cluster. Further *NCR* transcripts, *NCR1* (NKp46) and *NCR3* (NKp30), were found at the highest levels within the IL2RA^{+}SELL^{+}ID2^{hi} ILC cluster, which also expressed *GATA3*, and may represent a more naive ILC subset.[41] Conventional flow cytometry gating strategies identified ILC1 and ILC2 (Figure S2), but these populations were rare and variable between donors, and did not form a separate cluster by scRNA-seq analysis. Three additional ILC clusters were apparent in LN slices by transcriptional analysis: IL2RA^{+}IL22^{+} ILC3, HSP^{hi} ILCs, and IL2RA^{+}CXCL8^{+} ILCs. This suggests transcriptional differentiation of ILCs into further phenotypes, as might be expected due to the plasticity of this cell type and its sensitivity to environmental cues, such as those introduced by slicing[42–44] (Figures 1C and 1D). Indeed, the proportion of ILC3 (defined by scRNA-seq) was significantly decreased in LN slices as further transcriptional clusters appeared (Figure S1), although the distribution of ILC subsets by flow cytometry and conventional gating strategies was similar between whole and sliced LNs after culture (Figure S2). A cluster of neutrophils/granulocytes was detectable in the whole LN, and by flow cytometry after culture and sorting (Figure S1), but absent from LN slice transcriptomic data, reflecting the difficulty of capturing this cell type for transcriptomic analysis after culture.[45,46] Cell abundances of matched cell populations beyond ILCs were not significantly different between whole and sliced LN datasets, including myeloid cells (dendritic cells [DCs], monocytes/macrophages [Mon./Mac.], mast cells), plasmacytoid DCs, and stromal and endothelial cells (Figure S1).

To compare the cell type/states between whole and sliced LNs, the median gene expression of the top-most variable features across datasets was computed for each cluster, and the Pearson correlation coefficient calculated between clusters in whole and sliced LN datasets. This confirmed a strong correlation in the gene-expression profiles of sliced LN cell clusters with matched cell clusters in the whole LN, demonstrating the preservation of cell phenotype *ex vivo* (Figure 1E).

The LN is a highly organized structure and to faithfully recapitulate its immune pathways individual cells need to be maintained within their natural microenvironment. Multiplexed imaging of slices confirmed the preservation of LN architecture in culture, including B cell (CD20) follicles, T cell (CD3) paracortical regions, lymphatic sinuses including of the medulla (CD209, LYVE1), and blood vessels (CD31) (Figure 1F).

Human LN slices respond to inflammatory perturbation *ex vivo*

Having established overall preservation of tissue architecture and cell composition in LN slices, we sought to profile responses to vaccine adjuvant as a known driver of transient inflammation in effective immunization.[4,38,39] In particular, high potency has been demonstrated for adjuvants that contain multiple immunostimulatory components, such as AS01.[33,36,47,48] We employed an AS01-like liposome (L)-based adjuvant, containing a synthetic toll-like receptor 4 (TLR4) agonist 3D-6-acyl-PHAD (3D6AP) (M), and QS-21 saponin (Q)—abbreviated as LMQ—to study the inflammatory immune response within human LN tissue slices (Figure 2A).

To compare adjuvant uptake of *ex vivo* LN slices with LN *in vivo*, we injected mice intramuscularly (i.m.) with fluorescently labeled LMQ and analyzed cell-type specificity of adjuvant uptake in the draining lymph node (dLN). Equivalent cell populations were positive for the adjuvant at two time points in both LN slices cultured with LMQ and in dLNs following i.m. LMQ injection (Figure 2B), verifying that adjuvant uptake in slices mirrors cell-type specificity *in vivo*. Lymphocytes demonstrated the lowest adjuvant uptake capacity, and most DCs and neutrophils were positive for fluorescent LMQ at later time points. No significant difference was observed within cell types between *ex vivo* and *in vivo*, although there was a trend toward lower uptake in monocytes at the earliest time point, and higher uptake by macrophages at the later time point. As the proportion of adjuvant-positive cells increased over time, responses were subsequently assayed at 20 h post-adjuvant stimulation, with the aim of capturing the highest functional impact on each cell type. Transcriptional responses at 20 h were also found to correlate with adjuvant concentration in culture (Figure S1).

Cell populations in human LN slices were also capable of selectively taking up fluorescently labeled adjuvant (Figure 2C). In line with results from mouse LNs, there was a hierarchy between cell types, with the highest uptake among leukocytes seen with myeloid cells, including dendritic cells (DCs), macrophages and monocytes, while lymphocytes and neutrophils displayed little adjuvant uptake at 20 h of culture.

Adjuvant stimulation resulted in proinflammatory cytokine production, with a range of cytokines detected across donors in the culture supernatant (Figure 2D; Table S2). Assessment of serial LN slices within individual donors, pooled by treatment across multiple slices, revealed significant induction of tumor necrosis factor (TNF)-α and interleukin (IL)-6 by LMQ (Figure 2E), recapitulating findings from the serum and injection site in mice after immunization with LMQ.[38] Crucially, the LN slice model captures the diversity of human responses in the type and levels of cytokines produced, while enabling paired analyses against the baseline control. Together, this demonstrates that cellular uptake of adjuvant within the slices triggers pathways and functional responses that recapitulate LMQ-induced mediators observed *in vivo,*[38] supporting LN slice culture as a 3D human model for *ex vivo* study of adjuvant-induced inflammation.

LMQ adjuvant directly activates TLR4 and the NLRP3 inflammasome in tissue monocytes/macrophages

To identify which cells are most likely to initiate inflammation by responding directly to the TLR4-agonist in LMQ, we examined whole LN cell populations for the expression of TLR4, its co-receptors, CD14 and LY96, and the associated signaling molecules (Figure 3A). There was little to no expression of these molecules in lymphoid subsets, including innate lymphocytes such

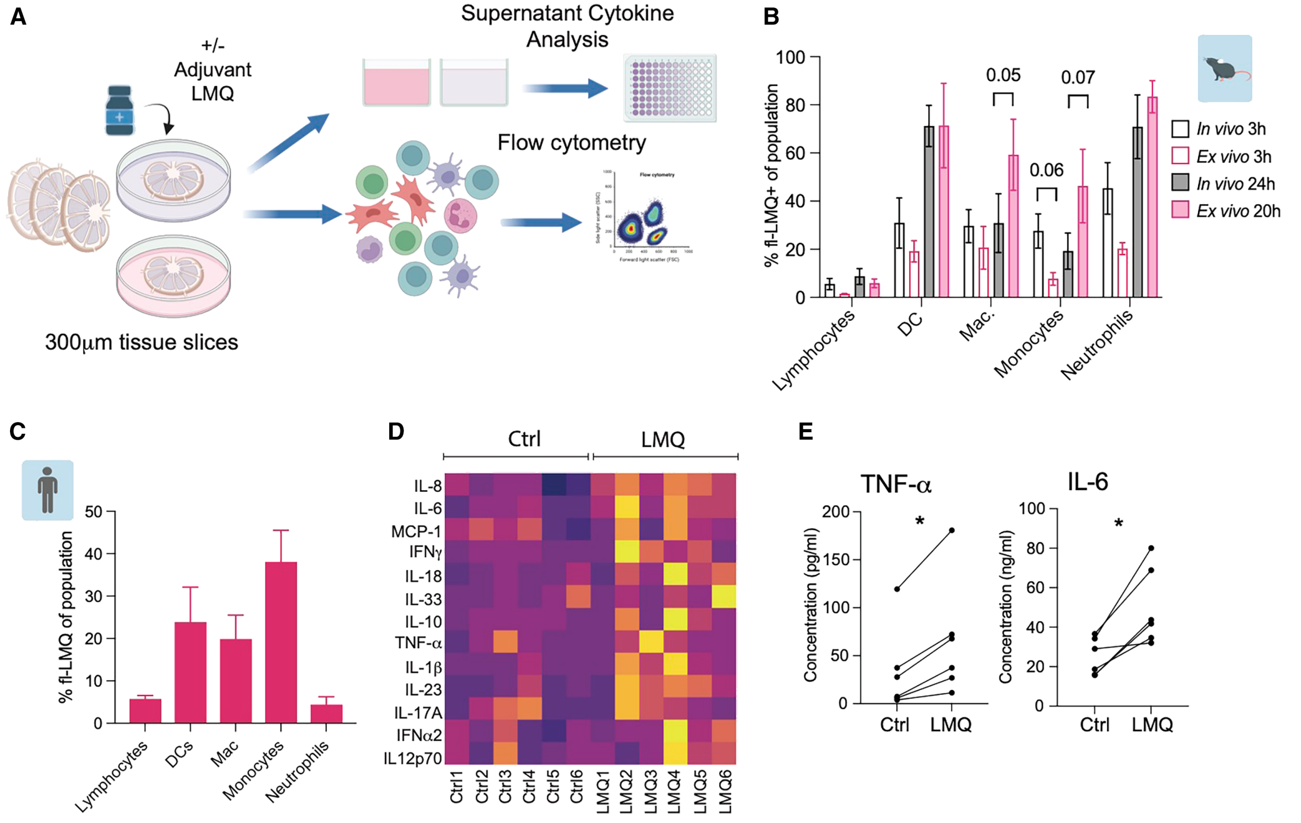

Figure 2. Human LN slices respond to inflammatory perturbation *ex vivo*

(A) Schematic of workflow for analysis of sliced human LNs.

(B) Kinetics and cell-type specificity of uptake of a fluorescently labeled liposomal adjuvant LMQ in the mouse dLN following intramuscular (i.m.) injection (*in vivo*; $n = 4$) or in cultured LN slices (*ex vivo*; $n = 3$, one slice per mouse). Data are mean and SEM. All *in vivo* vs. *ex vivo* comparisons were non-significant by multiple unpaired t tests with Welch correction at each time point within cell type; p values < 0.1 are indicated.

(C) Proportion of adjuvant-positive cell populations within human LN slices after 20 h of *ex vivo* stimulation ($n = 3$). Data are mean and standard deviation (SD).

(D) Cytokine concentration in slice culture supernatants after 20 h without (Ctrl) or with LMQ adjuvant (LMQ). Heatmap indicates average concentration, averaged from two to four slices per donor and condition, with slices successively assigned to either Ctrl or LMQ condition, scaled by cytokine (row) ($n = 6$ donors).

(E) TNF-α and IL-6 concentration in supernatants from paired donor conditions without (Ctrl) or with LMQ adjuvant (LMQ) after 20 h. Each point is the average of two to four slices, $^{*}p < 0.05$ by Wilcoxon matched-pairs signed rank test ($n = 6$ donors).

See also Table S2.

as NK cells. Myeloid lineages showed the highest expression, in particular monocytes/macrophages (Mon./Mac.), which expressed transcripts for TLR4 and both co-receptors. Expression patterns of *TLR4*, *CD14,* and *LY96* were similar in the LN slices after stimulation in culture (Figure S3).

The impact of TLR4 signaling was assessed by pairwise differential gene-expression analysis within donors, identifying differentially expressed genes (DEGs) between unstimulated and LMQ-stimulated cell clusters (Table S3). Few upregulated DEGs were detected in DCs (Figure S3). In contrast, in Mon./Mac. LMQ stimulation resulted in a strong transcriptional response, including upregulation of activation markers (*IDO1*, *CCR7*, *CD83*, and *CD40*) (Figure 3B). Gene set enrichment analysis (GSEA) of significant DEG identified TLR signaling among the top enriched pathways in response to LMQ (Figure 3C; Table S4), confirming direct cell activation by the TLR4 agonist. Gene sets related to interleukin signaling were also enriched, suggesting a positive post-activation feedback loop via expression of cytokine mediators.

In mice, LMQ drives inflammation through synergistic TLR4 and QS-21-mediated NLRP3 activation, inducing release of IL-1β and IL-18.[34,38,49] We therefore examined the expression of NLRP3 components across LN cell populations. Gene signatures for the canonical inflammasome complex, scored by UCell,[50] were significantly increased in response to LMQ within Mon./Mac. (Figure 3D), which demonstrated the highest median expression across cell types. Interestingly, the inflammasome gene signature was also significantly upregulated in B cells, but to a much lower level. Initiation of the inflammasome pathway results in Caspase 1 activation to cleave pro-interleukin 1 and pro-interleukin 18 into their active forms for release, with transcription induced by TLR-mediated priming.[51,52] Both IL-1β and IL-18 cytokines were significantly higher in slice culture supernatants after LMQ treatment (Figure 3E).

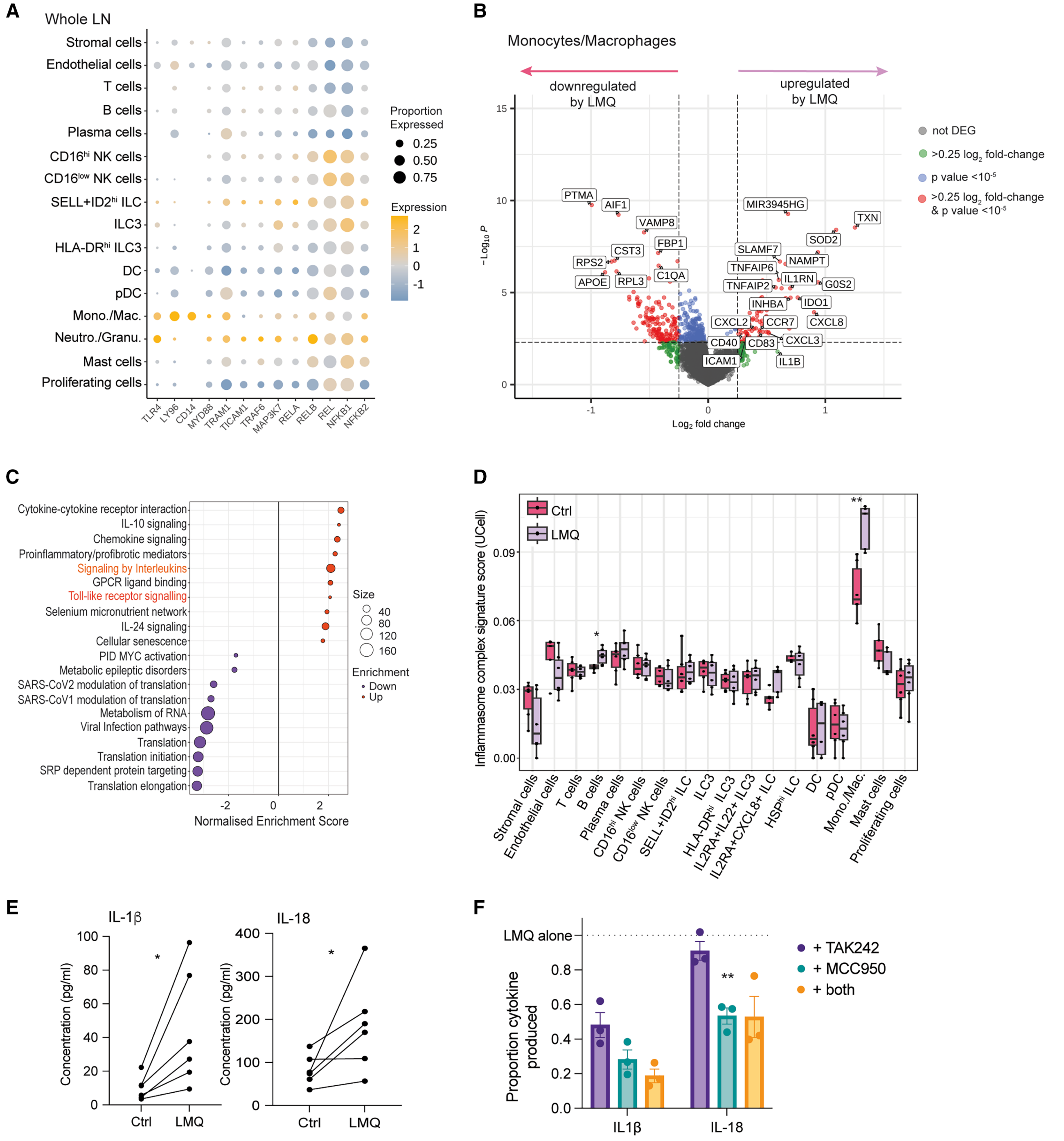

Figure 3. Adjuvant directly activates TLR4 and the NLRP3 inflammasome in tissue monocytes/macrophages

(A) Dot plot showing expression of TLR4 signaling components for each cell cluster within whole human LNs. Color indicates relative log-normalized level of expression across clusters and dot size the proportion of each cluster expressing each gene.

(B) Volcano plot indicating DEG between LMQ-stimulated (right: upregulated) and control (left: upregulated) conditions in monocytes/macrophages. Genes with $\log_2$ fold-change >0.25 are indicated in green, those with p value < 10^{-5} in blue and those fulfilling both criteria in red.

(C) Enriched canonical pathways among significantly ($p < 0.05$) DEGs, which are up- (orange) and down- (purple) regulated in monocytes/macrophages by adjuvant (LMQ) stimulation.

(D) Gene signature scoring (UCell) of the Canonical Inflammasome Complex Gene Ontology gene set according to cell type and sample type (magenta = control, lavender = LMQ-treated). The median UCell scores for each individual donor are indicated. **$p < 0.05$ and *$p < 0.5$ by two-way ANOVA with Wilcox test and Benjamini Hochberg correction.

(legend continued on next page)

To evaluate relative contributions of TLR4 signaling and NLRP3 activation in LMQ-mediated inflammation, LN slices were pre-treated with molecular inhibitors of these two pathways, TAK242[53] and MCC950,[54] respectively. MCC950-mediated NLRP3 inhibition reduced the release of both IL-1β and IL-18, while inhibition of TLR4 signaling by TAK242 decreased production of IL-1β but not IL-18 (Figure 3F). This demonstrates that IL-18 is less reliant on TLR4-mediated priming of transcription than IL-1β, consistent with a constitutive expression of IL-18 precursor,[55] and further indicated by lack of upregulated *IL18* transcription despite increased protein release (Figure S3). Therefore, LMQ initiates inflammation through direct activation of Mon./Mac. within the human LN, resulting in the production of inflammatory cytokines, including IL-1β. This aligns with results with AS01 in mice showing that inflammasome activation drives IL-1β production in bone-marrow-derived macrophages and that QS-21 mediated activation of LN macrophages, particularly within the subcapsular sinus.[33,34]

Indirect activation of ILCs by vaccine adjuvant leads to downstream signaling to B cells in spatially preserved LN tissue

Given the preserved tissue architecture in LN slices, we investigated the possibility of indirect, cytokine-mediated, activation of cell types that lack the ability to directly respond to TLR4 signaling. After depleting T and B cells, ILCs were the major population within the human LN (Figure 1C), with a significant increase in the proportion of $IL2RA^{+}IL22^{+}$ ILC3s in LMQ-treated slices (Figure 4A). *IL22* transcripts were also significantly upregulated within the $IL2RA^{+}IL22^{+}$ ILC3 cluster following LMQ stimulation (Figure 4B) and IL-22 cytokine increased in the supernatants of three out of four donors tested (Figure 4C). We observed strong correlation (r^2 = 0.8974) between IL-22 concentration and the proportion of $IL2RA^{+}IL22^{+}$ ILC3s in the same donor slices (Figure 4D). This provides further evidence that released IL-22 originates from activated ILC3, demonstrating the ability of LN slices to retain donor-to-donor variation across multiple readouts. ILC3s can be activated by a combination of IL-1β and IL-23 to release IL-17A, or more particularly IL-22.[42,43,56] While IL-17A was not induced at either the transcriptional or protein level (Figure S4), both IL-1β (Figure 3E) and IL-23 (Figure S4) were significantly increased in LMQ-stimulated supernatants, and are likely responsible for IL-22 production by ILC3s. To the best of our knowledge, IL-22 release by ILC3 in response to this adjuvant composition has not been previously described in mice, although it has been observed in response to a viral-vectored vaccine.[57] Interestingly Th17-related cytokines are enriched in the dLN of mice within the first hours following AS01 injection.[33]

In addition to IL-1β, LMQ also induced IL-18 release (Figure 3E), which in combination with other innate cytokines such as IL-12, activates NK cells to produce IFNγ.[58,59] One of the most clearly induced genes overall was *IFNG* within the $CD16^{low}$ NK cell subset (Figure 4E), consistent with results in mice after vaccination with AS01-adjuvanted vaccines, with NK cells being the major producer of interferon (IFN)-γ.[33] IL-12 was not detected in culture supernatants (Figure S4), implying that IL-18 synergizes with an alternate innate cytokine to induce *IFNG* transcription in NK cells in humans. In mice IL-12p40 blockade significantly reduces IFN-γ production in NK cells in response to adjuvanted vaccine,[33] although the p40 subunit is also a component of IL-23, which has also been described to synergize with IL-18 to induce IFN-γ production in NK cells.[60] IL-23 may therefore provide a synergistic signal in the human LN, as it was upregulated within LMQ-activated human LN slice supernatants (Figure S4). Upregulated transcription was accompanied by elevated IFN-γ in LMQ-stimulated slice culture supernatants (Figure 4F). *IFNG* transcription was not significantly enhanced in T cells, which generally showed little transcriptional change in response to LMQ (Figure S4), consistent with the small percentage of $CD8^{+}$ T cells producing IFN-γ in the dLN of AS01-immunized mice.[33] The contribution from other ILC subsets was also low (Figure S2; Table S3), although ILC1s—including recruited migratory subsets—have been shown in mice to respond to AS01-adjuvanted vaccine with IFN-γ production.[61] Together, these results indicate NK cells as the primary source of IFN-γ in human LN responses to LMQ. Release of IFN-γ was reduced by pre-treatment of slices with both TLR4 and NLRP3 inhibitors (Figure 4G), despite the lack of TLR4 receptors or inflammasome upregulation in NK cells (Figures 3A and 3D), further supporting their indirect activation by LMQ. Interestingly, transcriptional profiling implicated B cells as a potential target of NK cell-derived IFN-γ, demonstrating significant upregulation of *IRF1* and *STAT1* (Figure 4I), and enrichment in interferon signaling pathways in response to LMQ (Figure 4J; Table S4). As IFN-α was not upregulated in culture supernatants (Figure S4), interferon signaling in B lymphocytes is predicted to be in response to IFN-γ produced by NK cells.

IFN-γ has been shown to operate within only a 30-μm radius in dense cell environments.[62] We therefore explored the spatial relationship between NK cells and B cells, using multiplexed imaging, to determine the potential for proximal signaling. While few individual $CD3^{-}CD56^{+}$ NK cells were identified within B cell-rich follicles, clusters of NK cells could be observed in extrafollicular regions populated by macrophages (Figures 4H and S4), indicating them as the likely source of NK cell-activating cytokines. Interestingly, these NK cells also appeared proximal to clusters of B cells, with "nearest neighbor" analysis indicating that ∼40% of NK cells were within a 30-μm radius of B cells

(E) Concentrations of IL-1β and IL-18 detected in slice culture supernatants after 20 h without (Ctrl) or with LMQ adjuvant (LMQ) from six donors, where each point is the average of three to four slices, *$p < 0.05$ by Wilcoxon matched-pairs signed rank test.
(F) Relative concentration of IL-1β and IL-18 in slice culture supernatants following 20 h stimulation with adjuvant LMQ in the absence or presence of inhibitors TAK242 (TLR4 inhibitor), MCC950 (NLRP3 inhibitor), or a combination of both. Proportion of each secreted cytokine was calculated relative to LMQ stimulation for each donor (indicated by dotted line at 1.0). Data are mean and SEM (n = 3, each point is the average of three to four slices from one donor). **$p < 0.01$ by mixed-effects analysis with Dunnett's multiple comparisons test to LMQ alone.
See also Figure S3 and Tables S3 and S4.

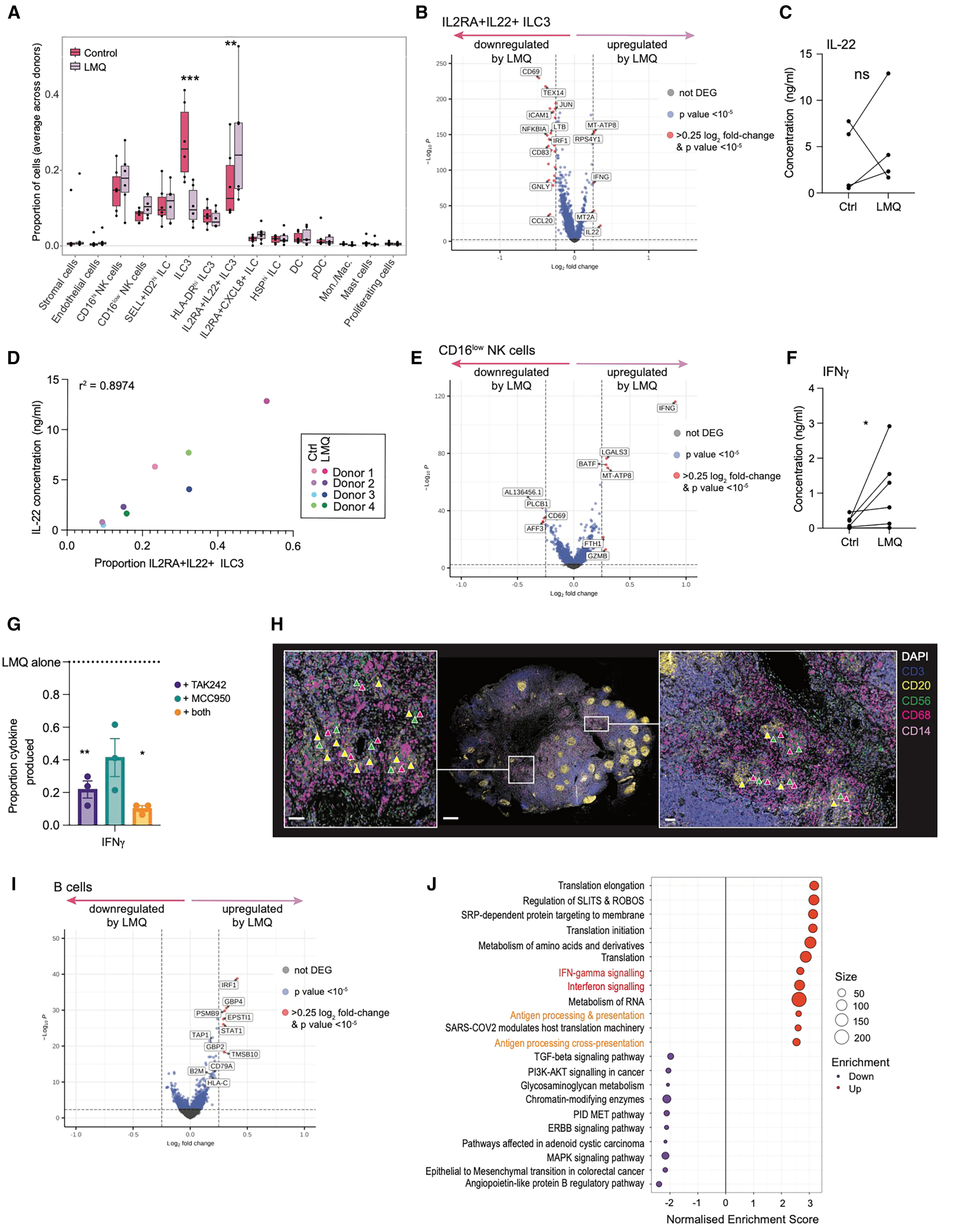

(legend on next page)

outside follicles. Stratification according to cluster size also indicated that larger clusters of NK cells tended to localize with denser regions of B cells outside follicles, over short (~30 μm) distances (Figure S4).

In addition to enrichment in IFN-γ signaling pathways, B cells also showed upregulated expression of gene sets associated with protein translation, and antigen processing and presentation (Figure 4J), which have also been associated with higher antibody responses to vaccination.[63] Taken together, this reveals a potential mechanism by which the adjuvant signals indirectly to the adaptive arm of the immune system, through direct stimulation of myeloid populations and via NK cell-produced IFN-γ, ultimately enhancing humoral immunity.

NHSC populations are primed both directly and indirectly by vaccine adjuvant to mediate inflammatory cell recruitment in the LN

The effect of vaccine adjuvants on LN-resident NHSC populations is rarely studied and generally underappreciated, with AS01 responses in mice described to rely on TLR4 signaling in hematopoietic rather than stromal cells.[64] Stromal cells are critical in shaping the immune milieu within the LN,[13] and are retained within LN slices (Figures 1F and S5), providing a unique opportunity to study their contribution to adjuvant-induced inflammation. Both mesenchymal stromal and endothelial cells (ECs) demonstrated uptake of fluorescently labeled adjuvant, both in slices (Figure 5A) and *in vivo* in mouse dLN following i.m. injection (Figure S5). LMQ-induced gene-expression changes in NHSC populations revealed a strong transcriptional response in ECs (Figure 5B), including gene set enrichment of the TLR signaling pathway: *JUN*, *TAB2*, and *MAPK8* (Figure 5C; Table S4), suggesting that LMQ signals directly to ECs and induces transcriptional activation through TLR4 stimulation. The IL-1 signaling pathway was also enriched, indicating a potential role for IL-1β in a further indirect activation of ECs. Mesenchymal stromal cells also exhibited a strong transcriptional response to LMQ (Figure 5D) but with enrichment of cytokine-mediated, rather than TLR, signaling pathways (Figure 5E; Table S4), suggesting that the stromal response to LMQ may mainly be due to indirect signaling.

Clear upregulation in the transcription of cytokines associated with neutrophil chemotaxis, such as *CXCL1*, *CXLC2,* and *CXCL8*, was observed in both NHSC subsets in response to LMQ (Figures 5B and 5D), as was upregulation of *IL33* and *TSLP* by mesenchymal stromal cells (Figure 5D). CXCL8 was also significantly increased in culture supernatants (Figure 5F), with further contribution from ILCs and Mon./Mac. (Figure S5), while CXCL2 production was restricted to stromal cells and ECs. As mice lack a direct CXCL8 orthologue, the CXCL8 production detected here is unique to human LNs, while production of CXCL1, a functional orthologue, has been reported in mice post vaccination with LMQ[38] and AS01.[34,36] Indeed, neutrophils were rapidly recruited to the dLN after i.m. injection of LMQ in mice, while monocytes and DCs accumulated more steadily over 16 h (Figure S5). Inferred cell-cell communication, by CellPhoneDB analysis,[65] indicated significant crosstalk in response to LMQ between both stromal cells and ECs and innate cells, across multiple cytokine/chemokine-receptor interactions, including *CXCL2*, *IL33*, *TSLP,* and *TNFSF10* (TRAIL) (Figure S5; Table S5). Together, this reveals how adjuvant primes stromal cells and ECs to recruit and support innate cell mediators of inflammation for downstream amplification of the immune stimulus and illustrates their key role as orchestrators of the response to vaccination in human lymphoid tissue.

To explore which specific populations of LN NHSCs, of which multiple subsets have been described,[66,67] were responsible for production of neutrophil chemotactic factors, we performed further subclustering on the EC and mesenchymal stromal cell populations. While cell numbers were overall limited, we identified clusters likely containing populations of fibroblast reticular cells (FRCs), follicular dendritic cells (FDCs) and pericytes, and both blood (BECs) and lymphatic (LECs) endothelial cells (in addition to contaminating leukocytes) based on expression of key cell-specific marker genes[66] (Figure 5G). This also revealed TLR4 and TLR4 co-receptor expression within a fraction of the LEC- and FDC-containing clusters, potentially reflecting the

Figure 4. Indirect activation of ILCs by adjuvant results in downstream signaling to B cells in spatially preserved LN tissue

(A) Proportion of each cell type in unstimulated control (magenta) and LMQ-stimulated (lavender) LN slices, each point represents an individual donor. $^{**}p < 0.005$, $^{***}p < 0.0005$, ANOVA using a linear model.

(B) DEGs in IL2RA + IL22+ ILC3 between LMQ-stimulated (right: upregulated) and control (left: upregulated) conditions. Genes with $\log_2$ fold-change >0.25 and p value $< 10^{-5}$ are indicated in red.

(C) IL-22 concentration in slice culture supernatants after 20 h culture without (Ctrl) or with LMQ adjuvant (LMQ) from paired donor conditions ($n = 4$, each point is the average of two to four slices from one donor), $^{*}p < 0.05$ by Wilcoxon matched-pairs signed rank test.

(D) Proportion of $IL2RA^{+}IL22^{+}$ ILC3 cells per donor and per condition against IL-22 concentration detected in paired culture supernatants. r^2 value was calculated by simple linear regression.

(E) DEGs in $CD16^{low}$ NK cells as in (B).

(F) IFN-γ concentration in slice culture supernatants as in (C).

(G) Relative IFN-γ concentration in slice culture supernatants following 20 h stimulation with adjuvant LMQ in the absence or presence of inhibitors TAK242 (TLR4 inhibitor), MCC950 (NLRP3 inhibitor), or a combination of both. Proportion of each secreted cytokine was calculated relative to LMQ stimulation for each donor (indicated by dotted line at 1.0) ($n = 3$, each point is the average of two to four slices from one donor). Data are mean and SEM. $^{**}p < 0.01$, $^{*}p < 0.05$ by mixed-effects analysis with Dunnett's multiple comparisons test to LMQ alone.

(H) Multiplex stained image of whole human LN for NK cells ($CD3^{-}CD56^{+}$, green triangles), macrophages (CD68, magenta triangles), and B cells (CD20, yellow triangles). Middle image scale bar indicates 400 μm. Inset images are zoomed in regions of boxed areas, where scale bars are 50 μm. Images are representative of three individuals.

(I) DEGs in B cells as in (B).

(J) Enriched canonical pathways among significant ($p < 0.05$) DEGs which are up- (orange) and down- (purple) regulated in B cells in response to LMQ stimulation. See also Figure S4 and Tables S3 and S4.

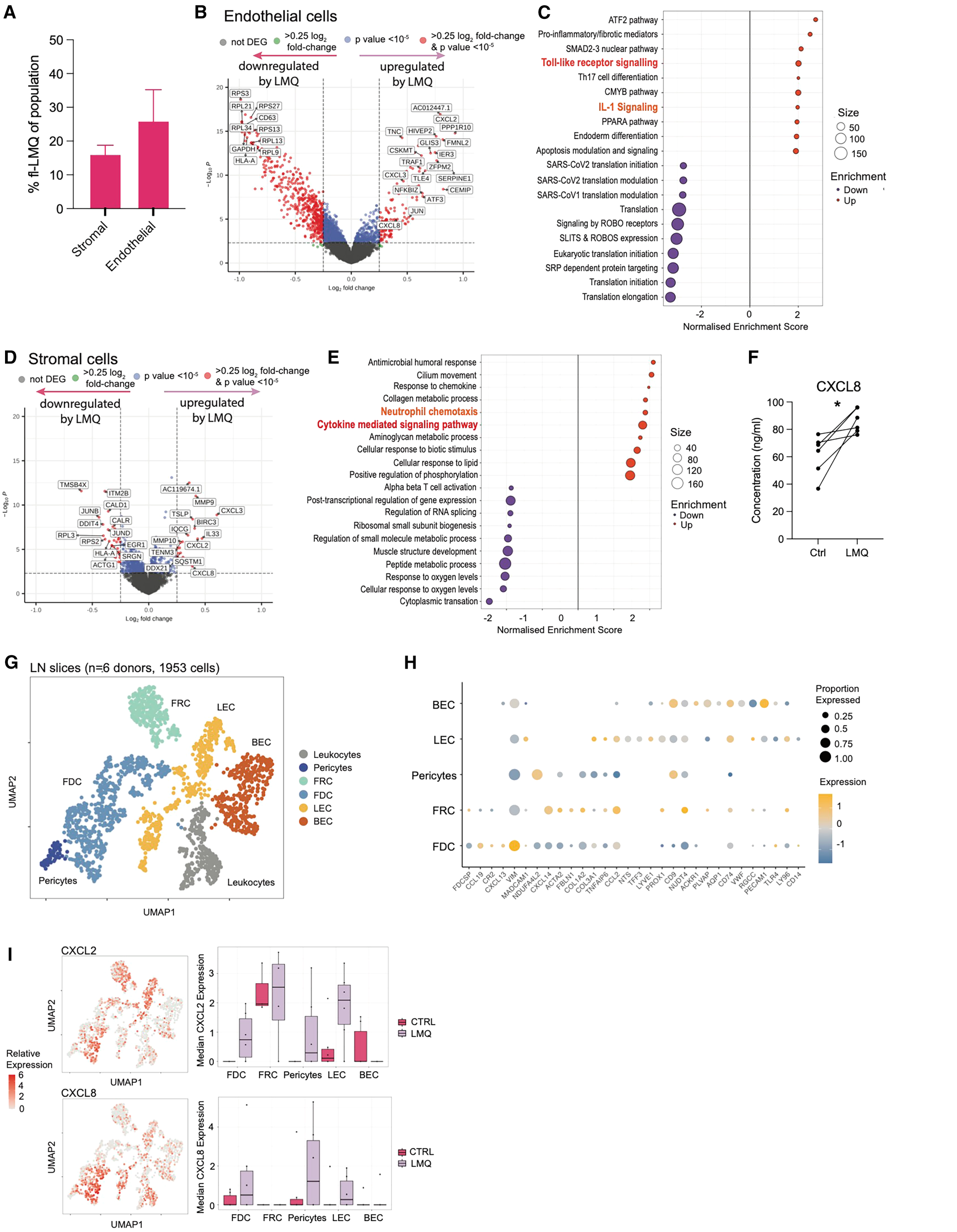

(legend on next page)

true FDC population, and as previously described in humans.[68,69] Expression of TLR4 tracked with expression of *CXCL2* and *CXCL8* enriched within these subsets (Figures 5H and 5I), and with transcription increased upon LMQ treatment (Figure 5I). Interestingly, some increased transcription was also observed within the pericyte cluster. Collectively, our data indicate a key role for LN NHSC compartments in the human response to LMQ adjuvant, and in particular in production of chemotactic signals that amplify the inflammatory state through recruitment of innate cell types such as neutrophils.

DISCUSSION

Rational design of immune modulators and vaccines relies on a thorough understanding of both their mechanism of action and the physiological immune processes they aim to perturb. To date, much of this knowledge comes from animal studies, and due to the relative lack of translation to clinical efficacy, the advantages of studying new therapeutics in human tissue early in the development pipeline are beginning to be appreciated.[2,3] Evolutionary divergence means that certain immune pathways are not present in rodent animal models, and thus their contribution cannot be ascertained.[1] In parallel, genetic and environmental divergence in people results in a huge variation in responses[63,70] that cannot be captured by genetically and environmentally controlled laboratory animals. Here we present a human *ex vivo* LN slice model in which the response to an inflammatory stimulus can be directly studied, with a variety of readouts, to reveal mechanistic insights into the triggered processes while retaining the donor-to-donor variation inherent to humans.

To study native human LN responses while preserving the tissue microanatomy, we developed precision cutting and subsequent culture of human LN slices. Precision cutting has been successfully applied in other organs[21,22,26,27,29] and species,[31] and is here extended to human LNs as the relevant tissue for assessment of vaccine responses. Small LN size allows for obtaining full-organ cross-sectional tissue slices and, by depletion of T/B lymphocytes, we were able to characterize rarer LN populations and their responses, including a previously underappreciated role for ILCs and NHSCs, in the adjuvant-driven inflammatory response.

Employing LMQ—a liposomal vaccine adjuvant containing a TLR4 agonist and saponin, on the path to clinical deployment[38,39]—we studied early induction of inflammation in human LN tissue. In mice, following i.m. injection, LMQ activates the NLRP3 inflammasome and induces proinflammatory cytokines in the dLN.[38] Our LN slice model, when stimulated with LMQ *ex vivo*, demonstrated cell-type specificity in adjuvant uptake equivalent to the mouse dLN, along with inflammasome-dependent secretion of proinflammatory cytokines IL-1β and IL-18. These results align with NLRP3-inflammasome activating properties of the QS-21 saponin component,[34,49] and with NLRP3-dependent IL-1β induction by LMQ in both mouse bone-marrow-derived macrophages and monocyte-derived macrophages from human peripheral blood.[38] Through the application of specific small molecule inhibitors TAK242 and MCC950 in the slice cultures, we further demonstrate that both TLR4 and inflammasome activation are required for IL-1β production in human LN tissue, but that IL-18 production was not dependent on TLR4-priming.

This adjuvant composition is associated with induction of Th1-type immunity through IFN-γ-mediated cytokine production,[33,38] with IFN-γ signaling required for T cell functionality in mice.[33] Correspondingly, in human LN slices, LMQ triggered significant production of IFN-γ by NK cells, following direct activation of Mon./Mac. and production of IL-18 leading to NK cell activation.[33,34] Responses to interferon signaling were detected in B cells, which were found in close proximity to both NK cells, shown to produce IFN-γ, and the Mon./Mac. capable of activating them. This sequential cell-cell communication was identified through use of architecturally preserved LN tissue. Direct inflammasome activation of B cells by LMQ may also play a part, as they displayed some NLRP3 component upregulation, although at much lower levels than in Mon./Mac. B cell transcriptomes were also enriched for antigen processing and presentation pathways, important in obtaining T cell help and enhancing antibody responses.[63,71] IFN-γ is upregulated in the serum of people immunized with AS01-adjuvanted vaccines,[33] with IFN-γ signaling associated with improved adaptive responses,[47] and antibodies considered critical for mediating protection.[48,72]

Unlike results described with AS01 in mice, neither activation of DCs nor T cells was observed in this human system.[23,34,36]

Figure 5. LN non-hematopoietic stromal cells (NHSCs) are primed both directly and indirectly by vaccine adjuvant to mediate inflammatory cell recruitment

(A) Proportion of adjuvant-positive cell populations within CD45$^-$ populations of human LN slices after 20 h of culture (n = 3). Stromal cells were defined as CD45$^-$CD31$^-$Pdpn$^+$ and endothelial cells as CD45$^-$CD31$^+$. Data are mean and SD.

(B) DEGs in ECs between LMQ-stimulated (right: upregulated) and control (left: upregulated) LN slices. Genes with $\log_2$ fold-change >0.25 are indicated in green, those with p value < 10^{-5} in blue, and those fulfilling both criteria in red.

(C) Enriched canonical gene sets among significant (p < 0.05) DEGs that are up- (orange) and down- (purple) regulated in ECs following adjuvant (LMQ) stimulation.

(D) DEGs in stromal cells as in (C).

(E) Enriched gene ontology biological processes gene sets in stromal cells as in (C).

(F) CXCL8 concentration in slice culture supernatants after 20 h without (Ctrl) or with adjuvant LMQ (LMQ) (n = 6, each point is the average of three to four slices from one donor), *p < 0.05 by Wilcoxon matched-pairs signed rank test.

(G) UMAP plot of sub-clustered NHSCs from human LN slices colored by cluster and predicted stromal cell type.

(H) Dot plot showing expression of stromal/endothelial marker genes and TLR4 receptor components for each NHSC cluster. Color indicates relative log-normalized level of expression across clusters and dot size the proportion of each cluster expressing each gene.

(I) Left, relative expression of CXCL2 (top) and CXCL8 (bottom) across NHSC clusters, and right, median transcript expression across NHSC cell types in human LN slices without (Ctrl; magenta) or with LMQ adjuvant (LMQ; lavender), each dot represents an individual donor.

See also Figure S5 and Tables S3 and S4.

However, a DC maturation signature (upregulation of *CCR7*, *CD83*, *IDO1*, *ICAM1*) was evident within the Mon./Mac. cluster, and LMQ has been shown to induce the differentiation of human monocytes into DCs, with a Th1-polarizing and $CD8^+$ T cell priming potential in 2D *in vitro* models.[4] Indeed, AS01 has been described to induce preferential activation of monocytes over myeloid DCs in human peripheral blood in the absence of antigen.[73] Together, this could indicate a role for monocyte-derived DCs in adjuvant-driven T cell responses within the LN.

This study also highlights both direct and indirect activation of LN NHSCs, which are generally neglected in studies of vaccine responses. Clear upregulation of chemokines such as *CXCL2* and *CXCL3* in mesenchymal stromal and ECs indicate their pivotal role in cell recruitment and amplification of the inflammatory response. While in mice TLR4 expression on stromal cells was suggested to be dispensable for optimal adjuvant responses, the impact of TLR4 deletion within the stromal compartment specifically on cell recruitment was not assessed, although a stromal contribution to DC maturation was noted.[64] We observed upregulated expression of *TSLP* in stromal cells, a cytokine shown to activate DCs,[74] as well as *IL33*, an alarmin critical for anti-viral T cell responses.[75] We also show ILC3s to be the major resident non-T/B cell population in the human LNs studied, activated by adjuvant to produce IL-22. IL-22 has been described to promote chemokine expression in the stromal cells of tertiary lymphoid organs important in regulating B cell responses,[76] offering another mechanism by which adjuvants may be impacting humoral adaptive immunity. Anatomical differences in the distribution of ILC subtypes have been described,[40,56] and also of other resident and migratory cell types in LNs draining distinct organs or regions.[77,78] Systematic assessment of heterogeneity in immune pathways of LNs of different location and immune history, and particularly those draining standard vaccination sites, would be beneficial, with human LN slice cultures offering a platform through which this could be achieved.

LMQ-induced levels of IL-22 in LN slice supernatants showed donor variability proportional to the $IL2RA^+IL22^+$ ILC3 population present in each donor. The induction of inflammatory cytokines was also variable between donors, with some displaying higher baseline cytokine levels. Preserving individual immune variation is important in accurately recapitulating human responses, such as demographic differences (e.g., with age) or inherently among individuals. Some donors are described to have a "naturally adjuvanted state,"[63] which may be due to past or current infections and cannot be easily replicated in animal models. Importantly, tissue slices enable personalized assessment of the response to immune intervention through capturing pre-treatment (baseline) and post-treatment responses within the same individual. This allows intrinsic differences and infection histories to be taken into account when assessing response to therapy or vaccination, and the generation of donor-specific immune profiles, facilitating stratification of novel therapies and formulations to the individuals most likely to respond. Testing of responses directly in patients could be achieved through use of precision slices from tissue biopsies, in tissues beyond the LN, which could also enable identification of therapy responders vs. non-responders. Use of a system such as this in deciphering differential responses across the human population will be key in retaining the impact of the tissue microenvironment and organization on responses as important parameters in vaccination,[25] cancer,[79] or inflammatory disease settings.[80]

The *ex vivo* human LN slice approach described here offers a versatile platform for studying mode of action and early cell signaling, and could be applied to a variety of compounds, from novel small molecule drugs to a range of immunostimulants and existing therapeutics. Evaluating responses to immune or inflammatory perturbation on a per donor basis and building our understanding of the principles and nuances of the underlying mechanisms could pave the way for rational design of vaccines and drugs toward achieving precision medicine.

Limitations of the study

While this method enables modeling of immune responses in humans, and could provide a means of testing candidate drugs, healthy human tissue can be difficult to acquire and access is limited, which would restrict the use of such approaches in drug screening strategies. Further, the type of LN obtained may not be the most relevant for the scientific question, with the extent of variation between LNs of different anatomical sites not yet fully defined but likely to impact responses.[77] In the cystic LNs used in this study, ILC3s dominated non-T/B cell populations, which may not be true of LNs draining conventional immunization sites.[40,56] Our analysis of stromal cell subsets was also limited to small cell numbers, partly as a result of ILC dominance, which limited our analysis and characterization of stromal subtypes. Further limitations of this study, and which may account for the lack of observed T cell and DC activation compared with studies of AS01 in mice, include the absence of antigen, which could be added to cultures in future studies. Indeed, slices of mouse LNs have previously been shown to process whole-protein antigen added to culture, and to respond to vaccine antigen *in vitro* following previous *in vivo* vaccination.[31] *Ex vivo* tissue viability over a longer time (days to weeks), which would enable assaying of the adaptive responses to antigen, remains to be fully determined, and will likely require further optimization of culture conditions such as media perfusion to prevent tissue hypoxia for extended periods in culture.[81] Additional optimization of culture media may also improve retention of macrophages within tissue slices, for which a reduction was observed after culture. It also should be noted that this is a static system, with cell populations uniformly exposed to the adjuvant, rather than temporally through drainage via LN sinuses and conduits.[82] Similarly, the effects of cell recruitment cannot be observed, limiting the study to resident cell types. Soluble antigen, including adjuvant,[34] has been shown to reach the dLN within minutes, whereas influx of migrating cells takes several hours to many days,[36,83,84] implicating resident cells as drivers of the earliest immune responses. One exception to this are neutrophils, with antigen-bearing neutrophils found in the dLN within the first few hours post vaccination.[36,85] Although neutrophils were detected in the LNs at baseline and after slice culture, they were not retained during sample preparation for transcriptomic analysis, meaning that their specific gene-expression contribution could not be examined. Analysis of neutrophils by scRNA-seq is widely recognized as technically difficult,[45] although application of alternative sequencing platforms may improve capture.[86]

RESOURCE AVAILABILITY

Lead contact

Requests for further information and resources should be directed to and will be fulfilled by the lead contact, Anita Milicic (anita.milicic@ndm.ox.ac.uk).

Materials availability

This study did not generate new unique reagents. VFI adjuvant LMQ can be made available through contact via the VFI website (https://www.vaccineformulationinstitute.org/).

Data and code availability

Single-cell RNA-seq data have been deposited at GEO: GSE294959 and are available through CELLxGENE Discover (“LNCS: Atlas of healthy human lymph nodes with and without *ex vivo* adjuvant stimulation”). They are publicly available as of the date of publication. All original code has been deposited at Github with DOI 10.5281/zenodo.15387998 (https://github.com/DendrouLab/LymphNodeCultureSlice_2025CellRep) for single-cell RNA-seq analyses and https://zenodo.org/records/15396757 (https://github.com/edwardjenkins1/2D_spatial_analysis_lymph_node) for image analyses, and is publicly available as of the date of publication. Microscopy data and flow cytometry data reported in this paper will be shared by the lead contact upon request. Any additional information required to reanalyze the data reported in this paper is available from the lead contact upon request.

ACKNOWLEDGMENTS

Adjuvant used in this study was provided by the Vaccine Formulation Institute, Switzerland. Sample collection was supported by NIHR Biomedical Research Center, Oxford. The views expressed are those of the authors and not necessarily those of the NHS, the NIHR or the Department of Health. We thank the Oxford Translational Gastroenterology Unit Investigators for their help in sample collection, and to Paul Klenerman and Nick Provine for help with obtaining ethical approval. We are grateful to Ryan Waters for provision of pig lymph nodes, and to the Kennedy Institute facility staff including the Flow Facility, Single Cell Facility, Digital Pathology Omics Core, Oxford Zeiss Centre of Excellence and the Histology Facility. We acknowledge the generous support of the Kennedy Trust for Rheumatology Research for the provision of flow cytometry, single-cell genomics, and microscopy facilities used in this research. The authors are grateful to Rowie Borst for lab management, Sam Pledger for lab management and technical assistance, Romain Guyon for helpful discussions and statistical help, Sarah Davidson for assists throughout the project, Zhi Wong for project support and ideas, Carl Lee for all-round help, Floriane Auderset and Marcelle van Mechelen for critical review of the manuscript, and Jelena Bezbradica Mirkovic for help with project discussions and interpretation of inflammasome results. Figure schematics were made with BioRender.

Chan Zuckerberg Initiative grant 2020-217289 (to J.R.F., J.H.Y.S., M.C., C.A.D., and A.M.); UK Medical Research Council MR/Y004450/1 (to J.R.F. and M.C.); Wellcome Trust Fellowship 226938/Z/23/Z (J.H.Y.S.); Wellcome Trust Fellowship 224040/Z/21/Z (E.J.); German Research Foundation/Deutsche Forschungsgemeinschaft 495054088 (to S.R.); Wellcome Trust grant HMR05310 (to S.H.); Wellcome Trust and Royal Society 204290/Z/16/Z (to C.A.D.); John Fell Fund grant (to A.M.); Bill and Melinda Gates Foundation “Adjuvants for Global Health” grant INV001759 (to T.C. and A.M.); UK Medical Research Council MR/T030410/1 (to C.A.D.); UK Medical Research Council MR/XO12093/1 (to N.G. and A.B.); Rosetrees Trust R35579/AA002/M85-F2 (to C.A.D.); Janssen Biotech, Inc Cartography Consortium (to C.A.D.); NIHR Biomedical Research Center, Inflammation Across Tissues Theme (to J.F. and C.A.D.); Kennedy Trust for Rheumatology Research KENN 20 21 17 (to M.A. and M.L.D.).

AUTHOR CONTRIBUTIONS

Conceptualization: J.R.F., M.C., C.A.D., and A.M.; data curation: J.H.Y.S. and C.A.D.; formal analysis: J.R.F., J.H.Y.S., N.G., E.J., and T.S.; methodology: J.R.F.; investigation: J.R.F., E.N., S.R., A.B., S.M.K., S.H., and M.A.; visualization: J.R.F., J.H.Y.S., E.J., and N.G.; resources: T.C. and A.G.-W.; funding acquisition: M.L.D., M.C., C.A.D., and A.M.; supervision: M.C., C.A.D., and A.M.; writing – original draft: J.R.F. and A.M.; writing – review & editing: J.R.F., J.H.Y.S., E.J., A.G.-W., M.C., C.A.D., and A.M.

DECLARATION OF INTERESTS

M.C. has founders shares in Mestag Therapeutics Limited.

STAR★METHODS

Detailed methods are provided in the online version of this paper and include the following:

- KEY RESOURCES TABLE
- EXPERIMENTAL MODEL AND STUDY PARTICIPANT DETAILS
  - Human Participants
  - Animals
- METHOD DETAILS
  - Materials, including adjuvant
  - Precision lymph node slicing and culture
  - Mouse experiments
  - Single cell RNA sequencing
  - Multiplexed fluorescence imaging
  - Supernatant analysis by LEGENDPlex and ELISA
  - Flow cytometry
- QUANTIFICATION AND STATISTICAL ANALYSIS

SUPPLEMENTAL INFORMATION

Supplemental information can be found online at https://doi.org/10.1016/j.celrep.2025.115938.

Received: November 6, 2024
Revised: March 13, 2025
Accepted: June 9, 2025
Published: July 2, 2025

REFERENCES

1. Medetgul-Ernar, K., and Davis, M.M. (2022). Standing on the shoulders of mice. Immunity *55*, 1343–1353. https://doi.org/10.1016/j.immuni.2022.07.008.

2. Morrocchi, E., van Haren, S., Palma, P., and Levy, O. (2024). Modeling human immune responses to vaccination in vitro. Trends Immunol. *45*, 32–47. https://doi.org/10.1016/j.it.2023.11.002.

3. Pulendran, B., S Arunachalam, P., and O’Hagan, D.T. (2021). Emerging concepts in the science of vaccine adjuvants. Nat. Rev. Drug Discov. *20*, 454–475. https://doi.org/10.1038/s41573-021-00163-y.

4. Doss-Gollin, S., Thomas, S., Brook, B., Abedi, K., Lebas, C., Auderset, F., Lugo-Rodriguez, Y., Sanchez-Schmitz, G., Dowling, D.J., Levy, O., and van Haren, S.D. (2023). Human in vitro modeling of adjuvant formulations demonstrates enhancement of immune responses to SARS-CoV-2 antigen. NPJ Vaccines *8*, 163. https://doi.org/10.1038/s41541-023-00759-y.

5. Chaicharoenaudomrung, N., Kunhorm, P., and Noisa, P. (2019). Three-dimensional cell culture systems as an in vitro platform for cancer and stem cell modeling. World J. Stem Cells *11*, 1065–1083. https://doi.org/10.4252/wjsc.v11.i12.1065.

6. Turner, J.S., O’Halloran, J.A., Kalaidina, E., Kim, W., Schmitz, A.J., Zhou, J.Q., Lei, T., Thapa, M., Chen, R.E., Case, J.B., et al. (2021). SARS-CoV-2 mRNA vaccines induce persistent human germinal centre responses. Nature *596*, 109–113. https://doi.org/10.1038/s41586-021-03738-2.

7. Lederer, K., Bettini, E., Parvathaneni, K., Painter, M.M., Agarwal, D., Lundgreen, K.A., Weirick, M., Muralidharan, K., Castaño, D., Goel, R.R., et al. (2022). Germinal center responses to SARS-CoV-2 mRNA vaccines in

healthy and immunocompromised individuals. Cell *185*, 1008–1024.e15. https://doi.org/10.1016/j.cell.2022.01.027.

8. Turner, J.S., Zhou, J.Q., Han, J., Schmitz, A.J., Rizk, A.A., Alsoussi, W.B., Lei, T., Amor, M., McIntire, K.M., Meade, P., et al. (2020). Human germinal centres engage memory and naive B cells after influenza vaccination. Nature *586*, 127–132. https://doi.org/10.1038/s41586-020-2711-0.
9. Day, S., Kaur, C., Cheeseman, H.M., de Groot, E., McFarlane, L.R., Tanaka, M., Coelho, S., Cole, T., Lemm, N.M., Lim, A., et al. (2022). Comparison of blood and lymph node cells after intramuscular injection with HIV envelope immunogens. Front. Immunol. *13*, 991509. https://doi.org/10.3389/fimmu.2022.991509.
10. Damato, V., Theorell, J., Al-Diwani, A., Kienzler, A.K., Makuch, M., Sun, B., Handel, A., Akdeniz, D., Berretta, A., Ramanathan, S., et al. (2022). Rituximab abrogates aquaporin-4-specific germinal center activity in patients with neuromyelitis optica spectrum disorders. Proc. Natl. Acad. Sci. USA *119*, e2121804119. https://doi.org/10.1073/pnas.2121804119.
11. Al-Diwani, A., Theorell, J., Damato, V., Bull, J., McGlashan, N., Green, E., Kienzler, A.K., Harrison, R., Hassanali, T., Campo, L., et al. (2022). Cervical lymph nodes and ovarian teratomas as germinal centres in NMDA receptor-antibody encephalitis. Brain *145*, 2742–2754. https://doi.org/10.1093/brain/awac088.
12. Provine, N.M., Al-Diwani, A., Agarwal, D., Dooley, K., Heslington, A., Murchison, A.G., Garner, L.C., Sheerin, F., Klenerman, P., and Irani, S.R. (2024). Fine needle aspiration of human lymph nodes reveals cell populations and soluble interactors pivotal to immunological priming. Eur. J. Immunol. *54*, e2350872. https://doi.org/10.1002/eji.202350872.
13. Krishnamurty, A.T., and Turley, S.J. (2020). Lymph node stromal cells: cartographers of the immune system. Nat. Immunol. *21*, 369–380. https://doi.org/10.1038/s41590-020-0635-3.
14. Leal, J.M., Huang, J.Y., Kohli, K., Stoltzfus, C., Lyons-Cohen, M.R., Olin, B.E., Gale, M., Jr., and Gerner, M.Y. (2021). Innate cell microenvironments in lymph nodes shape the generation of T cell responses during type I inflammation. Sci. Immunol. *6*, eabb9435. https://doi.org/10.1126/sciimmunol.abb9435.
15. Szakal, A.K., Kosco, M.H., and Tew, J.G. (1989). Microanatomy of lymphoid tissue during humoral immune responses: structure function relationships. Annu. Rev. Immunol. *7*, 91–109. https://doi.org/10.1146/annurev.iy.07.040189.000515.
16. van Rooijen, N. (1990). Antigen processing and presentation in vivo: the microenvironment as a crucial factor. Immunol. Today *11*, 436–439. https://doi.org/10.1016/0167-5699(90)90171-5.
17. Giese, C., Demmler, C.D., Ammer, R., Hartmann, S., Lubitz, A., Miller, L., Müller, R., and Marx, U. (2006). A human lymph node in vitro–challenges and progress. Artif. Organs *30*, 803–808. https://doi.org/10.1111/j.1525-1594.2006.00303.x.
18. Wagar, L.E., Salahudeen, A., Constantz, C.M., Wendel, B.S., Lyons, M.M., Mallajosyula, V., Jatt, L.P., Adamska, J.Z., Blum, L.K., Gupta, N., et al. (2021). Modeling human adaptive immune responses with tonsil organoids. Nat. Med. *27*, 125–135. https://doi.org/10.1038/s41591-020-01145-0.
19. Ferro, L.M., Weedon, H.M., Flego, L.R., Beroukas, D., and Zola, H. (1993). An organ fragment culture model to study lymphocyte activation in human lymphoid tissue. Immunobiology *188*, 51–61. https://doi.org/10.1016/S0171-2985(11)80486-9.
20. Grivel, J.C., and Margolis, L. (2009). Use of human tissue explants to study human infectious agents. Nat. Protoc. *4*, 256–269. https://doi.org/10.1038/nprot.2008.245.
21. Hoffmann, P., Skibinski, G., and James, K. (1995). Organ culture of human lymphoid tissue. I. Characteristics of the system. J. Immunol. Methods *179*, 37–49. https://doi.org/10.1016/0022-1759(94)00268-2.
22. Skibinski, G., Hoffmann, P., Radbruch, A., and James, K. (1997). Organ culture of human lymphoid tissue. II. Marked differences in cytokine production and proliferation between slice and suspension cultures of human spleen. J. Immunol. Methods *205*, 115–125. https://doi.org/10.1016/s0022-1759(97)00058-6.
23. Stylianou, V.V., Bertram, K.M., Vo, V.A., Dunn, E.B., Baharlou, H., Terre, D.J., Elhindi, J., Elder, E., French, J., Meybodi, F., et al. (2024). Innate immune cell activation by adjuvant AS01 in human lymph node explants is age independent. J. Clin. Investig. *134*, e174144. https://doi.org/10.1172/jci174144.
24. Glushakova, S., Baibakov, B., Margolis, L.B., and Zimmerberg, J. (1995). Infection of human tonsil histocultures: A model for HIV pathogenesis. Nat. Med. *1*, 1320–1322. https://doi.org/10.1038/nm1295-1320.
25. Cupovic, J., Ring, S.S., Onder, L., Colston, J.M., Lütge, M., Cheng, H.W., De Martin, A., Provine, N.M., Flatz, L., Oxenius, A., et al. (2021). Adenovirus vector vaccination reprograms pulmonary fibroblastic niches to support protective inflating memory CD8(+) T cells. Nat. Immunol. *22*, 1042–1051. https://doi.org/10.1038/s41590-021-00969-3.
26. Palma, E., Doornebal, E.J., and Chokshi, S. (2019). Precision-cut liver slices: a versatile tool to advance liver research. Hepatol. Int. *13*, 51–57. https://doi.org/10.1007/s12072-018-9913-7.
27. Viana, F., O'Kane, C.M., and Schroeder, G.N. (2022). Precision-cut lung slices: A powerful ex vivo model to investigate respiratory infectious diseases. Mol. Microbiol. *117*, 578–588. https://doi.org/10.1111/mmi.14817.
28. Espie, D., Barrin, S., Rajnpreht, I., Vimeux, L., and Donnadieu, E. (2023). Live Imaging of CAR T Cell Ca2+ Signals in Tumor Slices Using Confocal Microscopy. In Methods in Molecular Biology (Springer US)), pp. 453–462. https://doi.org/10.1007/978-1-0716-3135-5_29.
29. Knoblich, K., Cruz Migoni, S., Siew, S.M., Jinks, E., Kaul, B., Jeffery, H.C., Baker, A.T., Suliman, M., Vrzalikova, K., Mehenna, H., et al. (2018). The human lymph node microenvironment unilaterally regulates T-cell activation and differentiation. PLoS Biol. *16*, e2005046. https://doi.org/10.1371/journal.pbio.2005046.
30. Donnadieu, E., Michel, Y., and Hansmann, M.-L. (2019). Live Imaging of Resident T-Cell Migration in Human Lymphoid Tissue Slices Using Confocal Microscopy. In Methods in Molecular Biology (New York: Springer), pp. 75–82. https://doi.org/10.1007/978-1-4939-9036-8_10.
31. Belanger, M.C., Ball, A.G., Catterton, M.A., Kinman, A.W.L., Anbaei, P., Groff, B.D., Melchor, S.J., Lukens, J.R., Ross, A.E., and Pompano, R.R. (2021). Acute Lymph Node Slices Are a Functional Model System to Study Immunity Ex Vivo. ACS Pharmacol. Transl. Sci. *4*, 128–142. https://doi.org/10.1021/acsptsci.0c00143.
32. Ball, A.G., Belanger, M.C., and Pompano, R.R. (2021). Detergent wash improves vaccinated lymph node handling ex vivo. J. Immunol. Methods *489*, 112943. https://doi.org/10.1016/j.jim.2020.112943.
33. Coccia, M., Collignon, C., Herve, C., Chalon, A., Welsby, I., Detienne, S., van Helden, M.J., Dutta, S., Genito, C.J., Waters, N.C., et al. (2017). Cellular and molecular synergy in AS01-adjuvanted vaccines results in an early IFNgamma response promoting vaccine immunogenicity. NPJ Vaccines *2*, 25. https://doi.org/10.1038/s41541-017-0027-3.
34. Detienne, S., Welsby, I., Collignon, C., Wouters, S., Coccia, M., Delhaye, S., Van Maele, L., Thomas, S., Swertvaegher, M., Detavernier, A., et al. (2016). Central Role of CD169(+) Lymph Node Resident Macrophages in the Adjuvanticity of the QS-21 Component of AS01. Sci. Rep. *6*, 39475. https://doi.org/10.1038/srep39475.
35. Neeland, M.R., Shi, W., Collignon, C., Taubenheim, N., Meeusen, E.N.T., Didierlaurent, A.M., and de Veer, M.J. (2016). The Lymphatic Immune Response Induced by the Adjuvant AS01: A Comparison of Intramuscular and Subcutaneous Immunization Routes. J. Immunol. *197*, 2704–2714. https://doi.org/10.4049/jimmunol.1600817.
36. Didierlaurent, A.M., Collignon, C., Bourguignon, P., Wouters, S., Fierens, K., Fochesato, M., Dendouga, N., Langlet, C., Malissen, B., Lambrecht, B.N., et al. (2014). Enhancement of adaptive immunity by the human vaccine adjuvant AS01 depends on activated dendritic cells. J. Immunol. *193*, 1920–1930. https://doi.org/10.4049/jimmunol.1400948.

37. Bechtold, V., Smolen, K.K., Burny, W., de Angelis, S.P., Delandre, S., Essaghir, A., Marchant, A., Ndour, C., Taton, M., van der Most, R., et al. (2024). Functional and epigenetic changes in monocytes from adults immunized with an AS01-adjuvanted vaccine. Sci. Transl. Med. *16*, eadl3381. https://doi.org/10.1126/scitranslmed.adl3381.

38. Reinke, S., Pantazi, E., Chappell, G.R., Sanchez-Martinez, A., Guyon, R., Fergusson, J.R., Salman, A.M., Aktar, A., Mukhopadhyay, E., Ventura, R. A., et al. (2023). Emulsion and liposome-based adjuvanted R21 vaccine formulations mediate protection against malaria through distinct immune mechanisms. Cell Rep. Med. *4*, 101245. https://doi.org/10.1016/j.xcrm.2023.101245.

39. O'Donnell, J.S., Isaacs, A., Jakob, V., Lebas, C., Barnes, J.B., Reading, P.C., Young, P.R., Watterson, D., Dubois, P.M., Collin, N., and Chappell, K.J. (2022). Characterization and comparison of novel adjuvants for a prefusion clamped MERS vaccine. Front. Immunol. *13*, 976968. https://doi.org/10.3389/fimmu.2022.976968.

40. Yudanin, N.A., Schmitz, F., Flamar, A.L., Thome, J.J.C., Tait Wojno, E., Moeller, J.B., Schirmer, M., Latorre, I.J., Xavier, R.J., Farber, D.L., et al. (2019). Spatial and Temporal Mapping of Human Innate Lymphoid Cells Reveals Elements of Tissue Specificity. Immunity *50*, 505–519.e4. https://doi.org/10.1016/j.immuni.2019.01.012.

41. Bjorklund, A.K., Forkel, M., Picelli, S., Konya, V., Theorell, J., Friberg, D., Sandberg, R., and Mjosberg, J. (2016). The heterogeneity of human CD127(+) innate lymphoid cells revealed by single-cell RNA sequencing. Nat. Immunol. *17*, 451–460. https://doi.org/10.1038/ni.3368.

42. Spits, H., Artis, D., Colonna, M., Diefenbach, A., Di Santo, J.P., Eberl, G., Koyasu, S., Locksley, R.M., McKenzie, A.N.J., Mebius, R.E., et al. (2013). Innate lymphoid cells–a proposal for uniform nomenclature. Nat. Rev. Immunol. *13*, 145–149. https://doi.org/10.1038/nri3365.

43. Bernink, J.H., Krabbendam, L., Germar, K., de Jong, E., Gronke, K., Kofoed-Nielsen, M., Munneke, J.M., Hazenberg, M.D., Villaudy, J., Buskens, C.J., et al. (2015). Interleukin-12 and -23 Control Plasticity of CD127(+) Group 1 and Group 3 Innate Lymphoid Cells in the Intestinal Lamina Propria. Immunity *43*, 146–160. https://doi.org/10.1016/j.immuni.2015.06.019.

44. Jaeger, N., Antonova, A.U., Kreisel, D., Roan, F., Lantelme, E., Ziegler, S.F., Cella, M., and Colonna, M. (2024). Diversity of group 1 innate lymphoid cells in human tissues. Nat. Immunol. *25*, 1460–1473. https://doi.org/10.1038/s41590-024-01885-y.

45. Wigerblad, G., Cao, Q., Brooks, S., Naz, F., Gadkari, M., Jiang, K., Gupta, S., O'Neil, L., Dell'Orso, S., Kaplan, M.J., and Franco, L.M. (2022). Single-Cell Analysis Reveals the Range of Transcriptional States of Circulating Human Neutrophils. J. Immunol. *209*, 772–782. https://doi.org/10.4049/jimmunol.2200154.

46. Blanter, M., Gouwy, M., and Struyf, S. (2021). Studying Neutrophil Function in vitro: Cell Models and Environmental Factors. J. Inflamm. Res. *14*, 141–162. https://doi.org/10.2147/JIR.S284941.

47. Burny, W., Callegaro, A., Bechtold, V., Clement, F., Delhaye, S., Fissette, L., Janssens, M., Leroux-Roels, G., Marchant, A., van den Berg, R.A., et al. (2017). Different Adjuvants Induce Common Innate Pathways That Are Associated with Enhanced Adaptive Responses against a Model Antigen in Humans. Front. Immunol. *8*, 943. https://doi.org/10.3389/fimmu.2017.00943.

48. Suscovich, T.J., Fallon, J.K., Das, J., Demas, A.R., Crain, J., Linde, C.H., Michell, A., Natarajan, H., Arevalo, C., Broge, T., et al. (2020). Mapping functional humoral correlates of protection against malaria challenge following RTS,S/AS01 vaccination. Sci. Transl. Med. *12*, eabb4757. https://doi.org/10.1126/scitranslmed.abb4757.

49. Marty-Roix, R., Vladimer, G.I., Pouliot, K., Weng, D., Buglione-Corbett, R., West, K., MacMicking, J.D., Chee, J.D., Wang, S., Lu, S., and Lien, E. (2016). Identification of QS-21 as an Inflammasome-activating Molecular Component of Saponin Adjuvants. J. Biol. Chem. *291*, 1123–1136. https://doi.org/10.1074/jbc.M115.683011.

50. Andreatta, M., and Carmona, S.J. (2021). UCell: Robust and scalable single-cell gene signature scoring. Comput. Struct. Biotechnol. J. *19*, 3796–3798. https://doi.org/10.1016/j.csbj.2021.06.043.

51. Bauernfeind, F.G., Horvath, G., Stutz, A., Alnemri, E.S., MacDonald, K., Speert, D., Fernandes-Alnemri, T., Wu, J., Monks, B.G., Fitzgerald, K.A., et al. (2009). Cutting edge: NF-kappaB activating pattern recognition and cytokine receptors license NLRP3 inflammasome activation by regulating NLRP3 expression. J. Immunol. *183*, 787–791. https://doi.org/10.4049/jimmunol.0901363.

52. He, Y., Hara, H., and Núñez, G. (2016). Mechanism and Regulation of NLRP3 Inflammasome Activation. Trends Biochem. Sci. *41*, 1012–1021. https://doi.org/10.1016/j.tibs.2016.09.002.

53. Ii, M., Matsunaga, N., Hazeki, K., Nakamura, K., Takashima, K., Seya, T., Hazeki, O., Kitazaki, T., and Iizawa, Y. (2006). A novel cyclohexene derivative, ethyl (6R)-6-[N-(2-Chloro-4-fluorophenyl)sulfamoyl]cyclohex-1-ene-1-carboxylate (TAK-242), selectively inhibits toll-like receptor 4-mediated cytokine production through suppression of intracellular signaling. Mol. Pharmacol. *69*, 1288–1295. https://doi.org/10.1124/mol.105.019695.

54. Coll, R.C., Robertson, A.A.B., Chae, J.J., Higgins, S.C., Muñoz-Planillo, R., Inserra, M.C., Vetter, I., Dungan, L.S., Monks, B.G., Stutz, A., et al. (2015). A small-molecule inhibitor of the NLRP3 inflammasome for the treatment of inflammatory diseases. Nat. Med. *21*, 248–255. https://doi.org/10.1038/nm.3806.

55. Puren, A.J., Fantuzzi, G., and Dinarello, C.A. (1999). Gene expression, synthesis, and secretion of interleukin 18 and interleukin 1β are differentially regulated in human blood mononuclear cells and mouse spleen cells. Proc. Natl. Acad. Sci. USA *96*, 2256–2261. https://doi.org/10.1073/pnas.96.5.2256.

56. Bar-Ephraim, Y.E., Cornelissen, F., Papazian, N., Konijn, T., Hoogenboezem, R.M., Sanders, M.A., Westerman, B.A., Gönültas, M., Kwekkeboom, J., Den Haan, J.M.M., et al. (2017). Cross-Tissue Transcriptomic Analysis of Human Secondary Lymphoid Organ-Residing ILC3s Reveals a Quiescent State in the Absence of Inflammation. Cell Rep. *21*, 823–833. https://doi.org/10.1016/j.celrep.2017.09.070.

57. Li, Z., Jackson, R.J., and Ranasinghe, C. (2018). Vaccination route can significantly alter the innate lymphoid cell subsets: a feedback between IL-13 and IFN-gamma. NPJ Vaccines *3*, 10. https://doi.org/10.1038/s41541-018-0048-6.

58. Okamura, H., Nagata, K., Komatsu, T., Tanimoto, T., Nukata, Y., Tanabe, F., Akita, K., Torigoe, K., Okura, T., Fukuda, S., et al. (1995). A novel costimulatory factor for gamma interferon induction found in the livers of mice causes endotoxic shock. Infect. Immun. *63*, 3966–3972. https://doi.org/10.1128/iai.63.10.3966-3972.1995.

59. Micallef, M.J., Ohtsuki, T., Kohno, K., Tanabe, F., Ushio, S., Namba, M., Tanimoto, T., Torigoe, K., Fujii, M., Ikeda, M., et al. (1996). Interferon-gamma-inducing factor enhances T helper 1 cytokine production by stimulated human T cells: synergism with interleukin-12 for interferon-gamma production. Eur. J. Immunol. *26*, 1647–1651. https://doi.org/10.1002/eji.1830260736.

60. Ziblat, A., Nuñez, S.Y., Raffo Iraolagoitia, X.L., Spallanzani, R.G., Torres, N.I., Sierra, J.M., Secchiari, F., Domaica, C.I., Fuertes, M.B., and Zwirner, N.W. (2018). Interleukin (IL)-23 Stimulates IFN-γ Secretion by CD56bright Natural Killer Cells and Enhances IL-18-Driven Dendritic Cells Activation. Front. Immunol. *8*. https://doi.org/10.3389/fimmu.2017.01959.

61. Dutton, E.E., Gajdasik, D.W., Willis, C., Fiancette, R., Bishop, E.L., Camelo, A., Sleeman, M.A., Coccia, M., Didierlaurent, A.M., Tomura, M., et al. (2019). Peripheral lymph nodes contain migratory and resident innate lymphoid cell populations. Sci. Immunol. *4*, eaau8082. https://doi.org/10.1126/sciimmunol.aau8082.

62. Centofanti, E., Wang, C., Iyer, S., Krichevsky, O., Oyler-Yaniv, A., and Oyler-Yaniv, J. (2023). The spread of interferon-γ in melanomas is highly spatially confined, driving nongenetic variability in tumor cells. Proc.

Natl. Acad. Sci. USA *120*, e2304190120. https://doi.org/10.1073/pnas.2304190120.

63. Mule, M.P., Martins, A.J., Cheung, F., Farmer, R., Sellers, B.A., Quiel, J.A., Jain, A., Kotliarov, Y., Bansal, N., Chen, J., et al. (2024). Integrating population and single-cell variations in vaccine responses identifies a naturally adjuvanted human immune setpoint. Immunity *57*, 1160–1176.e67. https://doi.org/10.1016/j.immuni.2024.04.009.
64. Van Maele, L., Fougeron, D., Cayet, D., Chalon, A., Piccioli, D., Collignon, C., Sirard, J.C., and Didierlaurent, A.M. (2019). Toll-like receptor 4 signaling in hematopoietic-lineage cells contributes to the enhanced activity of the human vaccine adjuvant AS01. Eur. J. Immunol. *49*, 2134–2145. https://doi.org/10.1002/eji.201948234.
65. Efremova, M., Vento-Tormo, M., Teichmann, S.A., and Vento-Tormo, R. (2020). CellPhoneDB: inferring cell-cell communication from combined expression of multi-subunit ligand-receptor complexes. Nat. Protoc. *15*, 1484–1506. https://doi.org/10.1038/s41596-020-0292-x.
66. Abe, Y., Sakata-Yanagimoto, M., Fujisawa, M., Miyoshi, H., Suehara, Y., Hattori, K., Kusakabe, M., Sakamoto, T., Nishikii, H., Nguyen, T.B., et al. (2022). A single-cell atlas of non-haematopoietic cells in human lymph nodes and lymphoma reveals a landscape of stromal remodelling. Nat. Cell Biol. *24*, 565–578. https://doi.org/10.1038/s41556-022-00866-3.
67. Takeda, A., Hollmen, M., Dermadi, D., Pan, J., Brulois, K.F., Kaukonen, R., Lonnberg, T., Bostrom, P., Koskivuo, I., Irjala, H., et al. (2019). Single-Cell Survey of Human Lymphatics Unveils Marked Endothelial Cell Heterogeneity and Mechanisms of Homing for Neutrophils. Immunity *51*, 561–572.e5. https://doi.org/10.1016/j.immuni.2019.06.027.
68. Pegu, A., Qin, S., Fallert Junecko, B.A., Nisato, R.E., Pepper, M.S., and Reinhart, T.A. (2008). Human lymphatic endothelial cells express multiple functional TLRs. J. Immunol. *180*, 3399–3405. https://doi.org/10.4049/jimmunol.180.5.3399.
69. Heesters, B.A., van Megesen, K., Tomris, I., de Vries, R.P., Magri, G., and Spits, H. (2021). Characterization of human FDCs reveals regulation of T cells and antigen presentation to B cells. J. Exp. Med. *218*, e20210790. https://doi.org/10.1084/jem.20210790.
70. Pulendran, B. (2014). Systems vaccinology: Probing humanity's diverse immune systems with vaccines. Proc. Natl. Acad. Sci. USA *111*, 12300–12306. https://doi.org/10.1073/pnas.1400476111.
71. Bucasas, K.L., Franco, L.M., Shaw, C.A., Bray, M.S., Wells, J.M., Niño, D., Arden, N., Quarles, J.M., Couch, R.B., and Belmont, J.W. (2011). Early patterns of gene expression correlate with the humoral immune response to influenza vaccination in humans. J. Infect. Dis. *203*, 921–929. https://doi.org/10.1093/infdis/jiq156.
72. Arunachalam, P.S., Ha, N., Dennison, S.M., Spreng, R.L., Seaton, K.E., Xiao, P., Feng, Y., Zarnitsyna, V.I., Kazmin, D., Hu, M., et al. (2024). A comparative immunological assessment of multiple clinical-stage adjuvants for the R21 malaria vaccine in nonhuman primates. Sci. Transl. Med. *16*, eadn6605. https://doi.org/10.1126/scitranslmed.adn6605.
73. Smith, C.L., Richardson, B., Rubsamen, M., Cameron, M.J., Cameron, C.M., and Canaday, D.H. (2024). Adjuvant AS01 activates human monocytes for costimulation and systemic inflammation. Vaccine *42*, 229–238. https://doi.org/10.1016/j.vaccine.2023.12.010.
74. Soumelis, V., Reche, P.A., Kanzler, H., Yuan, W., Edward, G., Homey, B., Gilliet, M., Ho, S., Antonenko, S., Lauerma, A., et al. (2002). Human epithelial cells trigger dendritic cell mediated allergic inflammation by producing TSLP. Nat. Immunol. *3*, 673–680. https://doi.org/10.1038/ni805.
75. Aparicio-Domingo, P., Cannelle, H., Buechler, M.B., Nguyen, S., Kallert, S.M., Favre, S., Alouche, N., Papazian, N., Ludewig, B., Cupedo, T., et al. (2021). Fibroblast-derived IL-33 is dispensable for lymph node homeostasis but critical for CD8 T-cell responses to acute and chronic viral infection. Eur. J. Immunol. *51*, 76–90. https://doi.org/10.1002/eji.201948413.
76. Barone, F., Nayar, S., Campos, J., Cloake, T., Withers, D.R., Toellner, K.M., Zhang, Y., Fouser, L., Fisher, B., Bowman, S., et al. (2015). IL-22 regulates lymphoid chemokine production and assembly of tertiary lymphoid organs. Proc. Natl. Acad. Sci. USA *112*, 11024–11029. https://doi.org/10.1073/pnas.1503315112.
77. Cruz De Casas, P., Knöpper, K., Dey Sarkar, R., and Kastenmüller, W. (2024). Same yet different — how lymph node heterogeneity affects immune responses. Nat. Rev. Immunol. *24*, 358–374. https://doi.org/10.1038/s41577-023-00965-8.
78. Esterhazy, D., Canesso, M.C.C., Mesin, L., Muller, P.A., de Castro, T.B.R., Lockhart, A., ElJalby, M., Faria, A.M.C., and Mucida, D. (2019). Compartmentalized gut lymph node drainage dictates adaptive immune responses. Nature *569*, 126–130. https://doi.org/10.1038/s41586-019-1125-3.
79. Klemm, F., and Joyce, J.A. (2015). Microenvironmental regulation of therapeutic response in cancer. Trends Cell Biol. *25*, 198–213. https://doi.org/10.1016/j.tcb.2014.11.006.
80. Thomas, T., Friedrich, M., Rich-Griffin, C., Pohin, M., Agarwal, D., Pakpoor, J., Lee, C., Tandon, R., Rendek, A., Aschenbrenner, D., et al. (2024). A longitudinal single-cell atlas of anti-tumour necrosis factor treatment in inflammatory bowel disease. Nat. Immunol. *25*, 2152–2165. https://doi.org/10.1038/s41590-024-01994-8.
81. Anbaei, P., Stevens, M.G., Ball, A.G., Bullock, T.N.J., and Pompano, R.R. (2024). Spatially resolved quantification of oxygen consumption rate in ex vivo lymph node slices. Analyst *149*, 2609–2620. https://doi.org/10.1039/d4an00028e.
82. Kelch, I.D., Bogle, G., Sands, G.B., Phillips, A.R.J., LeGrice, I.J., and Dunbar, P.R. (2019). High-resolution 3D imaging and topological mapping of the lymph node conduit system. PLoS Biol. *17*, e3000486. https://doi.org/10.1371/journal.pbio.3000486.
83. Itano, A.A., McSorley, S.J., Reinhardt, R.L., Ehst, B.D., Ingulli, E., Rudensky, A.Y., and Jenkins, M.K. (2003). Distinct dendritic cell populations sequentially present antigen to CD4 T cells and stimulate different aspects of cell-mediated immunity. Immunity *19*, 47–57. https://doi.org/10.1016/s1074-7613(03)00175-4.
84. Sixt, M., Kanazawa, N., Selg, M., Samson, T., Roos, G., Reinhardt, D.P., Pabst, R., Lutz, M.B., and Sorokin, L. (2005). The conduit system transports soluble antigens from the afferent lymph to resident dendritic cells in the T cell area of the lymph node. Immunity *22*, 19–29. https://doi.org/10.1016/j.immuni.2004.11.013.
85. Neeland, M.R., Shi, W., Collignon, C., Meeusen, E.N.T., Didierlaurent, A.M., and de Veer, M.J. (2018). The adjuvant system AS01 up-regulates neutrophil CD14 expression and neutrophil-associated antigen transport in the local lymphatic network. Clin. Exp. Immunol. *192*, 46–53. https://doi.org/10.1111/cei.13088.
86. Kwok, A.J., Allcock, A., Ferreira, R.C., Cano-Gamez, E., Smee, M., Burnham, K.L., Zurke, Y.-X., McKechnie, S., Mentzer, A.J., et al.; Emergency Medicine Research Oxford EMROx (2023). Neutrophils and emergency granulopoiesis drive immune suppression and an extreme response endotype during sepsis. Nat. Immunol. *24*, 767–779. https://doi.org/10.1038/s41590-023-01490-5.
87. Curion, F., Rich-Griffin, C., Agarwal, D., Ouologuem, S., Rue-Albrecht, K., May, L., Garcia, G.E.L., Heumos, L., Thomas, T., Lason, W., et al. (2024). Panpipes: a pipeline for multiomic single-cell and spatial transcriptomic data analysis. Genome Biol. *25*, 181. https://doi.org/10.1186/s13059-024-03322-7.
88. Wolf, F.A., Angerer, P., and Theis, F.J. (2018). SCANPY: large-scale single-cell gene expression data analysis. Genome Biol. *19*, 15. https://doi.org/10.1186/s13059-017-1382-0.
89. Stuart, T., Butler, A., Hoffman, P., Hafemeister, C., Papalexi, E., Mauck, W.M., 3rd, Hao, Y., Stoeckius, M., Smibert, P., and Satija, R. (2019). Comprehensive Integration of Single-Cell Data. Cell *177*, 1888–1902.e21. https://doi.org/10.1016/j.cell.2019.05.031.
90. Korsunsky, I., Millard, N., Fan, J., Slowikowski, K., Zhang, F., Wei, K., Baglaenko, Y., Brenner, M., Loh, P.R., and Raychaudhuri, S. (2019). Fast, sensitive and accurate integration of single-cell data with Harmony. Nat. Methods *16*, 1289–1296. https://doi.org/10.1038/s41592-019-0619-0.

CellPress
OPEN ACCESS

91. Traag, V.A., Waltman, L., and van Eck, N.J. (2019). From Louvain to Leiden: guaranteeing well-connected communities. Sci. Rep. *9*, 5233. https://doi.org/10.1038/s41598-019-41695-z.

92. Lun, A.T.L., McCarthy, D.J., and Marioni, J.C. (2016). A step-by-step workflow for low-level analysis of single-cell RNA-seq data with Bioconductor. F1000Res. *5*, 2122. https://doi.org/10.12688/f1000research.9501.2.

93. Subramanian, A., Tamayo, P., Mootha, V.K., Mukherjee, S., Ebert, B.L., Gillette, M.A., Paulovich, A., Pomeroy, S.L., Golub, T.R., Lander, E.S., and Mesirov, J.P. (2005). Gene set enrichment analysis: a knowledge-based approach for interpreting genome-wide expression profiles. Proc. Natl. Acad. Sci. USA *102*, 15545–15550. https://doi.org/10.1073/pnas.0506580102.

94. Bankhead, P., Loughrey, M.B., Fernández, J.A., Dombrowski, Y., McArt, D.G., Dunne, P.D., McQuaid, S., Gray, R.T., Murray, L.J., Coleman, H.G., et al. (2017). QuPath: Open source software for digital pathology image analysis. Sci. Rep. *7*, 16878. https://doi.org/10.1038/s41598-017-17204-5.

STAR★METHODS

KEY RESOURCES TABLE

REAGENT or RESOURCE	SOURCE	IDENTIFIER
**Biological samples**		
Healthy human lymph nodes	Cholecystectomy	REC 21/YH/0206
**Chemicals, peptides, and recombinant proteins**		
LMQ	Vaccine Formulation Initiative	https://doi.org/10.3389/fimmu.2022.976968
1,1' – Dioctadecyl-3,3,3′3′-Tetramethylindodicarbocyanine Perchlorate (DiD'/DiIC$_{18}$(5)) oil	ThermoFisher Scientific	Cat#:D307
TLR4 inhibitor, TAK242	Merck	Cat#: 614316
CP-456773 sodium salt (MCC950)	Sigma Aldrich	Cat#: PZ0280
**Critical commercial assays**		
LEGENDplex Human Inflammation Panel 1	BioLegend	Cat#:740809
Human IL-22 ELISA Kit – Quantikine	R&D Systems	Cat#: D2200
**Deposited data**		
Raw single cell RNAseq data	This paper	GEO: GSE294959
Processed single cell RNAseq data	This paper	CELLxGENE: LNCS: Atlas of healthy human lymph nodes with and without *ex vivo* adjuvant stimulation
**Software and algorithms**		
Original code for single cell RNAseq analysis	This paper	https://doi.org/10.5281/zenodo.15387998 (https://github.com/DendrouLab/LymphNodeCultureSlice_2025CellRep)
Original code for multiplex image quantification	This paper	https://doi.org/10.5281/zenodo.15396757 (https://github.com/edwardjenkins1/2D_spatial_analysis_lymph_node)
QuPath v0.4.3	Bankhead, P. et al. QuPath: Open source software for digital pathology image analysis. Scientific Reports (2017). https://doi.org/10.1038/s41598-017-17204-5	https://qupath.github.io/
FlowJo v10.8.1	BD Life Sciences	https://docs.flowjo.com/flowjo/getting-acquainted/10-8-release-notes/10-8-1-exhaustive-release-notes/
R: A language and environment for statistical	https://www.R-project.org/	Version 4.3.2

EXPERIMENTAL MODEL AND STUDY PARTICIPANT DETAILS

Human Participants

Cystic lymph nodes (LNs) were obtained from adult patients undergoing routine cholecystectomy, with LNs resected following gallbladder excision and processed directly after surgery. Patients had no history of cholecystitis as evidenced by no record of raised inflammatory markers and no thickening of the gallbladder wall by imaging. Clinically, all recruited patients recruited were diagnosed with symptomatic gallstones, a non-inflammatory condition distinct from cholecystitis. Patients with acute inflammatory pathology of the gallbladder, suspicion of malignancy, and diagnosis of pancreatitis or who had undergone endoscopic examination of the biliary tree in the preceding 6 months were excluded to ensure LNs were non-inflamed. In each case the assessment was made by a consultant hepatobiliary surgeon. Written informed consent was obtained from each patient, and samples were collected under REC 21/YH/0206 as approved by Yorkshire and The Humber - Sheffield Research Ethics Committee. Samples were kept anonymous and handled according to the ethical guidelines set by NHS Health Research Authority. Donor demographics are provided in Table S1. Human LN samples were collected over a 3-year period, with the aim of generating at least five donors per group to ensure

sufficient cell representation in transcriptomic analyses, resulting in seven donors for whole LN analyses and six for analysis of LN slices. Whole LNs were processed directly from unsliced LN or from the non-sliced portion of larger specimens. In all experiments full cross-sectional LN slices were successively assigned to either control or LMQ treated conditions, with an average of 4 slices per donor per condition (range 2–7 slices), to ensure fair representation across LN structures between groups.

Animals

Balb/c mice were purchased from Envigo (BALB/cOlaHsd) and maintained at the Centre for Human Genetics or the Kennedy Institute of Rheumatology, University of Oxford. Animals were housed under Specific Pathogen Free (SPF) conditions and in accordance with the recommendations of the UK Animals (Scientific Procedures) Act 1986 and ARRIVE guidelines. Protocols were approved by the University of Oxford Animal Care and Ethical Review Committee for use under Project License PP0984913 granted by the UK Home Office. Mice used in experiments were all female and 8-12 weeks old.

METHOD DETAILS

Materials, including adjuvant

Reagents used are listed in Table S6.

LMQ adjuvant was manufactured at the Vaccine Formulation Institute as described previously.[39] Briefly, liposomes composed of 1,2-dioleoyl-*sn*-glycero-3-phosphocholine (DOPC, Merck-Avanti, USA) and cholesterol were combined with QS-21 (Desert King International, USA) and synthetic TLR4 ligand 3D-6-acyl-PHAD (3D6AP) (Merck-Avanti, USA). LMQ was used *ex vivo* at a dose of 12.5μL contained 2.5μg of QS-21 and 1μg of 3D6AP. Fluorescent adjuvant was made by the addition of 1,1' – Dioctadecyl-3,3,3′3′-Tetramethylindodicarbocyanine Perchlorate (DiD'/DiIC_{18}(5)) oil (ThermoFisher, cat no. D307).

Precision lymph node slicing and culture

LNs were dissected from surrounding fat and underwent a 1 s wash in 0.01% Digitonin (Thermo Scientific), followed by 5–7 s in PBS, to remove residual lipid droplets as previously described,[32] prior to embedding as whole organs in 2.5% Agarose. Full organ cross-sectional slices were cut from embedded LNs using a Compresstome (Precisionary Instruments), following manufacturer's guidelines and with a stainless-steel blade set at a speed of 1–2 and oscillation of 6. Surrounding agarose was removed from cut slices and slices were rested in 1mL of complete media (RPMI +10% FCS + 1x Glutamine +50U/ml Pen/Strep +50μM β-mercaptoethanol + 1mM sodium pyruvate + 1x non-essential amino acids +20mM HEPES) for 1 h at 37°C before further culture in 1mL of fresh complete media. Each slice was cultured separately, and slices were successively assigned to either control or LMQ treated conditions, pooling an average of 4 slices per donor per condition (range 2–7 slices). For stimulations, 12.5μL of LMQ (Vaccine Formulation Institute) was added and slices cultured for 20 h. For inhibition experiments, slices were pre-treated with 1μM MCC950 (PZ0280, Sigma Aldrich) and/or 5μM TAK242 (614316, Merck) for 1 h before addition of LMQ.

Mouse experiments

For *in vivo* experiments mice were immunised intramuscularly with a total volume of 50μL containing 25μL of LMQ for cell recruitment studies, or fluorescent LMQ for adjuvant uptake experiments, in the tibialis muscle under light isoflurane anesthesia. Draining inguinal LNs were harvested at the indicated time points and digested in RPMI containing 0.8mg/ml Dispase, 0.2mg/ml Collagenase-P and 0.1mg/ml DNase, with mixing at 37°C for 3 × 20 min incubations. Cells were collected in cold PBS supplemented with 2% FCS and 2mM EDTA and filtered through a 100 μm cell strainer, before being stained for flow cytometric analysis. For cell recruitment studies, precision count beads (BioLegend) were included for calculation of absolute numbers of neutrophils ($CD45^{+}CD3^{-}B220^{-}Ly6C^{+}Ly6G^{++}$), monocytes ($CD45^{+}CD3^{-}B220^{-}Ly6C^{++}Ly6G^{+}$) and dendritic cells ($CD45^{+}CD3^{-}B220^{-}Ly6C^{-}Ly6G^{-}CD11c^{+}MHCII^{+}$).

For *ex vivo* adjuvant uptake experiments, inguinal LNs were obtained and cut into 300 μm thick slices and cultured with fluorescent LMQ for the indicated timepoints. After culture, slices were digested and stained for flow cytometric analysis.

Single cell RNA sequencing

Cell and library preparation for single-cell mRNA sequencing

Human LNs were mechanically disrupted with scissors before being enzymatically digested in RPMI containing 0.8mg/ml Dispase, 0.2mg/ml Collagenase-P and 0.1mg/ml DNase, with mixing. This was performed at 37°C for 3 × 20 min incubations for whole LN preparations, and 2 × 15 min for slice preparations. Slices from treatment groups were pooled. After digestion cells were collected in cold PBS supplemented with 2% FCS and 2mM EDTA and filtered through a 100 μm cell strainer. Cells were stained with live/dead near infra-red (nIR), anti-CD3, anti-CD20, anti-CD235a, and 7AAD added directly before sorting with the SONY SH800S cell sorter. Dead cells and red blood cells were removed by sorting ($CD235a^{-}7AAD^{-}Live/Dead^{-}$), while T ($CD3^{+}CD20^{-}$) and B ($CD3^{-}CD20^{+}$) cells were sorted separately and spiked back at ratio of 1:20 of sorted non-T/B ($CD3^{-}CD20^{-}$) cells. Antibody details are listed in Table S7.

The cell count and viability were determined with acridine orange/propidium iodide fluorescence using a LUNA-FX7 cell counter (Logos Biosystems). Approximately 20,000 cells per sample were loaded onto the 10X Genomics Chromium Controller (Chip G). Gene expression sequencing libraries were prepared using the 10X Genomics Single Cell 3′ Reagent Kits v3.1 following the

manufacturer user's guide (CG000330). The final libraries were loaded on the NovaSeq6000 sequencing platform (Illumina, v1.5 chemistry, 150bp paired end).

Sequencing and data processing

Expression libraries were sequenced by the Oxford Genomics Center or Novogene on the NovaSeq6000 S4. Each sample was sequenced to an average depth of approximately 36,000 reads per cell.

Cell Ranger v7.0.0 count, with include intron parameter set to TRUE, was used to align reads to the GRCh38-2020-A human transcriptome and generate feature-barcode matrices from the Chromium single-cell RNA-sequencing output. Panpipes was used to perform quality control, batch correction, and clustering pipelines.[87] Quality control for high-quality single cells include removing cells expressing fewer than log1p(6) genes, and cells with mitochondria gene count percentage greater than 30.

Highly variable gene selection, dimension reduction, clustering, and annotation

UMI counts were normalised by the total number of UMIs per cell, and converted to transcripts-per-10000, and then log-normalised.[88] Top 2000 highly variable genes were selected using vst method from Seurat[89] and implemented in Scanpy.[88] T cell receptor and immunoglobulin genes were removed from highly variable gene lists. Data was then scaled prior to PCA. Harmony was used for batch correction by donor.[90] Leiden clustering was applied to determine cell populations.[91] Further sub-clustering on myeloid populations was required to derive the following populations: Monocytes/Macrophages, DC, Neutrophils/Granulocytes, and on Stromal/Endothelial cells to derive: Fibroblastic Reticular cells, Follicular Dendritic cells, Pericytes, Blood Endothelial cells and Lymphatic Endothelial cells.

Comparison of whole vs. slice transcriptomes

Median gene expression was determined for each gene across cell clusters in whole and control slice samples. Genes were then filtered to the union of the top 2000 most variable features across both datasets. The Pearson correlation coefficients were calculated between each cluster pair from whole and control slice datasets and visualised by a heatmap, with correlation coefficients >0.8 indicated with an asterix.

Differentially expressed gene analysis and gene set enrichment analysis

Differential gene expression (DEG) was determined by Welch t-tests between LMQ and control slice samples using pairwiseTTests in scran[92] after blocking on cell type. DEG lists are detailed in Table S3. EnhancedVolcano package was used to summarise DEG results as a volcano plot.

For gene set enrichment analysis, differentially expressed genes with a p value threshold of 0.05 were selected to create a ranked gene list. Enrichment of canonical pathways gene sets (c2.cp.v2023.2.Hs.symbols.gmt) and ontology gene sets (c5.go.bp.v2023.2.Hs.symbols.gmt) from within the Human MSigDB collection were then tested by Gene Set Enrichment Analysis (GSEA) using the fgsea package (https://github.com/ctlab/fgsea/)[93] against ranked gene lists for each cell type. Gene sets were filtered with a p value of 0.05, minimum size of 15, maximum size of 400 and number of permutations set at 10,000. Enriched gene lists and leading edge genes referred to in the text are listed in Table S4.

Differential abundance analysis

T and B cells were selectively depleted during transcriptomic analysis, so were excluded from differential abundance analyses. Differential abundance was determined by performing an ANOVA test where the overall error sum of squares and degrees of freedom are calculated from a linear model of cell type and sample type (additive model).

Further packages used

Other packages used not mentioned already include ggplot2, dittoSeq, and single cell experiment, conducted in R (version 4.3.2, https://www.R-project.org/).

Multiplexed fluorescence imaging

5μm formalin-fixed paraffin embedded (FFPE) LN slides were deparaffinised and rehydrated. Slides were then permeabilized for 10 min in 0.3% Triton X-100 and washed in PBS. Antigen retrieval was performed using the NxGen decloaking chamber (Biocare Medical, Pacheco, CA, USA) for 20 min each in boiling pH6 Citrate (Agilent, S1699) and pH9 Tris-based antigen retrieval solutions. Slides were then blocked for 1 h at room temperature in PBS with 3% BSA (Merck, A7906) and 10% Donkey serum (Bio-Rad, C06SB) containing Human TruStain FcX Fc Receptor Blocking Solution (BioLegend). Slides were then washed and stained with DAPI (Thermo, D3571) for 15 min, before being washed in PBS and coverslipped with mounting media combining 50% glycerol (Sigma, G5516) and 4% propyl gallate (Sigma, 2370). The GE Cell DIVE system was used to image at 20X through iterative rounds of bleaching and staining, with slides de-coverslipped in PBS between each round. Background imaging was performed initially and after each bleaching round, and used to subtract background fluorescence from subsequent stained images. Staining was performed with three antibodies at a time, in blocking buffer (PBS, 3% BSA, 10% donkey serum). The intial round used primary antibodies incubated overnight at 4°C, followed by washes in PBS and 0.05% Tween 20, and then secondary antibodies stained for 1 h at room temperature. All subsequent rounds used directly conjugated antibodies stained either overnight at 4°C or for 1 h at room temperature. Where required, BSA-Azide-free antibodies were manually conjugated using Mix-*n*-Stain Antibody Labeling Kits (Biotium) following manufacturer's protocols. Bleaching was performed using $NaHCO_3$ (0.1M, pH 11.2; Sigma S6297) and 3% H_2O_2 (Merck, 216763) for three rounds of 15 min, with PBS washes in between each. DAPI staining was repeated after each bleach round. The antibodies, reagents, instruments and software used are listed in Table S8. Images were analyzed with QuPath v0.4.3.[94]

Supernatant analysis by LEGENDPlex and ELISA

At the end of culture, supernatants were collected from individual slice cultures, centrifuged to pellet contaminating cells and the supernatant transferred and stored at −80°C. Thawed supernatants were analyzed for released cytokines by the LEGENDplex Human Inflammation Panel 1 (BioLegend, 740809) following the manufacturer's instructions. Data were collected on an Aurora spectral cytometer (Cytek Biosciences) and analyzed by LEGENDplex Data Analysis Software Suite. IL-22 cytokine concentrations were quantified by Human IL-22 Quantikine ELISA Kit (R&D Systems, D2200) following manufacturer's instructions. Cytokine concentrations were averaged from 2 to 4 slices per condition for each donor, and are detailed in Table S2. Adjuvant-mediated stimulation was assessed by Wilcoxon matched-pairs signed rank test and the effect of inhibitor addition was analyzed by mixed effects analysis with Dunnett's multiple comparisons to LMQ alone.

Flow cytometry

Single cell suspensions were blocked in PBS with 2% FCS containing Human TruStain FcX Fc Receptor Blocking Solution (BioLegend) followed by staining with Live/Dead Fixable dye (ThermoFisher Scientific) and antibodies (detailed in Table S7) in PBS with 2% FCS for 20 min at 4°C. Cells were washed in the same buffer and then fixed in Fixation Buffer (BioLegend) for 15 min at room temperature, before being washed and resuspended in the same buffer for analysis. Where intracellular staining was required, cells were fixed, permeabilized and intracellular markers stained using eBioscience Foxp3/Transcription Factor Staining Buffer Set (ThermoFisher Scientific), according to the manufacturer's instructions. Data was collected on either an LSRFortessa X20 (BD) or an Aurora spectral cytometer (Cytek Biosciences) and analyzed by FlowJo v10.8.1 (BD Life Sciences). The antibodies, reagents, instruments, and software used are listed in Table S7.

QUANTIFICATION AND STATISTICAL ANALYSIS

Statistical analysis was performed in GraphPad Prism v10 (GraphPad Software, LLC) or R version 4.3.2 (R Core Team (2023). R: A Language and Environment for Statistical Computing, R Foundation for Statistical Computing, Vienna, Austria. https://www.R-project.org/). The statistical tests used, definitions of significance and number of biological replicates, where *n* represents the number of donors, are detailed in figure legends.

Cell Reports, Volume *44*

Supplemental information

Ex vivo model of functioning human lymph node reveals role for innate lymphocytes and stroma in response to vaccine adjuvant

Joannah R. Fergusson, Jacqueline H.Y. Siu, Nitya Gupta, Edward Jenkins, Eloise Nee, Sören Reinke, Tamara Ströbel, Ananya Bhalla, Shyami M. Kandage, Thomas Courant, Sarah Hill, Moustafa Attar, Michael L. Dustin, Alex Gordon-Weeks, Mark Coles, Calliope A. Dendrou, and Anita Milicic

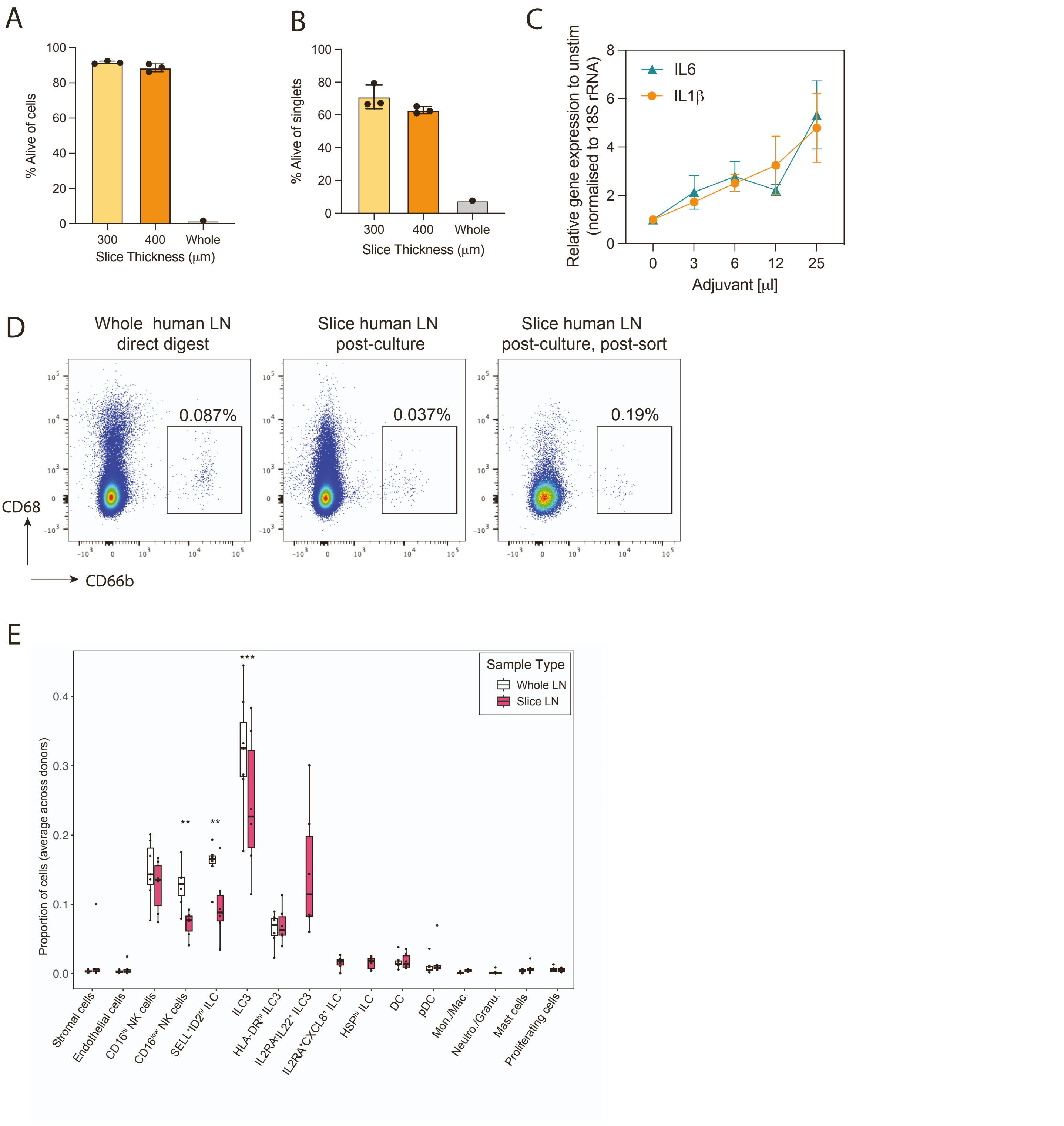

Figure S1: Viability and responsiveness of LN slices in culture. Related to Figure 1.
(A) Percentage of live cells (L/D negative amongst cells gated on FSC vs SSC) from pig LN slices of indicated thickness compared to cultured whole LN after 20 h culture. (n=3 for slices, n=1 whole LN). **(B)** Percentage of live cells (L/D negative amongst all events, gated on singlets only) from pig LN slices of indicated thickness compared to cultured whole LN after 20 h culture (n=3 for slices, n=1 whole LN). **(C)** Expression of IL6 and IL1β relative to housekeeping 18S rRNA in pig LN slices stimulated with the indicated volumes of adjuvant in 1ml of media after 20 h of culture (n=3, average of duplicate wells). **(D)** Presence of neutrophils (gated on singlets; live cells; CD45+CD66b+) in single cell suspensions from human whole LN or sliced LN after 20 h culture pre- (middle) or post- sorting (right). Percentage of CD66b+ in CD45+ cells is indicated above each gate (representative of 3 donors). **(E)** Proportion of each cell type (excluding T and B cells) in single cell transcriptomic analyses of human whole LN (black outline) and LN slices (magenta), each point represents an individual donor. **$p \leq 0.005$, ***$p < 0.0005$ by ANOVA using a linear model between cell types with matched clusters in whole and slice datasets.

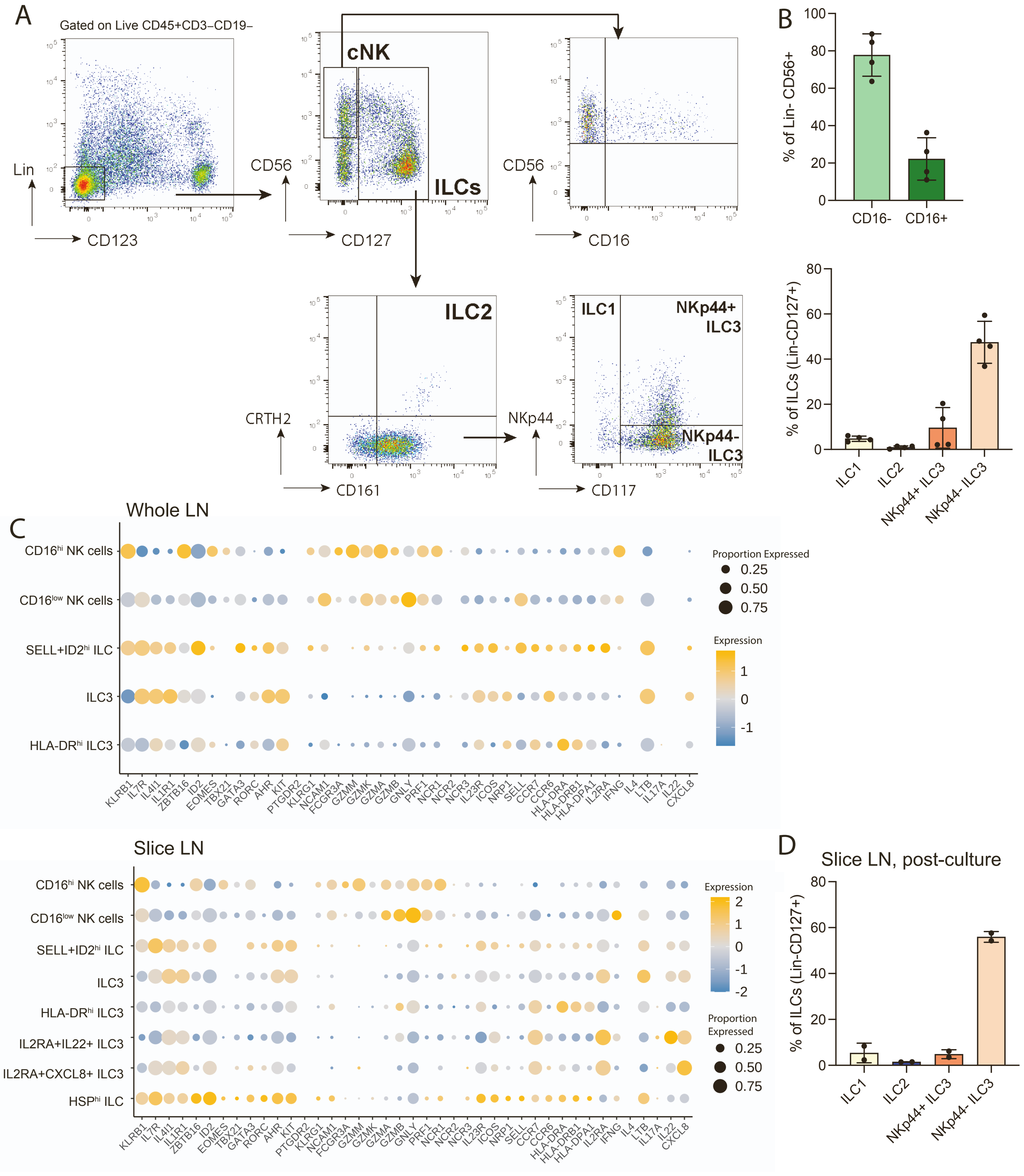

Figure S2: ILC populations of the human cystic LN. Related to Figure 1.
(A) Gating strategy for the identification of NK cells and ILC subsets from whole human LN by flow cytometry. Lin = CD14, CD66, CD34. **(B)** Quantification of the percentage of NK cells either CD16− or CD16+ (above) and ILC subsets (below), as gated in A. **(C)** Dot plots showing expression of marker genes for each ILC cluster within whole (above) and slice (below) human LN. Colour indicates relative log-normalised level of expression across clusters and dot size the proportion of each cluster expressing each gene. **(D)** Quantification of the percentage of ILC subsets in slice human LN following 20 h culture by flow cytometry, as in B.

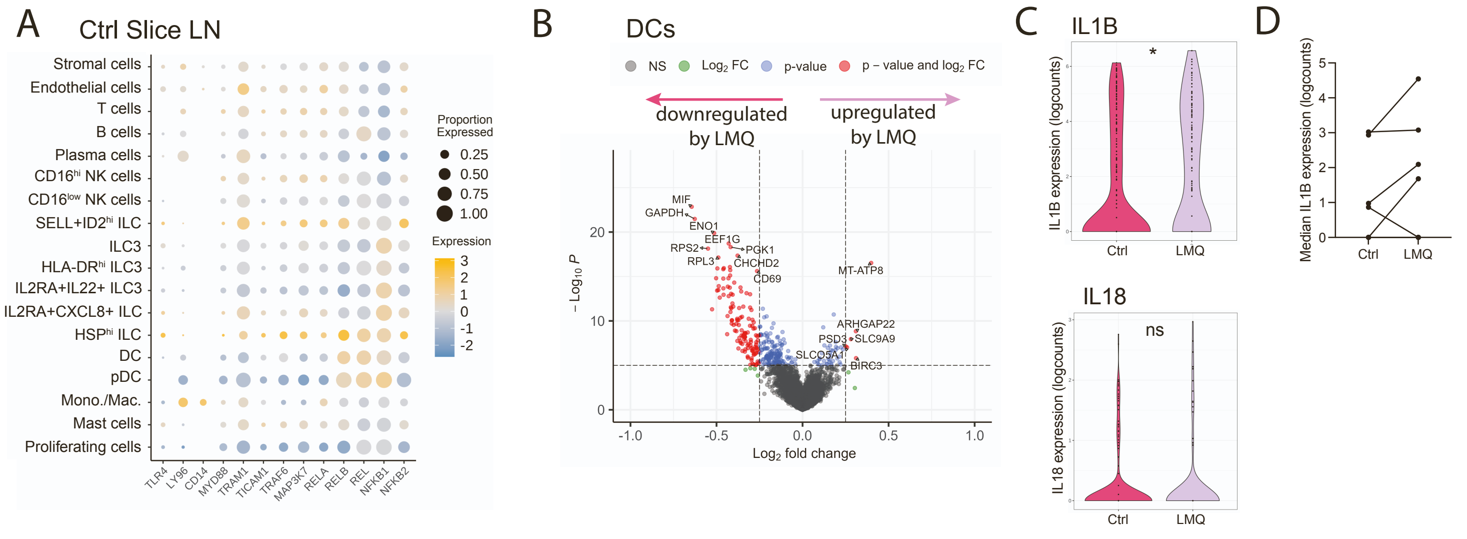

Figure S3: LN slice responses to TLR4 and inflammasome activation by adjuvant LMQ. Related to Figure 3. **(A)** Dot plot showing expression of TLR4 signalling components for each cell cluster within untreated (Ctrl) LN slices. Colour indicates relative log-normalised level of expression across clusters and dot size the proportion of each cluster expressing each gene. **(B)** Volcano plot indicating differentially expressed genes between LMQ-stimulated (right: upregulated) and control (left: upregulated) conditions in DCs. Genes with $\log_2$ fold-change >0.25 are indicated in green, those with p value $<10^{-5}$ in blue and those fulfilling both criteria in red. **(C)** Log normalised expression levels of IL1B and IL18 transcripts in the Monocytes/Macrophages cluster of all donors in control (magenta) and LMQ (lavender) treated samples. $^*p<0.05$. **(D)** Median log normalised expression levels of IL1B transcripts in the Monocytes/Macrophages cluster of paired donor slice conditions, either control or LMQ treated conditions.

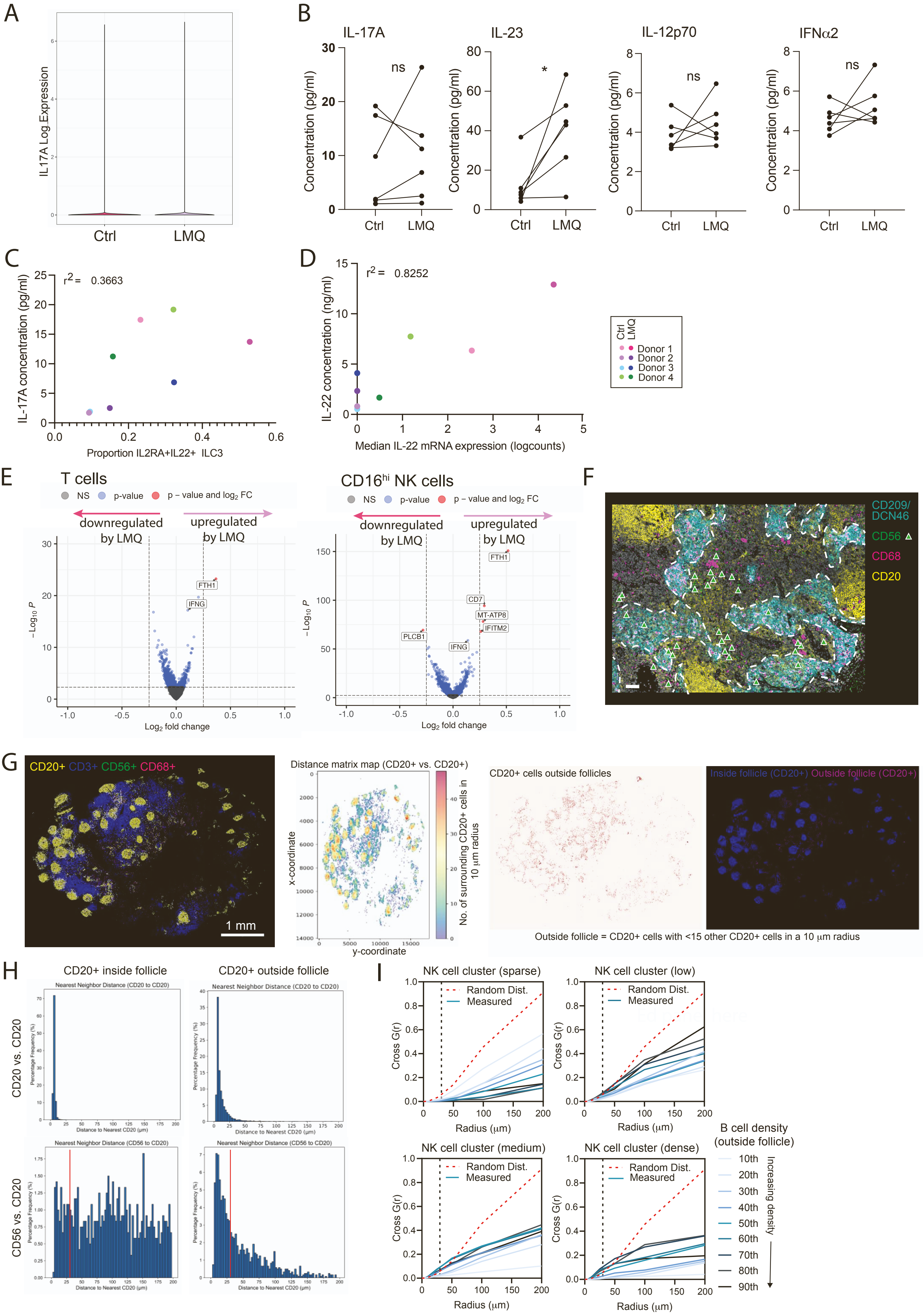
A
IL17A Log Expression
Ctrl
LMQ
B
IL-17A
IL-23
IL-12p70
IFNα2
Concentration (pg/ml)
ns
*
ns
ns
Ctrl
LMQ
C
r2 = 0.3663
IL-17A concentration (pg/ml)
Proportion IL2RA+IL22+ ILC3
D
r2 = 0.8252
IL-22 concentration (ng/ml)
Median IL-22 mRNA expression (logcounts)
Ctrl
LMQ
Donor 1
Donor 2
Donor 3
Donor 4
E
T cells
NS
p-value
p – value and log2 FC
downregulated by LMQ
upregulated by LMQ
FTH1
IFNG
Log2 fold change
CD16hi NK cells
CD7
MT-ATP8
IFITM2
PLCB1
F
CD209/ DCN46
CD56
CD68
CD20
G
CD20+ CD3+ CD56+ CD68+
1 mm
Distance matrix map (CD20+ vs. CD20+)
x-coordinate
y-coordinate
No. of surrounding CD20+ cells in 10 μm radius
CD20+ cells outside follicles
Inside follicle (CD20+) Outside follicle (CD20+)
Outside follicle = CD20+ cells with <15 other CD20+ cells in a 10 μm radius
H
CD20+ inside follicle
CD20+ outside follicle
CD20 vs. CD20
CD56 vs. CD20
Nearest Neighbor Distance (CD20 to CD20)
Nearest Neighbor Distance (CD56 to CD20)
Percentage Frequency (%)
Distance to Nearest CD20 (μm)
I
NK cell cluster (sparse)
NK cell cluster (low)
NK cell cluster (medium)
NK cell cluster (dense)
Random Dist.
Measured
Cross G(r)
Radius (μm)
B cell density (outside follicle)
10th
20th
30th
40th
50th
60th
70th
80th
90th
Increasing density

Figure S4: Activation of ILCs by adjuvant and downstream signaling to B cells. Related to Figure 4.
(A) Log normalised expression levels of IL17A transcripts in all donors in control (magenta) and LMQ (lavender) treated samples. **(B)** Concentrations of IL-17A, IL-23, IL-12p70 and IFNα2 in slice culture supernatants after 20 h culture without (Ctrl) or with LMQ adjuvant (LMQ) from paired donor conditions (n=6, each point is the average of 2-4 slices from one donor *p<0.05 or ns = not significant by Wilcoxon matched-pairs signed rank test). **(C)** Proportion of IL2RA+IL22+ ILC3 cells per donor and per condition against IL-17A concentration detected in culture supernatants. Dots are coloured according to donor, and shade according to treatment (light = control, dark = LMQ-treated). R^2 value was calculated by simple linear regression. **(D)** Median log-normalised expression of IL-22 in the IL2RA+IL22+ ILC3 cluster per donor and per condition against IL-22 concentration detected in culture supernatants. Dots are coloured according to donor, and shade according to treatment (light = control, dark = LMQ-treated). R^2 value was calculated by simple linear regression. **(E)** Volcano plot indicating differentially expressed genes between LMQ-stimulated (right: upregulated) and control (left: upregulated) conditions in T cells (left) and CD16^{hi} NK cells (right). Genes with $\log_2$ fold-change >0.25 are indicated in green, those with p value $<10^{-5}$ in blue and those fulfilling both criteria in red. **(F)** Multiplexed stained image of whole human LN for NK cells (CD56, green triangles) in relation to medullary sinuses (CD209/DCN46; dashed line). Macrophages (CD68) and B cells (CD20) are also shown; scale bars are 50 μm. Images are representative of three individuals. **(G)** (Left) Image of segmented nuclei coloured by indicated marker. Scale bar is indicated. (Middle) Distance map of CD20+ cells from segmented image left, coloured based on the number of surrounding CD20+ cells within a 10 μm radius. Red and blue regions demarcate dense and sparse areas of CD20+ cells, respectively. (Right) CD20+ cells with <15 other CD20+ cells within a 10 μm radius were considered outside follicles. (Right) Nuclei coloured according to this threshold, with blue and magenta nuclei indicating CD20+ inside and outside follicles, respectively. **(H)** Nearest-neighbourhood analysis of CD20+ cells inside and outside follicles vs. either other CD20+ cells (inside follicles) or vs. CD56+ cells (NK cells). Red line indicates 30 μm distance. **(I)** Cross-G analysis of NK and CD20+ cells (outside follicles) after stratifying NK and B cells into groups based on their local density. NK cells were stratified into four distinct cluster sizes (‘sparse’, ‘low’, ‘medium’ and ‘dense’ based on probability density quartiles) and B cells into ten (one being sparse, and ten being dense). Red-dashed line is the theoretical likelihood of a cell cluster being nearby for a given radius from the cell of interest. This is based on a Poisson distribution and uses the measured area of the LN (see Methods). The teal line indicates the empirical value from the image. Empirical > theoretical = cells examined are closer than you would expect by random. Empirical < theoretical = cells further apart than expected by random. Empirical = theoretical = randomly distributed. This highlights whether sparse or dense NK/B cells tended to congregate more than expected by random. Figure shows that only dense NK and B cell populations tended to congregate closer than expected by random. Analysis is based on using distance matrices from 33,082 CD20+ total, 24,785 CD20+ inside follicles, 8297 CD20+ outside follicles and 1201 CD56+ cells total. 300 cells were used per NK quartile (i.e., sparse, low, medium, dense). ~830 cells were used per CD20+/B cell decile.

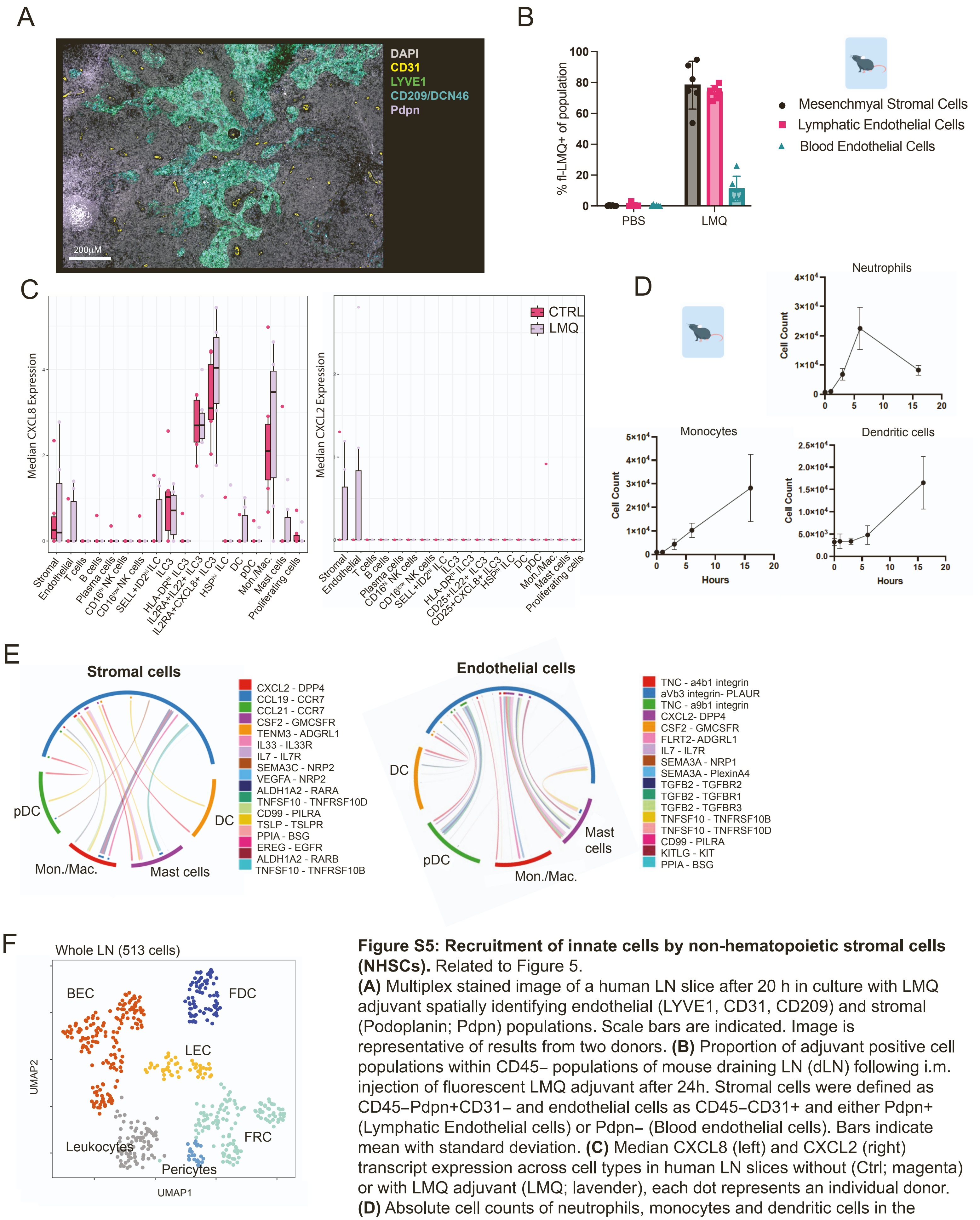

Figure S5: Recruitment of innate cells by non-hematopoietic stromal cells (NHSCs). Related to Figure 5.
(A) Multiplex stained image of a human LN slice after 20 h in culture with LMQ adjuvant spatially identifying endothelial (LYVE1, CD31, CD209) and stromal (Podoplanin; Pdpn) populations. Scale bars are indicated. Image is representative of results from two donors. **(B)** Proportion of adjuvant positive cell populations within CD45– populations of mouse draining LN (dLN) following i.m. injection of fluorescent LMQ adjuvant after 24h. Stromal cells were defined as CD45–Pdpn+CD31– and endothelial cells as CD45–CD31+ and either Pdpn+ (Lymphatic Endothelial cells) or Pdpn– (Blood endothelial cells). Bars indicate mean with standard deviation. **(C)** Median CXCL8 (left) and CXCL2 (right) transcript expression across cell types in human LN slices without (Ctrl; magenta) or with LMQ adjuvant (LMQ; lavender), each dot represents an individual donor. **(D)** Absolute cell counts of neutrophils, monocytes and dendritic cells in the mouse dLN at various timepoints following i.m. injection of LMQ adjuvant (n=6). **(E)** Relevant interactions upregulated in response to adjuvant stimulation between stromal (left) and endothelial (right) cells and indicated innate cell types as calculated by CellPhoneDB. Arrows in the chord diagram indicate the direction of the interaction, with coloured bars indicating the sender cell type and the colour of the bar the receiver cell type. **(F)** UMAP plot of NHSCs from whole human LN coloured by cluster and stromal cell type.

Table S1: Human LN donor demographics

Donor	Self-declared Ethnicity	Age	Sex
1	White British	20	Female
2	White British	61	Female
3	White Eastern European	45	Male
4	White British	59	Male
5	White British	49	Female
6	White Eastern European	40	Male
7	White British	64	Female
8	White British	54	Female
9	White British	54	Female
10	Asian	51	Female
11	White other	75	Female

Table S6: General reagents used throughout study

REAGENT	SOURCE	IDENTIFIER
LMQ	Vaccine Formulation Initiative	DOI: 10.3389/fimmu.2022.976968
RPMI 1640 Medium	ThermoFisher Scientific (Gibco)	Cat#: 21875-034
Dispase II, powder	ThermoFisher Scientific	Cat#: 17105-041
Collagenase P	Merck	Cat#: 11213857001
Deoxyribonuclease I from bovine pancreas	Sigma Aldrich	Cat#: D5025-150KU
PBS, pH 7.4	ThermoFisher Scientific (Gibco)	Cat#: 10010015
Fetal Bovine Serum, Value, One Shot™ format	ThermoFisher Scientific (Gibco)	Cat#: A5209401
UltraPure 0.5M EDTA, pH 8.0	ThermoFisher Scientific (Invitrogen)	Cat#: 15575020
1,1' – Dioctadecyl-3,3,3'3'-Tetramethylindodicarbocyanine Perchlorate (DiD'/DiIC$_{18}$(5)) oil	ThermoFisher Scientific	Cat#:D307
Digitonin (5%)	ThermoFisher Scientific	Cat#: BN2006
UltraPure Agarose	ThermoFisher Scientific (Invitrogen)	Cat#: 16500500
Penicillin-Streptomycin (10,000 U/ml)	ThermoFisher Scientific (Gibco)	Cat#: 15140122
2-Mercaptoethanol	VWR Lifescience	Cat#: 0482-100ML
Sodium Pyruvate (100mM)	ThermoFisher Scientific	Cat#: 11360070
MEM Non-Essential Amino Acids Solution (100X)	ThermoFisher Scientific	Cat#: 11140035
HEPES (1M)	ThermoFisher Scientific (Gibco)	Cat#: 15630056
CP-456773 sodium salt (MCC950)	Sigma Aldrich	Cat#: PZ0280
TLR4 inhibitor, TAK242	Merck	Cat#: 614316
LEGENDplex Human Inflammation Panel 1	BioLegend	Cat#: 740809
Human IL-22 ELISA Kit - Quantikine	R&D Systems	Cat#: D2200
eBioscience Cell Stimulation Cocktail (500X)	ThermoFisher Scientific (Invitrogen)	Cat#: 00-4970-93

Table S7: Flow cytometry Antibodies, Instrument, Software

REAGENT	SOURCE	IDENTIFIER	
**Antibodies**		**Clone**	**Cat. No.**
Brilliant Violet 795 anti-mouse CD45 Antibody	BioLegend	30-F11	103149
CD45R (B220) Monoclonal Antibody, Alexa Fluor 488	eBioscience	RA3-6B2	53-0452-82
Alexa Fluor 594 anti-mouse F4/80 Antibody	BioLegend	BM8	123140
PE/Cyanine7 anti-mouse CD169 (Siglec-1) Antibody	BioLegend	3D6.112	142411
Ly6G/Ly-6C Monoclonal Antibody, eFluor450, eBiosience	ThermoFisher Scientific	RB6-8C5	48-5931-82
Brilliant Violet 605 anti-mouse CD11c Antibody	BioLegend	N418	117334
Brillaint Violet 711 anti-mouse/human CD11b Antibody	BioLegend	M1/70	101241
Alexa Fluor 488 anti-mouse CD3 Antibody	BioLegend	17A2	100212
CD19 Monoclonal Antibody, FITC, eBioscience	ThermoFisher Scientific	MB19-1	11-0191-85
Alexa Fluor 488 anti-mouse NK1.1 Antibody	BioLegend	PK136	108718
PerCP/Cyanine5.5 anti-mouse I-A/I-E Antibody	BioLegend	M5/114.15.2	107626
PE anti-mouse Ly-6C Antibody	BioLegend	HK1.4	128008
BD Horizon BUV395 Mouse Anti-Human CD3	BD Biosciences	UCHT1	563548
BD Horizon BUV496 Mouse Anti-Human CD45	BD Biosciences	HI30	750179
Brillaint Violet 421 anti-human CD19 Antibody	BioLegend	HIB19	302233
BD OptiBuild BV750 Mouse Anti-Human CD25 (IL-2 Receptor α)	BD Biosciences	2A3	747290
BD OptiBuild BV786 Mouse Anti-Human CD161	BD Biosciences	HP-3G10	748281
PerCP/Cyanine5.5 anti-human CD14 Antibody	BioLegend	HCD14	325621
Alexa Fluor 488 anti-human CD56 (NCAM) Antibody	BioLegend	HCD56	318311
PE anti-human CD117 (c-kit) Antibody	BioLegend	104D2	313203
PE/Dazzle 594 anti-human CD294 (CRTH2) Antibody	BioLegend	BM16	350125
PE/Cyanine5 anti-human CD16 Antibody	BioLegend	3G8	302009
PE/Cyanine7 anti-human CD123 Antibody	BioLegend	6H6	306009
Alexa Fluor 647 anti-human CD336 (NKp44) Antibody	BioLegend	P44-8	325112
Alexa Fluor 700 anti-human CD66b Antibody	BioLegend	G10F5	305113
Alexa Fluor 700 anti-human CD34 Antibody	BioLegend	561	343621
APC/Fire 810 anti-human CD127 (IL-7Rα) Antibody	BioLegend	A019D5	351374
Alexa Fluor 700 anti-human CD16 Antibody	BioLegend	3G8	302026
PE/Cyanine7 anti-human CD11c Antibody	BioLegend	S-HCL-3	371508
PE anti-human Podoplanin Antibody	BioLegend	NZ-08	337003
Alexa Fluor 488 anti-human CD3 Antibody	BioLegend	HIT3a	300319
Alexa Fluor 488 anti-human CD19 Antibody	BioLegend	HIB19	302219
Brilliant Violet 785 anti-human CD68 Antibody	BioLegend	Y1/82A	333826
BD OptiBuild BV650 Anti-Human CD31	BD Biosciences	M89d3	744465
Brilliant Violet 605 anti-human CD45 Antibody	BioLegend	HI30	304042
PE anti-human CD235a (Glycophorin A) Antibody	BioLegend	I264	349105
BD Horizon BV421 Mouse Anti-Human HLA-DR	BD Biosciences	G46-6	562805
**Other**			
Live/Dead Fixable Near-IR Dead Cell Kit	ThermoFisher Scientific	Cat#: L10119	
Live/Dead Fixable Aqua Dead Cell Kit	ThermoFisher Scientific	Cat#: L34957	
7-AAD Viability Staining Solution	BioLegend	Cat#: 420403	
Human TruStain FcX Fc Receptor Blocking Solution	BioLegend	Cat#: 422302	
TruStain FcX (anti-mouse CD16/32) Antibody	BioLegend	Cat#: 101320	
ArC Amine Reactive Compensation Bead Kit	ThermoFisher Scientific	Cat#: A10628	
AbC Total Antibody Compensation Kit	ThermoFisher Scientific	Cat#: A10497	
BD CompBeads Anti-Mouse Ig, k/Negative Control Compensation Particles Set	BD Biosciences	Cat#: 552843	
Precision Count Beads	BioLegend	Cat#: 424902	
Fixation Buffer	BioLegend	Cat#: 420801	
eBioscience Foxp3/Transcription Factor Staining Buffer Set	ThermoFisher Scientific	Cat#: 00-5523-00	
**Software**			
FlowJo v10.8.1	BD Life Sciences	https://docs.flowjo.com/flowjo/getting-acquainted/10-8-release-notes/10-8-1-exhaustive-release-notes/	
**Hardware**			
LSRFortessa X20 (R/B/V/YG)	BD Biosciences		
Cytek Aurora (R/B/V/YG/UV)	Cytek Biosciences		

Table S8: CellDIVE Antibodies, Instrument, Software

REAGENT	SOURCE	IDENTIFIER	
**Antibodies**		**Clone**	**Cat. No.**
Recombinant Anti-NCAM1 Antibody	Abcam	EPR2566	ab133345
IL3RA/CD123 Mouse mAb	Cell Signaling Technology	BR4MS	23797S
Purified anti-human IgD Antibody	BioLegend	W18340A	324502
Mast Cell Tryptase Antibody	Santa Cruz	AA1	sc-59587
Recombinant Alexa Fluor 555 Anti-CD68 Antibody	Abcam	EPR20545	ab280860
Alexa Fluor 647 anti-human CD66b Antibody	BioLegend	G10F5	305110
Alexa Fluor 647 Anti-Sialoadhesin/CD169 antibody	Abcam	SP216	ab306568
Alexa Fluor 488 Anti-CD11c Antibody	Abcam	EP1347Y	ab275738
Alexa Fluor 555 Anti-CD3 Antibody	Abcam	SP162	ab307335
CD31/PECAM-1 Antibody	Novus Biologicals	C31.3	NBP2-15202
Alexa Fluor 488 Anti-CD20 Antibody	Abcam	EP459Y	ab198941
Purified Mouse Anti-Human CD209	BD Biosiences	DCN46	551186
Podoplanin Mouse Monoclonal Antibody	Cell Marque	D2-40	322M
Recombinant Anti-CD14 Antibody	Abcam	EPR3653	ab133335
Recombinant Anti-LYVE1 Antibody	Abcam	EPR21857	ab232935
Donkey Anti-Rabbit IgG H&L	Abcam	Polyclonal	ab150073
Donkey Anti-Mouse IgG H&L	Abcam	Polyclonal	ab150106
Donkey Anti-Rat IgG H&L	Abcam	Polyclonal	ab150155
**Other**			
Bovine Serum Albumin (BSA)	Sigma – Aldrich	Cat#: A7906	
Citrate Antigen Retrieval Solution (ARS1)	Vector Laboratories	Cat#: H-3300	
DAPI Stock Solution	ThermoFisher Scientific	Cat#: D3571	
Donkey Serum	Sigma – Aldrich	Ca#: D9663	
EDTA	Alfa Aesar	Cat#: 33312	
Ethanol	Honeywell	Cat#: 32221-2.5L	
FcR Blocking Reagent	Miltenyi	Cat#: 130-059-901	
Human TruStain FcX Fc Receptor Blocking Solution	BioLegend	Cat#: 422302	
Glycerol	Sigma – Aldrich	Cat#: G5516	
Hydrogen Peroxide	Sigma – Aldrich	Cat#: 216763	
Phosphate Buffered Saline 10X	VWR	Cat#: 437117K	
Propyl Gallate	Sigma – Aldrich	Cat#: 02370	
Sodium Bicarbonate	Sigma - Aldrich	Cat #: S6297	
Sodium Hydroxide Pellets	Sigma – Aldrich	Cat#: 30620-1KG-M	
Triton X-100	Sigma – Aldrich	Cat#: T9284	
Trizma®Base	Sigma – Aldrich	Cat#: T6066	
Xylene	Sigma – Aldrich	Cat#: 534056	
Super Frost Plus Glass Slides	Fisher Scientific	Product code 10149870	
Leica SurgiPath Coverglass 24 x 50 mm coverslips (Size 1)	Leica	Cat#: 3800145G	
Mix-n-Stain CF450 Dye Antibody Labeling Kit (50-110ug)	Biotium	Cat#: 92324	
Mix-n-Stain CF555 Dye Antibody Labeling Kit (50-110ug)	Biotium	Cat#: 92234	
**Software**			
QuPath v0.4.3	Bankhead, P. et al. QuPath: Open source software for digital pathology image analysis. Scientific Reports (2017). https://doi.org/10.1038/s41598-017-17204-5	https://qupath.github.io/	
**Hardware**			
Cell DIVE multiplexed imaging platform	General Electric	https://www.ge.com/research/project/multiplexed-tissue-imaging-platform	
Epredia Gemini AS Automated Slide Stainer	Fisher Scientific	https://www.fishersci.co.uk/shop/products/gemini-as-automated-slide-stainer/15243973	
Manual Rotary Microtome HistoCore BIOCUT	Leica	https://www.leicabiosystems.com/en-gb/histology-equipment/microtomes/histocore-biocut/	

Table S9: qPCR primers and reagents

Gene symbol	Gene	Forward primer	Reverse primer	Manufacturer
18S rRNA	18S ribosomal RNA	GACAAATCGCTCCACCAACT	CCTGGGGCTTAATTTGACTC	ThermoFisher Scientific
IL1B	Interleukin 1b	TGATGGCTAACTACGGTGACAACA	CTCAGAGAACCAAGGTCCAGGTTT	
IL6	Interleukin 6	CTCTCCACAAGCGCCTTTAGTC	GCATTTTGTCTGAGGTGGCATC	

REAGENT	SOURCE	IDENTIFIER
SYBR Green PCR Master Mix	ThermoFisher Scientific	Cat#: 4309155
First Strand cDNA Synthesis Kit	ThermoFisher Scientific	Cat#: K1612
RNeasy Mini Kit	Qiagen	Cat#: 74106
RNase-Free DNase Set	Qiagen	Cat#: 79254
**SOFTWARE**		
Design & Analysis Software 2.6.0	AppliedBioSystems by ThermoFisher Scientific	
**HARDWARE**		
ViiA 7 Real-Time PCR System	ThermoFisher Scientific	

METHODS S1: Methods relating to supplementary material only

Pig Samples

Porcine mesenteric lymph nodes from healthy piglets were kindly provided by The Pirbright Institute, and were processed as for human lymph nodes.

CellDIVE Image Quantification

Segmentation and subsequent extraction of centroid, shape descriptors (e.g., area, perimeter) and all channel intensity information of individual nuclei was performed using StarDist in python with the pre-trained '2d_versatile_flou' model [1] and 'regionprops' from Skicit-image [2], respectively. 249,227 nuclei were detected. Nuclei were assigned a positive marker if the intensity was greater than the median + 1-3.5x the standard deviation for that intensity across all nuclei. More stringent thresholds were assigned to channel CD56 based on weaker staining compared to CD3 or CD20. Only nuclei that were single positive for a given marker were used for downstream analysis. For example, nuclei that were CD3+CD56+ (i.e., considered as NKT cells) were excluded. CD3+ only were defined as T cells, CD20+ cells were defined as B cells and CD56+ cells were defined as NK cells. To separate CD20+ cells inside vs. outside of follicles a distance matrix (i.e., using the Euclidian distance) was produced between all nuclei, and CD20+ cells that exhibited > 15 other CD20+ cells with a 10 µm radius were considered inside a follicle. The (cross) G-function (i.e., cumulative frequency distribution (CFD) of the 1st-order nearest neighbour distances), was used to produce a probability curve for the spatial relationships between indicated cells [3]. The cross G-function is given by:

$$G^{xy}(r) = \frac{1}{N_x}\sum_{k=1}^{N_x} 1\{d_k^{(y)} \leq r\}$$

To provide context, the CFD is compared to a curve based on a Poisson distribution (i.e., a random distribution of cells) within the same given area of the LN (i.e., 15493173 µm^2 in this case). The Poisson distribution is given by:

$$G_{xy}(r) = 1 - e^{-\lambda_y \pi r^2}$$

λ_y is the intensity (mean density) of cell type-y. Clustered populations of cells appear as curves shifted to the left of the Poisson distribution, and is interpreted as cell population x and cell population y being found closer more than you would expect by random for the same given number of cells within a defined area. Curves that match the Poisson distribution are randomly distributed. Curves that fall to the right are likely cells segregated from each other. NK cells and B cells (outside follicles) were further stratified into different density groups i.e., 'sparse', 'low', 'medium', and 'dense' (for NK cells) or density groups 1-10 (1 being sparse and 10 being most dense) for B cells. This was done using quartiles/deciles of probability density values

measured from a gaussian kernel density estimate of the NK or B cell (outside follicle) centroid coordinates. All image processing and quantification was performed using custom scripts written in Python, and are available at DOI 10.5281/zenodo.15396757.

Quantitative real-time polymerase chain reaction (qRT-PCR)

Slices were stored in RNA protect (Qiagen) until RNA extraction by RNeasy Mini Kit (Qiagen) following manufacturer's instructions. An electronic pestle was used to aid tissue disruption. Extracted RNA was reverse transcribed using First-strand cDNA synthesis kit (Thermo Fisher Scientific) and 100ng of complementary DNA used in a 20μl qRT-PCR reaction. PCR cycling consisted of 20 seconds at 95°C followed by 40 cycles of 3 seconds at 95°C followed by 20 seconds at 60°C. Amplified DNA products were detected by SYBR Green PCR Master Mix (ThermoFisher Scientific) and primers for IL1B, IL6 and 18S rRNA and with a ViiA 7 Real-Time PCR System (Thermo Fisher Scientific). The reagents, instruments, and software used are listed in Table S9.

CellPhoneDB

To analyse cell-cell communication molecules, CellPhoneDB v5 (method 3) was used to understand the differentially expressed interactions. Relevant interactions were determined by: 1) interaction genes of interest must be expressed in the corresponding cell type by more than 10% of cells, and 2) at least one gene-cell type pair is identified in the DEG analysis described above. Interactions were also scored as described by CellPhoneDB v5 to rank interactions by specificity of their interacting partners [4,5] . Results were visualised using ktplots. Relevant interactions and significant means are detailed in Table S5.

SUPPLEMENTARY REFERENCES

1. Schmidt, U., Weigert, M., Broaddus, C., and Myers, G. (2018). Cell Detection with Star-Convex Polygons. In Lecture Notes in Computer Science, (Springer International Publishing), pp. 265-273. 10.1007/978-3-030-00934-2_30.
2. van der Walt, S., Schonberger, J.L., Nunez-Iglesias, J., Boulogne, F., Warner, J.D., Yager, N., Gouillart, E., Yu, T., and scikit-image, c. (2014). scikit-image: image processing in Python. PeerJ *2*, e453. 10.7717/peerj.453.
3. Parra, E.R. (2021). Methods to Determine and Analyze the Cellular Spatial Distribution Extracted From Multiplex Immunofluorescence Data to Understand the Tumor Microenvironment. Frontiers in Molecular Biosciences *8*. 10.3389/fmolb.2021.668340.
4. Efremova, M., Vento-Tormo, M., Teichmann, S.A., and Vento-Tormo, R. (2020). CellPhoneDB: inferring cell-cell communication from combined expression of multi-subunit ligand-receptor complexes. Nat Protoc *15*, 1484-1506. 10.1038/s41596-020-0292-x.

5. Troule, K.P., R.; Prete, M.; Cranley, J.; Harasty, A.; Tuong, Z.K.; Teichmann, S.A.; Garcia-Alonso, L.; Vento-Tormo, R. (2023). CellPhoneDB v5: inferring cell-cell communication from single-cell multiomics data. arXiv. 10.48550/arXiv.2311.04567.
